# Studying the Effect of the Host Genetic Background of Juvenile Polyposis Development Using Collaborative Cross and *Smad4* Knock-Out Mouse Models

**DOI:** 10.3390/ijms25115812

**Published:** 2024-05-27

**Authors:** Osayd Zohud, Kareem Midlej, Iqbal M. Lone, Aysar Nashef, Imad Abu-Elnaaj, Fuad A. Iraqi

**Affiliations:** 1 Department of Clinical Microbiology and Immunology, Faculty of Medicine and Health Sciences, Tel-Aviv University, Tel-Aviv 69978, Israel; osaydzohud@mail.tau.ac.il (O.Z.); kareemmidlej@mail.tau.ac.il (K.M.); iqbalzoo84@gmail.com (I.M.L.); 2 Department of Oral and Maxillofacial Surgery, Baruch Padeh Medical Center, Poriya Tebaria 42310, Israel; dr.aysarn@gmail.com (A.N.); iabu@poria.health.gov.il (I.A.-E.); 3 Department of Oral and Maxillofacial Surgery, Meir Medical Center, Faculty of Medicine and Health Sciences, Tel-Aviv University, Kfar-Saba 69978, Israel; 4 Azrieli Faculty of Medicine, Bar-Ilan University, Tsaft 33241, Israel

**Keywords:** juvenile polyposis syndrome, intestinal polyps, intestinal cancer, *Smad4*, collaborative cross mice

## Abstract

Juvenile polyposis syndrome (JPS) is a rare autosomal dominant disorder characterized by multiple juvenile polyps in the gastrointestinal tract, often associated with mutations in genes such as *Smad4* and *BMPR*1*A*. This study explores the impact of *Smad4* knock-out on the development of intestinal polyps using collaborative cross (CC) mice, a genetically diverse model. Our results reveal a significant increase in intestinal polyps in *Smad4* knock-out mice across the entire population, emphasizing the broad influence of *Smad4* on polyposis. Sex-specific analyses demonstrate higher polyp counts in knock-out males and females compared to their WT counterparts, with distinct correlation patterns. Line-specific effects highlight the nuanced response to *Smad4* knock-out, underscoring the importance of genetic variability. Multimorbidity heat maps offer insights into complex relationships between polyp counts, locations, and sizes. Heritability analysis reveals a significant genetic basis for polyp counts and sizes, while machine learning models, including k-nearest neighbors and linear regression, identify key predictors, enhancing our understanding of juvenile polyposis genetics. Overall, this study provides new information on understanding the intricate genetic interplay in the context of *Smad4* knock-out, offering valuable insights that could inform the identification of potential therapeutic targets for juvenile polyposis and related diseases.

## 1. Introduction

Juvenile polyposis syndrome (JPS) is a rare and dominant autosomal disorder characterized by multiple juvenile polyps in the gastrointestinal tract, typically beginning in childhood or adolescence [1]. JPS is caused by mutations in certain genes that regulate the growth of cells in the colon. The most associated genes are *Smad4* and *BMPR*1*A* [2]. *Smad4* is a tumor suppressor gene, and mutations in this gene are the most frequent among cases of JPS. *BMPR1A* is another tumor suppressor gene, and mutations in this gene are less frequent among cases of JPS [3]. *PTEN* is another gene less commonly involved in JPS [3].

JPS is characterized by multiple prominent juvenile polyps in the gastrointestinal tract that can increase the risk of developing colon cancer if not removed [4]. Colorectal cancer (CRC) is one of the most common cancers and the world’s second leading cause of cancer-related death [5]. CRC is caused by the accumulation of genetic and epigenetic changes that transform normal colonic mucosa into adenocarcinoma [6]. Although modern research has shed light on the molecular mechanism of CRC and provided improved screening strategies, the prevalence of CRC continues to increase [5].

Clinically, juvenile polyposis syndrome is diagnosed by having five or more juvenile polyps throughout the gastrointestinal tract or any number of juvenile polyps and a positive family history of juvenile polyposis [4,7].

Most juvenile polyps are hamartomatous but can turn cancerous if not removed [8]. The risk of developing colon cancer in individuals with JPS is estimated to be between 11% and 86%. The risk is higher in individuals with a large number of polyps, polyps located in the proximal (upper) part of the colon, or polyps that are larger in size. Most of this increased risk is attributed to colon cancer, but the stomach, upper gastrointestinal tract, and pancreatic cancers have also been reported [3,9,10,11,12]. Even among patients of the same age, polyp shapes and sizes vary; it is possible to notice this even among members of the same family who have JPS [13].

Some individuals with JPS may have a small number of polyps, while others may have hundreds or even thousands [14]. Some polyps may be small and easily removed during a colonoscopy, while others may be large and require surgery. The size of the polyps is also an essential factor in determining the risk of colon cancer [15]. The specific genetic mutations associated with the disorder can contribute to variations observed in juvenile polyposis syndrome (JPS), affecting the number, size, and location of polyps in the colon. For instance, people with JPS accompanied by *Smad4* gene mutations typically have more polyps than people with JPS accompanied by *BMPR*1*A* gene mutations [9,16].

JPS mouse models (transgenic and knock-out models) have been developed for several genes associated with JPS, including *Smad4*, *BMPR1A*, and *PTEN* [17,18,19]. These models have been used to study the development and progression of polyps in JPS and the potential therapeutic effects of various drugs and treatments [20]. This approach has been used to identify several genetic modifiers for JPS, including genes involved in the Wnt signaling pathway, which regulates cell growth and division [21]. Another pathway identified as a modifier in JPS is the *TGF*-beta signaling pathway [22]. The *TGF*-beta signaling pathway is involved in the regulation of cell growth, differentiation, and apoptosis [22]. Researchers have also identified genetic variants in other pathways, such as the hedgehog signaling pathway and the Notch signaling pathway, as modifiers of JPS; mutations in genes that regulate these pathways are associated with the development of colon cancer [23,24].

Animal models are essential to comprehend the host immune response [25]. The scientific team must determine whether sufficient data to support using an animal model for research, whether ethical concerns are addressed, and whether the information gathered from animal work will significantly advance scientific understanding before selecting an animal model for study [26]. To create the disease state, these models require artificial manipulation of the host and may range from fish to mice [27]. The genetic diversity of collaborative cross (CC) mice, like that of human populations, makes them a powerful tool for biomedical research [28]. CC mice are bred to have a wide range of genetic variation, making them an ideal model for studying complex diseases and traits that are difficult to study using traditional inbred mouse strains [25]. CC mice are produced from intercrossing and outcrossing eight strains, including laboratory and wild-derived strains, resulting in a highly diverse population [29]. CC mice can be used to identify genetic modifiers that modify the effects of disease-causing mutations, making them an effective tool for identifying genetic modifiers that could be related to human disease, providing new therapeutic targets for researchers [29]. CC mice can also investigate complex traits such as behavior and immune system function [30]. Crossing CC mice with KO mice has been a practical approach to identifying genetic modifiers [30]. Here, we present our attempt to identify genetic modifiers for JPS that influence the number and size (categorized based on diameter into A polyps (more than 3 mm), B polyps (1–3 mm), and C polyps (less than 1 mm)) of polyps by crossing CC mice with *Smad4* KO mice.

## 2. Results

### 2.1. The Effect of Smad4 Heterozygous Knock-Out in the General Population

**Figure 1:**
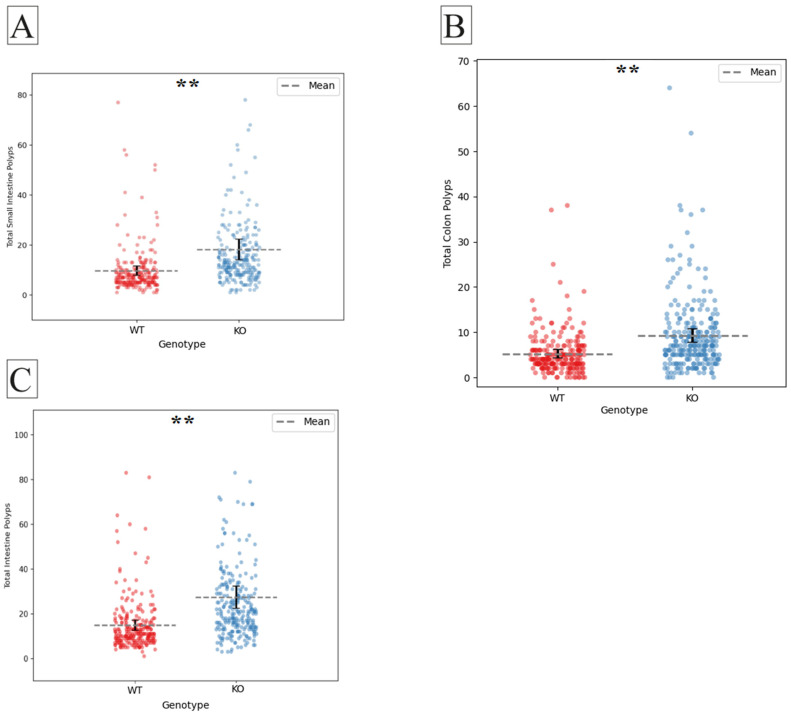
Polyp counts in *Smad4* heterozygous knock-out (KO) and wild-type (WT) mice. (**A**) The mean polyp count in the entire intestine is significantly higher in KO mice compared to WT mice (*p* < 0.001). (**B**) Variation in mean polyp count in the small intestine is significantly elevated in KO mice compared to WT mice (*p* < 0.001). (**C**) The mean polyp count in the colon is markedly higher in KO mice than in WT mice (*p* < 0.001) within the general mouse population. The *X*-axis represents the genotype, while the *Y*-axis represents the number of polyps. The statistical significance of differences in the average number of polyps between the two groups is presented as follows: (**) indicates a highly significant difference at *p* < 0.01.

Our experimental population of F1 mice showed a significant increase in intestinal polyps in *Smad4* heterozygous knock-out mice (n = 260) compared to the wild-type (n = 239), as shown in Figure 1A (*p* < 0.001). This was seen both in the small intestine and the colon, as shown in Figure 1B,C (*p* < 0.001). This matches the previous reports on *Smad4* as a model for intestinal polyposis [31].

### 2.2. Sex Effect

**Figure 2:**
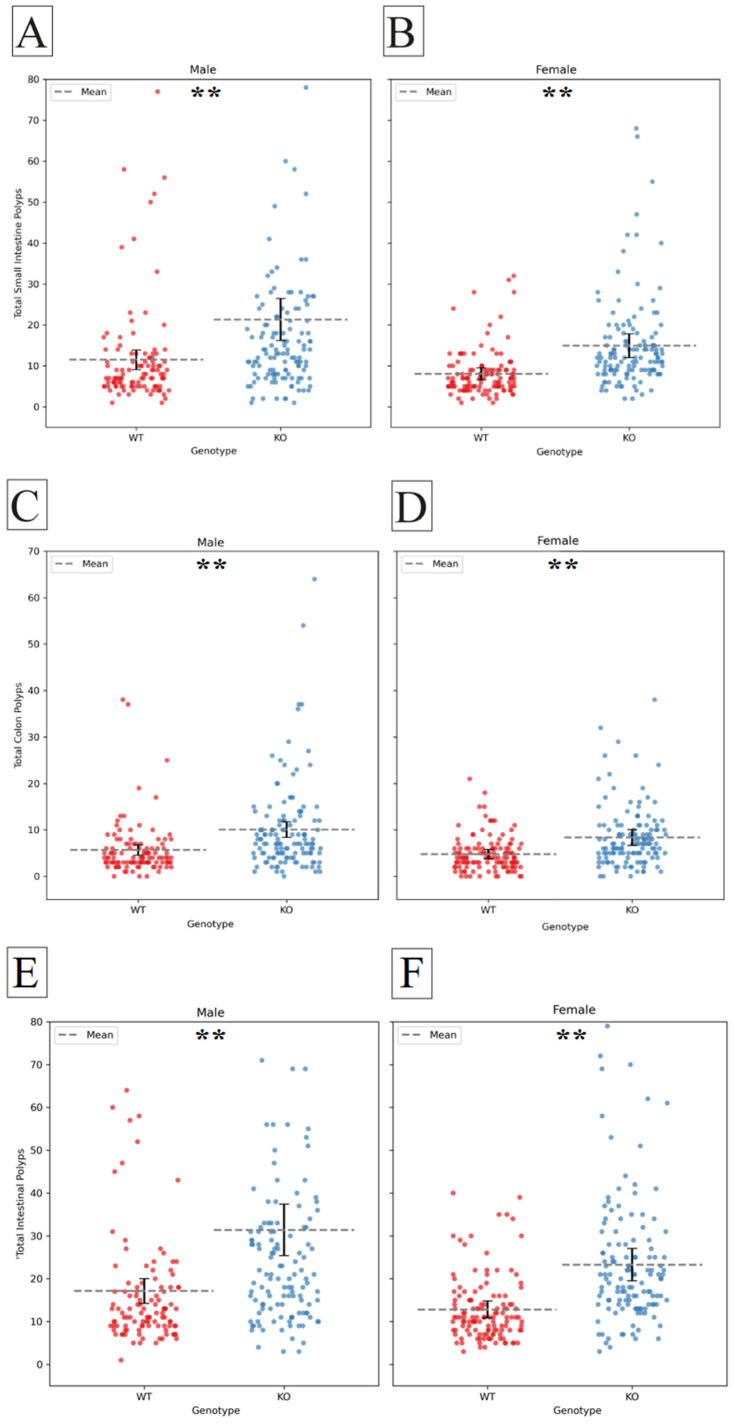
Differential impact of *Smad4* heterozygous knock-out on polyp counts in male and female mice. (**A**) Male mice with *Smad4* heterozygous knock-out exhibit a significant increase in polyp count in the small intestine compared to wild-type mice (*p* < 0.001). (**B**) Female mice with *Smad4* heterozygous knock-out demonstrate a significant increase in polyp count in the small intestine compared to wild-type mice (*p* < 0.001). (**C**) *Smad4* heterozygous knock-out in male mice leads to a significant increase in polyp count in the colon (*p* < 0.001). (**D**) Female mice with *Smad4* heterozygous knock-out show a significant increase in polyp count in the colon compared to wild-type mice (*p* < 0.001). (**E**) *Smad4* heterozygous knock-out in male mice leads to a significant increase in polyp count in the whole intestinal tract (*p* < 0.001). (**F**) Female mice with *Smad4* heterozygous knock-out also show a significant increase in polyp count in the whole intestinal tract compared to wild-type mice (*p* < 0.001). The *X*-axis represents the genotype, while the *Y*-axis represents the number of polyps. Statistical significance of differences in the average number of polyps between the two groups is denoted as follows: (**) indicates a highly significant difference at *p* < 0.01.

The effect of *Smad4* knock-out on polyp counts in males and females was tested separately. Polyp counts significantly increased in KO males and females compared to their WT counterparts in the small intestine (male *p* < 0.001, female *p* < 0.001), the colon (male *p* < 0.001, female *p* < 0.001) (presented in Figure 2), and the whole intestinal tract (male *p* < 0.001, female *p* < 0.001). Male mice tended to have more polyps than females, which is not statistically significant.

While our study specifically focused on mice, observing sex differences in polyp counts prompted consideration of potential implications for human health. One study conducted on a population of individuals with colorectal adenomas reported a higher incidence of polyps in males compared to females [32]. Sex effects played a significant role in colorectal tumorigenesis, mortality, and survival rates. Males exhibited higher incidence rates of CRC throughout their lifetime compared to females, with males also facing higher mortality rates. Additionally, sex-dependent differences extended to screening test willingness, diagnosis stage, survival advantage, site of CRC, metastatic potential, toxicity of anti-cancer drugs, and fiber intake. Understanding these sex-related disparities is crucial for tailoring individualized treatment plans and developing targeted therapies for CRC prevention and management [32].

Other studies did not observe any significant relationships between sex and polyp characteristics in children with non-syndromic juvenile polyps [33]. The analysis focused on factors such as polyp location, volume, and adenomatous transformation, but sex did not show a statistically significant impact on these parameters. This suggests that sex may not have been an essential factor influencing the characteristics of juvenile polyps in this cohort of patients [33].

Further investigation is warranted to elucidate whether similar trends observed in our mouse model may translate to human populations. Understanding the underlying mechanisms driving these sex-specific differences could offer valuable insights into the pathogenesis of intestinal polyps and inform tailored approaches for prevention and treatment strategies in both sexes. Further discussion on the relevance of these findings to human populations would enrich the interpretation of our results.

### 2.3. Line Genetic Effect

**Figure 3:**
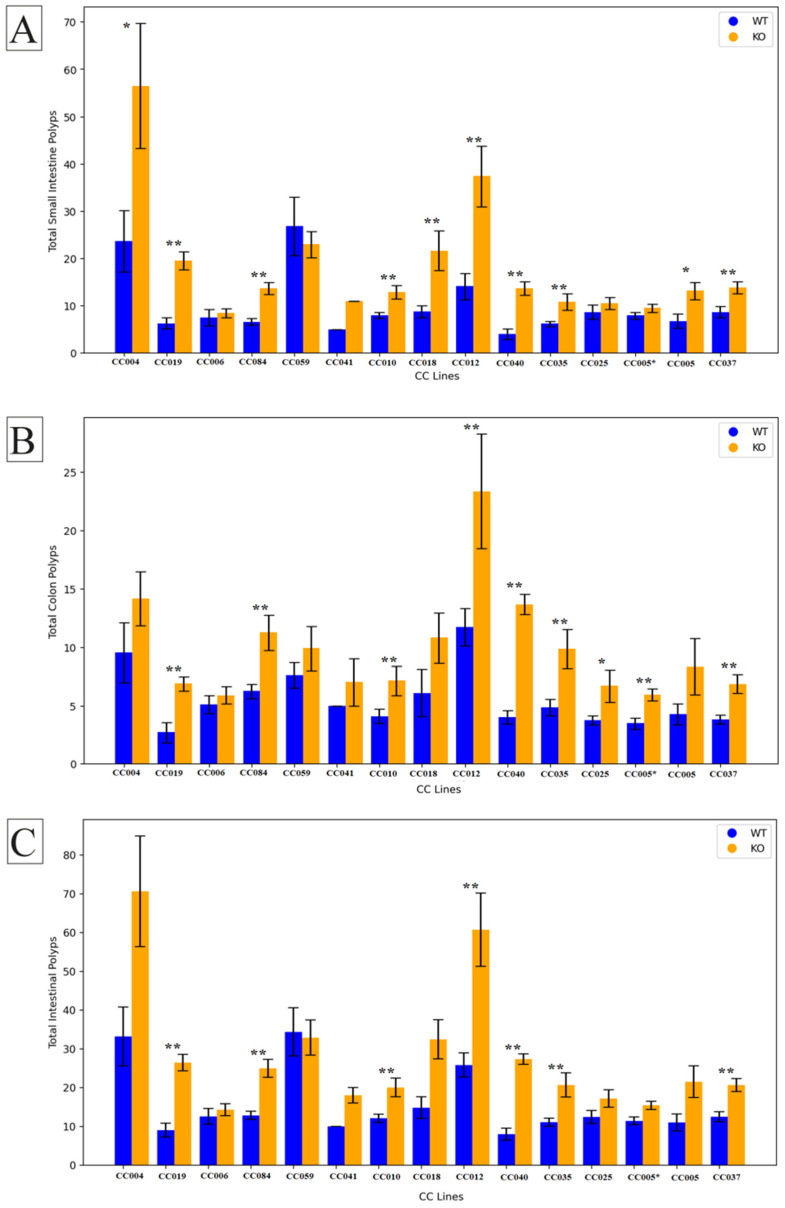
Comparison of polyp counts in F1 CC-C57BL/6 and F1 CC-C57BL/6 J-*Smad4*^tm1Mak^ lines. The average number of polyps (±SE) is shown for 14 F1 CC-C57BL/6 wild-type mice (blue-colored bars) and 14 F1 CC-C57BL/6 J-*Smad4*^tm1Mak^ heterozygous knock-out lines (orange-colored bars). The *X*-axis represents different collaborative cross (CC) lines, while the *Y*-axis shows the number of polyps in (**A**) the small intestine, (**B**) the colon, and (**C**) the entire intestinal tract. Statistical significance of differences in the average number of polyps between the two groups is denoted as follows: (*) indicates a significant difference at *p* < 0.05, and (**) indicates a highly significant difference at *p* < 0.01.

The results of our study reveal the nuanced impact of *Smad4* knock-out across various F1 mouse lines, as depicted in Figure 3. Notably, three lines, CC006, CC059, and CC041, exhibited no statistically significant difference in polyp counts between knock-out (KO) and wild-type (WT) mice in the small intestine. In contrast, lines CC004 (*p* = 0.035), CC005 (*p* = 0.017), and CC018 (*p* = 0.009) demonstrated a significant increase in small intestine polyp counts in the KO group compared to the WT group.

Moreover, lines CC025 (*p* = 0.044) and CC005* (*p* = 0.001) displayed statistically significant elevation in colon polyp counts. Further analysis revealed that in lines CC037 (*p* = 0.001), CC040 (*p* = 0.007), CC019 (*p* < 0.001), CC084 (*p* = 0.000), CC010 (*p* = 0.004), CC012 (*p* = 0.002), and CC035 (*p* = 0.005), there was a significant increase in mean polyp counts in both the small intestine and colon of the KO mice compared to their WT counterparts, CC005 and CC005* are cousin strains formerly stated in our previous works as IL711 and IL6018, respectively [34].

These findings underscore the diverse responses to *Smad4* knock-out among distinct mouse lines, emphasizing the importance of genetic variability in influencing phenotypic outcomes. The observed variations in polyp counts may be attributed to genetic and environmental factors that warrant further investigation. The provided *p*-values indicate the degree of statistical significance, offering valuable insights into the strength of the observed effects. These results contribute to our understanding of the intricate role of *Smad4* in polyp formation, shedding light on potential avenues for future research and therapeutic exploration.

### 2.4. Multimorbidity Heatmaps of Polyp Counts Regarding Body Weight

Understanding the relationship between different physiological variables is crucial for determining the overall health of an organism. Studying the correlation between organ weights and disease pathology is significant in biomedical research. An essential aim of our proposed research was to investigate the effect of host genetic background interaction with *Smad4* knock-out on disease multimorbidity to better understand the coexistence of multiple disease states in different genetic backgrounds. Therefore, in this study, we examined the correlation between the number and size of polyps in the intestines of WT mice and KO mice from various CC lines. We aimed to identify any significant differences in the correlation patterns between the two groups, which may indicate the role of specific genes in developing polyps and related diseases. These traits were converted into heat maps and then used to investigate relationships between trait intensity and transformation. An ideal positive correlation is represented in red (1), while a perfect negative correlation is represented in blue (−1). Our results provide valuable insights into the genetic basis of intestinal polyps and may have implications for developing novel therapies for related diseases.

#### 2.4.1. General Population Correlations

**Figure 4:**
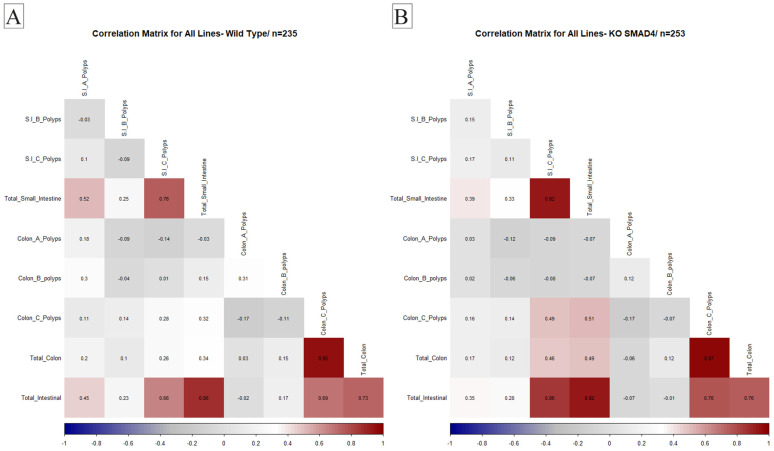
Correlation analysis of polyp development patterns in *Smad4* heterozygous knock-out and wild-type mice populations. This figure presents the correlation analysis results for polyp development within the gastrointestinal tract of wild-type (WT) controls (**A**) compared to *Smad4* heterozygous knock-out (KO) mice (**B**). The analysis reveals a significant positive correlation between the occurrence of Type C polyps in the colon and the presence of polyps in the small intestine within the KO population, suggesting a potential systemic effect or shared susceptibility factors. Furthermore, a marked positive correlation was observed between the total number of Type B polyps in the small intestine and the aggregate count of intestinal polyps in KO mice. This indicates that Type B polyps may be a predominant factor in the overall polyp burden. Additionally, a strong positive association is highlighted between the occurrence of Type B polyps in the small intestine and the total intestinal polyp count in KO mice, underscoring the significance of this polyp subtype in the observed pathology. These findings underscore the complex interplay between different polyp types in the intestines of *Smad4* heterozygous knock-out mice and contribute to our broader understanding of polyp development dynamics in genetic models of intestinal tumorigenesis. The data include correlation coefficients and *p*-values, delineating statistical significance and facilitating a nuanced interpretation of polyp distribution and frequency patterns in relation to genetic modifications.

Our investigation into the KO population compared to WT mice revealed that correlation patterns remained consistent between the two groups. A noteworthy positive correlation was observed between the development of colon C polyps and small intestinal polyps in the KO mice population. Additionally, a positive correlation was identified between total small intestinal B polyps and the overall count of intestinal polyps. This positive association was extended to specifically include small intestinal B polyps, highlighting the interconnected relationship between these variables in *Smad4* knock-out. These findings contribute to our understanding of the complex interactions between different polyp types in knock-out mice, offering insights into potential factors influencing the development of polyps in distinct regions of the intestines. The data illustrating these correlation patterns are presented in Figure 4.

#### 2.4.2. Sex Variation

**Figure 5:**
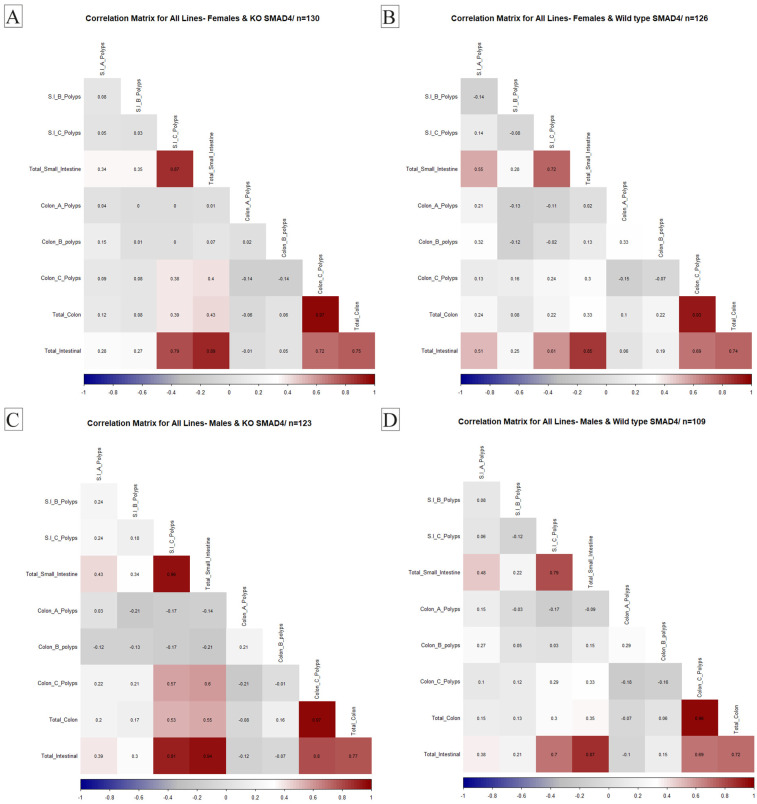
Gender-specific correlation patterns of polyp development in *Smad4* heterozygous knock-out mice compared to wild-type mice. Correlation matrices for (**A**) KO female mouse lines, (**B**) WT female mouse lines, (**C**) KO male mouse lines, and (**D**) WT male mouse lines. In male KO mice, a positive correlation is evident between colon polyps and the C portion of small intestinal polyps. Additionally, a robust positive association exists between counts of total small intestinal B polyps and the overall number of intestinal polyps. In female KO mice, a positive correlation was observed between small intestinal polyps and small intestinal B polyps. Moreover, a positive correlation exists between total colon polyps and small intestinal C polyps. Notably, the previously observed positive correlation between total intestinal polyps and small intestinal A polyps in the KO group was absent in females. These gender-specific correlations provide valuable insights into the effects of *Smad4* knock-out on polyp development, highlighting variations in the relationships between different polyp sizes in male and female mice.

We compared correlation patterns obtained in the KO to the correlations in the WT mice to assess the impact of *Smad4* knock-out. Distinctive correlation patterns emerged in the female subset of knock-out (KO) mice. Notably, a positive correlation was established between small intestinal polyps and small intestinal B polyps, underscoring an interconnected relationship between these variables in *Smad4* knock-out. A positive correlation was also identified between total colon polyps and small intestinal C polyps in female KO mice. However, it is noteworthy that, in the KO group, a previously observed positive correlation between total intestinal polyps and small intestinal A polyps was no longer evident. These gender-specific correlations in females provide valuable insights into the nuanced effects of *Smad4* knock-out on the development of polyps, shedding light on potential variations in the relationships between different polyp sizes. The specific details of these correlations are visually represented in Figure 5A,B. Our investigation revealed specific correlation patterns of particular significance in the male subset of knock-out (KO) mice. A positive correlation was identified between colon polyps and the C portion of small intestinal polyps. Furthermore, a robust positive association persisted in males, linking the counts of total small intestinal B polyps with the overall number of intestinal polyps. The visual representation of these correlations is presented in Figure 5C,D.

Analysis of sex-specific correlation patterns in *Smad4* KO mice provided valuable insights into the effects of *Smad4* knock-out on polyp development, highlighting variations in the relationships between different polyp sizes in male and female mice. Furthermore, the consistent correlation patterns observed within the KO population compared to WT mice underscore the robustness of these relationships despite genetic perturbations. These findings contribute to a deeper understanding of the molecular mechanisms underlying polyp development and may inform future research on therapeutic interventions for juvenile polyposis syndrome.

#### 2.4.3. Polyp Counts Correlations in Different Lines

In our expansive exploration across diverse lines, the impact of *Smad4* knock-out on correlation patterns emerged as a complex and varied interplay influenced by distinct genetic backgrounds. The KO mice from different lines exhibited a range of correlations among various polyp types, revealing a nuanced landscape shaped by underlying genetic factors. The analysis illuminated unique trends in the relationships between polyp counts, providing insight into the intricate genetic interactions influencing the development of intestinal polyps. This line-specific perspective underscores the importance of comprehensively understanding the diverse effects of *Smad4* knock-out, offering valuable insights into the underlying genetic complexities that contribute to the manifestation of polyp-related phenotypes. The comprehensive depiction of these correlations for each line can be found in Supplementary Figures S1–S80, providing a visual representation of the intricate genetic interplay in the context of *Smad4* knock-out across various genetic backgrounds.

### 2.5. Heritability

This study aimed to discover whether polyp counts and size phenotypic variance has a genetic basis in *Smad4* knockout F1 populations. Table 1 summarizes the significant heritability (H2) values calculated to answer this question. One-way ANOVA was used to calculate the heritability of sex and genotype-specific characteristics. The different traits are calculated: total polyp counts in the small intestines and its three segments, SB1, SB2, and SB3, colon polyps. The heritability was calculated for different polyp categories (A, B, and C) based on size for both sexes and genotypes.

### 2.6. Machine Learning

In our study, we had to distinguish between distinct classes (polyp sizes) designated as ‘I’ and ‘II’ in two-class classifications (A and C polyps). Transitioning to three-class classification (A, B, and C polyps) involved distinguishing between ‘I’, ‘II’, and ‘III’. These classifications mirror the complexity observed in the progression of JPS and aid in identifying patterns indicative of disease severity and progression.

In the realm of two-class classification, the linear discriminant analysis (LDA) algorithm exhibited a moderate level of performance in discerning between two distinct classes. As a linear classifier, LDA thrives when confronted with classes characterized by disparate means and akin covariances. Contrarily, the k-nearest neighbors (KNN) algorithm, a non-parametric approach, yielded comparatively inferior results, hinting at a dataset potentially requiring more robust local patterns conducive to its methodology.

On the other hand, support vector machines (SVMs) with a radial basis function (RBF) kernel emerged as the top-performing model among its counterparts, boasting the highest accuracy. The inherent capability of SVMs with an RBF kernel to delineate intricate decision boundaries renders them well-suited for scenarios necessitating nuanced classification. Meanwhile, the random forest (RF) ensemble technique demonstrated commendable yet slightly inferior performance when juxtaposed with SVM. RF harnesses the power of multiple decision trees to amalgamate their predictions, contributing to its competitive performance.

Transitioning to a three-class classification, LDA presented a modest performance level akin to its two-class counterpart, relying on linear decision boundaries to discern between the three classes. Conversely, KNN’s performance suffered a setback, which is indicative of the challenges in capturing distinctions among the three classes. The parameter selection of k in KNN aids in striking a balance between bias and variance, albeit without achieving commensurate performance levels with other models.

In contrast, a SVM with an RBF kernel sustained its supremacy in the three-class scenario, underscoring its adeptness in handling intricate relationships within the data. While random forest continues to deliver respectable results in this expanded classification context, SVM remains the preeminent performer, capitalizing on the ensemble nature of RF to encapsulate the manifold patterns present within the dataset. Detailed results for the regression models are presented in Table 2. Even though our ML assessment provided valuable insights into the genetic patterns behind JPS, its clinical relevance lies in identifying potential disease-modifying genes and pathways. Our findings reveal distinctive patterns indicative of how severe a particular disease is or how it develops, paving the way for targeted interventions and personalized treatment approaches. In future studies, we plan to validate these identified genetic markers in human cohorts and explore their functional significance in disease pathogenesis. ML analysis presents a holistic perspective of JPS genetic underpinnings, exposing potential areas for disease intervention and personalized medicine.

## 3. Discussion

*Smad4* is a protein that is critical in the TGF-beta signaling pathway, which regulates cell growth, differentiation, and apoptosis [35]. *Smad4* knock-out (KO) mice are often used as a model to study the effects of the loss of function of this protein [36]. Our study shows that the impact of *Smad4* KO varies between different collaborative cross mice, which is essential for understanding the genetic complexity of the TGF-beta signaling pathway.

Overall, this study highlights the importance of considering genetic background when studying the effects of gene knock-out and the need for further research to fully understand the genetic basis of intestinal polyps and related diseases. Using the genome-wide association studies (GWAS) approach for modifier screening can provide valuable insights into the genes and pathways involved in these diseases. It can help identify potential therapeutic targets for preventing and treating these diseases.

Linear discriminant analysis (LDA) yielded an accuracy of 0.67 and a Kappa statistic of 0.33, suggesting its effectiveness in distinguishing between classes ‘I’ and ‘II’. The k-nearest neighbors (KNN) model, with an optimal k value of 9, exhibited a lower accuracy of 0.55, possibly indicating challenges in capturing complex relationships. Support vector machines with a radial basis function kernel (SVM-RBF) performed exceptionally well, achieving an accuracy of 0.69 and a Kappa statistic of 0.38, showcasing its robustness in handling non-linear decision boundaries in binary classification tasks in multiclass classification tasks. Linear discriminant analysis (LDA) demonstrated an accuracy of 0.62 and a Kappa statistic of 0.098. The k-nearest neighbors (KNN) model, with an optimal k value of 9, achieved an accuracy of 0.62. Support vector machines (SVM) with optimal parameters C = 0.25 and sigma = 0.0774 provided an accuracy of 0.63, while random forest (RF) achieved an accuracy of 0.62.

However, several limitations to the study must be considered. First, although we tried our best to minimize genetic diversity, it is still possible that the complex interplay between genetics and environment caused disease phenotypes. Also, the application of computational models as a method for data analysis may have missed some chemical reactions occurring in the body or any other effects of factors not included in the model. Therefore, future research should consider including different genetic backgrounds and/or environmental conditions to improve our knowledge of disease mechanisms. There is also a need for experimental confirmation of these genetic modifiers and their corresponding pathways using in vitro and in vivo models, as this will help establish their functional importance. Finally, translating our findings into clinical practice would require validation on human populations to determine whether these can predict disease susceptibility or progression rates. These limitations notwithstanding, further studies will enlighten us on the etiology behind intestinal polyps with the potential to develop specific therapeutics targeting the ailment.

Furthermore, the appearance of polyps later in our study compared to human juvenile polyposis (JP) may be attributed to the genotype of the three wild-derived strains used in producing the CC mice. These strains could express more resistance to JP development, leading to delayed onset of polyp formation. This observation is consistent with findings from the F1 (APC-CC) study conducted by Alexander Dorman et al. [30], where intestinal polyps were observed at five months, two months later than the timeline reported in previous studies [37,38,39]. In the F1 (APC-CC) study, the researchers noted a delay in polyp formation compared to published data, which reported the appearance of intestinal polyps at three months. We hypothesize that similar genetic factors may contribute to the delayed onset of polyp formation observed in our study.

## 4. Materials and Methods

### 4.1. Ethical Aspects of the Project

All animal experiments in this study were compliant with national standards for the care and use of laboratory animals, and the experiment was reviewed and approved by Tel Aviv University’s Institutional Animal Care and Use Committee (IACUC), with an approved number (01-19-044). Mice were monitored daily for their overall health status. Mice that showed loss of around 10% of their BW between two measure points, or 20% overall of their initial body weight, or which were observed to be suffering (less movement and activity) and based on the consultation with the veterinarian at the small animal unit, were terminated.

### 4.2. Generation of F1 Crosses

The CC mouse lines were developed and maintained under conventional environmental conditions at the animal facility of Tel-Aviv University (TAU) by inbreeding for around 20 generations, as described earlier [40]. The C57BL/6 J-*Smad4*^tm1Mak^ mouse line was purchased from the Jackson Laboratory (Bar Harbor, ME, USA).

F1 mice were produced by a cross of females from 20 CC lines available at the Tel-Aviv animal facility with C57BL/6 J-*Smad4*^tm1Mak^ males. After PCR analysis for the *Smad4* gene genotype, 499 F1 mice from 14 lines were identified and included in the study for further assessment and analysis. The mice cohort we used is presented in Table 3.

### 4.3. Mouse Housing and Diet

Mice were housed in the animal facility at the Sackler Faculty of Medicine, Tel-Aviv University (TAU), according to the standard protocol approved by the TAU Animal Use and Care Committee (01-19-044). Mice were housed on hardwood chip bedding in open-topped cages, segregated by sex and CC lineage, maintained under a 12 h light/dark cycle (6:00 a.m. 6:00 p.m.) at 221 °C and fed tap water and standard rodent chow feed ad libitum (TD.2018SC, Teklad Global, Harlan Inc., Madison, WI, USA; contains %Kcal from fat 18%, protein 24%, and carbohydrate 58%) since weaning at three weeks until experiment termination at 80 weeks of age. F1 mice were monitored for their overall health status.

### 4.4. Genomic DNA Extraction and Genotyping

The NaOH extraction method was used to extract genomic DNA, as referenced in [41]. In the DNA preparation process, 3–4 mm pieces of the tail were trimmed and then placed into an Eppendorf tube. Subsequently, a solution comprising 75 μL of 25 NaOH and 0.2 mM EDTA was added to each sample. The samples were then meticulously placed within a thermocycler and subjected to a temperature of 98 °C for a duration of 1 h, after which the temperature was lowered to 15 °C and maintained at this level until the subsequent steps. Following the thermal treatment, 75 μL of a 40 mM Tris HCl solution with a pH of 5.5 were precisely added to the samples. To separate the components, the samples were centrifuged at 4000 rpm for a duration of 3 min. Finally, aliquots were extracted from the samples for PCR analysis.

### 4.5. Genotyping of F1 Mice

Mice were genotyped using a PCR protocol employing specific sets of primers. The primer sets utilized were as follows:

Primer 30403 (5′-TGT AGT TCT GTC TTT CCT TCC TG-3′);

Primer 30404 (5′-ACT GAC CTT TAT ATA CGC GCT TG-3′);

Primer oIMR2088 (5′-AGA CTG CCT TGG GAA AAG CG-3′).

PCR genotyping involved two distinct reactions denoted as Reaction A and Reaction B:

Reaction A: Primers 30403 and 30404 were employed to amplify a specific 200 bp segment from the wild-type (WT) copy of the *Smad4* gene.

Reaction B: Primers 30404 and oIMR2088 were used to generate a PCR 300bp product indicative of the knock-out (KO) *Smad4* genotype.

Both reactions constituted a touchdown phase. Afterward, the PCR resumed with denaturation at 94.0 °C, annealing at 60.0 °C, and extension at 72.0 °C for 30 cycles. Finally, an extension step was conducted at 72.0 °C, followed by a hold step at 10.0 °C.

### 4.6. Tissue Collection

At the time of termination (80 weeks of age), the F1 mice were terminated and culled using CO_2_ protocol. The body weight of the mouse was recorded as the final body weight. This was used to calculate body weight change during the experiment using the following formula: body weight change = (final body weight − initial body weight) × 100%/final body weight. The small intestine and colon were extracted and washed with PBS. The small intestine was divided into segments (SB1-proximal, SB2-middle, and SB3-distal), and the colon was kept as a whole. All segments were spread on 3 mm Whatman cellulose filter papers [34].

### 4.7. Intestine Whole Mounts Preparation and Assessing the Intestinal Polyp Counts

**Figure 6:**
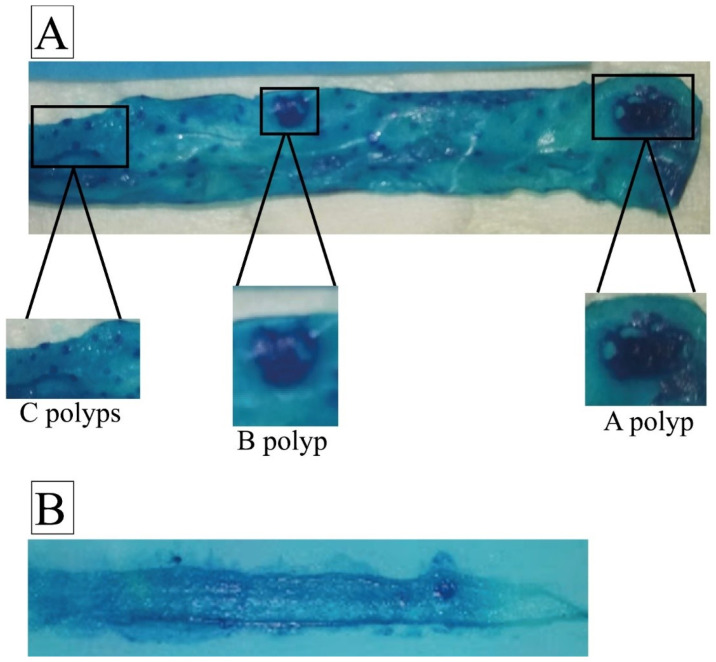
Representative whole mounts of mouse intestine highlighting polyp classification. (**A**) Microscope’s magnification of the entire mounts stained to visualize polyps in a mouse with heterozygous *Smad4* knock-out genotype. Depending on size criteria, multiple polyps are visible and categorized into three classes (A, B, and C). (**B**) Whole mount from a mouse with the wild-type genotype, showing fewer polyps in the same intestinal segment compared to the *Smad4* knock-out mouse. The microscope’s magnification was 10×.

Intestines were fixed in 10% neutral buffered formalin (NBF) overnight and stained using 0.02% methylene blue, as described earlier [42]. A magnifying glass lens was used to examine the stained intestinal and colon samples. Polyps were counted and categorized based on size, including those measuring greater than 3 mm (A polyps), 1–3 mm (B polyps), and less than 1 mm (C polyps). Figure 6 visually represents the polyps observed during this experimental assessment.

### 4.8. Estimation of the Heritability of the Assessed Phenotypes

Heritability measures the fraction of phenotype variability attributed to genetic variation [43]. Here, we used the ANOVA results to calculate the broad-sense heritability using the formula below:

H2=Vg/(Vg+Ve),

where H2 is the heritability, Vg is the genetic variance between the CC lines, and Ve is the environment variance. Considering the heritability results, we calculated the genetic coefficient of variation (CVg), which indicates the absolute amount of genetic variation. The CVg was calculated using the standard deviation (SD) results among the CC lines and trait mean overall CC.

CVg=SD/Mean.

### 4.9. Statistical Analysis

Data analysis was performed using a statistical software package, IBM SPSS statistic 23. An independent sample *t*-test was carried out to determine if there was a significant difference in polyp counts between the whole population of KO mice compared with WT mice. The difference in polyp counts between different genotypes in the male and female cohorts was tested for the sex effect. Finally, the difference in mean polyp counts was tested among WT and KO mice in different lines to measure the line effect. The Pearson product-moment correlation coefficient was used to measure the correlation between traits (polyp counts, organ %weights, and body weight changes).

### 4.10. Machine Learning

Incorporating machine learning (ML) into the analysis of juvenile polyposis syndrome (JPS) provided a robust method for understanding the intricate relationships between genetic factors and polyp counts in the collaborative cross (CC) mouse model. The ML pipeline involves data preprocessing, model application, and performance evaluation. The analysis was conducted on a dataset comprising 304 samples, each characterized by 12 predictor variables. The response variable had three classes denoted as ‘I’, ‘II’, and ‘III’ in one analysis while denoted as ‘I’ and ‘II’ in the second. The dataset included information related to mouse characteristics, polyp counts, sizes, body weights at different time points, and various other features.

### 4.11. Data Preprocessing

Before model training, the dataset was examined for missing values and outliers. Any necessary data cleaning or imputation was performed to ensure the integrity of the dataset. The dataset underwent preprocessing steps, including centering and scaling the ten predictor variables. This was carried out to standardize the features and enhance the performance of specific algorithms.

Descriptive statistics were computed to gain insights into the distribution and characteristics of the dataset. This involved calculating summary statistics such as mean, median, minimum, maximum, and quartiles for continuous variables and frequency distributions for categorical variables.

Classification algorithms:

Several machine-learning classification algorithms were employed to predict the class labels of the samples [44]. The primary algorithms used were as follows:

(a) Linear discriminant analysis (LDA):

LDA is a linear classification technique that aims to determine a linear combination of predictors that best separates the classes [44,45].

(b) k-nearest neighbors (KNN):

KNN is a non-parametric algorithm that classifies a data point based on the majority class of its k-nearest neighbors in the feature space.

(c) Support vector machines with a radial basis function kernel (SVM-RBF):

SVM with an RBF kernel is a powerful algorithm for non-linear classification. The hyperparameters C and sigma were tuned to optimize model performance.

(d) Random forest (RF):

RF is an ensemble learning method that constructs a multitude of decision trees during training and outputs the class, that is, the mode of the classes (classification) of the individual trees [46]. Additionally, RF is based on bagging and plays an important role in ensemble ML [46]. RF has been implemented vastly in biomedicine research [47,48]. In this study, we used the “rf” default implementation for RF with 100 trees. Additionally, in this model, RMSE was used to select the optimal model using the smallest value.

### 4.12. Model Evaluation

A robust evaluation process was implemented to gauge the models’ predictive capabilities. A 70–30% train-test split ensured unbiased evaluations of unseen data. Key metrics, including RMSE, R-squared, and mean absolute error (MAE), were employed for comprehensive performance analysis [49].

## 5. Conclusions

In conclusion, our study delved into the intricate genetic interplay associated with *Smad4* knock-out in juvenile polyposis syndrome (JPS) using collaborative cross (CC) mice, a genetically diverse model. Our findings revealed a significant increase in intestinal polyps in *Smad4* knock-out mice across the entire population, emphasizing the broad influence of *Smad4* on polyposis. Sex-specific analyses with distinct correlation patterns demonstrated higher polyp counts in knock-out males and females. Line-specific effects highlighted the nuanced response to *Smad4* knock-out, underscoring the importance of genetic variability. The heritability analysis underscored a significant genetic basis for polyp counts and sizes, reaffirming the importance of considering genetic background when studying the effects of gene knock-out. Machine learning models identified key predictors, including k-nearest neighbors and linear regression, enhancing our understanding of juvenile polyposis genetics. Our comprehensive investigation extended to multimorbidity heat maps, revealing complex relationships between polyp counts, locations, and sizes. The correlation patterns provide valuable insights into the interconnected nature of different polyp types, shedding light on potential factors influencing their development in distinct regions of the intestines. Moreover, our study explored the impact of *Smad4* knock-out across various CC mouse lines, highlighting diverse responses and emphasizing the need to consider genetic variability in influencing phenotypic outcomes. The observed variations in polyp counts may result from a combination of genetic and environmental factors that warrant further investigation.

Machine learning analysis, employing linear discriminant analysis, k-nearest neighbors, support vector machines, and random forest, adds a predictive dimension to our understanding. These models showcase varying accuracies in classifying different polyp categories, reinforcing the complexity of the genetic landscape in the context of *Smad4* knock-out. Our study provides a comprehensive understanding of the intricate genetic factors at play in *Smad4* knock-out, offering valuable insights into potential therapeutic targets for juvenile polyposis and related diseases. The consideration of genetic variability, as highlighted throughout our research, underscores the importance of personalized and precise approaches in addressing the complexities of polyposis syndromes. Further research into specific genes and signaling pathways involved in these diseases from various genetic backgrounds could pave the way for innovative therapies and preventive strategies.

## Figures and Tables

**Table 1:** Results of calculating heritability (H2) values. Heritability was calculated using one-way ANOVA for the traits in our study, which were calculated separately by sex and genotype.

Sex	Genotype	Trait	df between	df within	n	MS between	MS within	VG	H2	Trait Mean	CVg	Anova Sig
Female	WT	SB1_C	12	96	7.46	14.04	3.11	1.47	0.32	1.71	0.71	0.000
Female	WT	SB2_A	12	96	7.46	1.31	0.70	0.08	0.11	0.66	0.43	0.046
Female	WT	SB2_B	12	96	7.46	0.49	0.41	0.01	0.03	0.52	0.20	0.298
Female	WT	SB3_A	12	96	7.46	1.92	0.86	0.14	0.14	0.80	0.47	0.016
Female	WT	SB3_C	12	96	7.46	26.55	7.09	2.61	0.27	1.70	0.95	0.000
Female	WT	S.I_A_Polyps	12	96	7.46	7.21	3.55	0.49	0.12	2.04	0.34	0.029
Female	WT	S.I_C_Polyps	12	96	7.46	68.87	18.15	6.80	0.27	4.59	0.57	0.000
Female	WT	Total_Small_Intestine	12	96	7.46	96.44	26.12	9.42	0.27	8.48	0.36	0.000
Female	WT	Colon_A_Polyps	12	96	7.46	0.15	0.08	0.01	0.10	0.10	0.96	0.054
Female	WT	Colon_B_polyps	12	96	7.46	0.70	0.52	0.02	0.04	0.40	0.39	0.204
Female	WT	Colon_C_Polyps	12	96	7.46	50.97	9.60	5.54	0.37	4.64	0.51	0.000
Female	WT	Total_Colon	12	96	7.46	46.08	9.92	4.85	0.33	5.15	0.43	0.000
Female	WT	Total_Intestinal	12	96	7.46	191.35	43.68	19.79	0.31	13.62	0.33	0.000
Female	KO	SB1_A	13	100	7.21	0.96	0.85	0.02	0.02	0.87	0.14	0.340
Female	KO	SB1_B	13	100	7.21	0.77	0.74	0.00	0.01	0.92	0.07	0.417
Female	KO	SB1_C	13	100	7.21	43.26	29.78	1.87	0.06	3.37	0.41	0.149
Female	KO	SB2_A	13	100	7.21	0.93	0.84	0.01	0.02	1.00	0.11	0.358
Female	KO	SB2_C	13	100	7.21	16.22	7.22	1.25	0.15	2.39	0.47	0.013
Female	KO	SB3_A	13	100	7.21	2.14	1.00	0.16	0.14	1.20	0.33	0.018
Female	KO	SB3_C	13	100	7.21	118.77	36.60	11.39	0.24	3.96	0.85	0.000
Female	KO	S.I_A_Polyps	13	100	7.21	5.74	3.05	0.37	0.11	3.07	0.20	0.041
Female	KO	S.I_B_Polyps	13	100	7.21	3.35	3.00	0.05	0.02	2.57	0.09	0.355
Female	KO	S.I_C_Polyps	13	100	7.21	348.49	88.36	36.06	0.29	9.73	0.62	0.000
Female	KO	Total_Small_Intestine	13	100	7.21	394.98	92.78	41.89	0.31	15.37	0.42	0.000
Female	KO	Colon_B_polyps	13	100	7.21	1.90	0.88	0.14	0.14	0.62	0.60	0.017
Female	KO	Colon_C_Polyps	13	100	7.21	87.11	39.20	6.64	0.14	8.23	0.31	0.014
Female	KO	Total_Colon	13	100	7.21	81.18	38.90	5.86	0.13	8.96	0.27	0.021
Female	KO	Total_Intestinal	13	100	7.21	650.63	153.55	68.90	0.31	24.33	0.34	0.000
Male	WT	SB1_A	13	87	6.29	0.83	0.60	0.04	0.06	0.50	0.38	0.187
Male	WT	SB1_C	13	87	6.29	10.50	4.77	0.91	0.16	1.64	0.58	0.016
Male	WT	SB2_A	13	87	6.29	1.77	0.76	0.16	0.17	0.74	0.54	0.011
Male	WT	SB2_C	13	87	6.29	35.20	15.05	3.21	0.18	2.21	0.81	0.010
Male	WT	SB3_B	13	87	6.29	0.90	0.81	0.01	0.02	0.76	0.16	0.359
Male	WT	SB3_C	13	87	6.29	220.63	92.31	20.41	0.18	3.84	1.18	0.009
Male	WT	S.I_A_Polyps	13	87	6.29	5.49	3.48	0.32	0.08	1.98	0.29	0.107
Male	WT	S.I_C_Polyps	13	87	6.29	380.31	123.12	40.92	0.25	7.69	0.83	0.001
Male	WT	Total_Small_Intestine	13	87	6.29	445.20	122.28	51.37	0.30	11.67	0.61	0.000
Male	WT	Colon_A_Polyps	13	87	6.29	0.08	0.07	0.00	0.01	0.08	0.35	0.400
Male	WT	Colon_B_polyps	13	87	6.29	0.94	0.46	0.08	0.14	0.39	0.72	0.025
Male	WT	Colon_C_Polyps	13	87	6.29	72.84	28.17	7.11	0.20	5.38	0.50	0.004
Male	WT	Total_Colon	13	87	6.29	68.33	30.91	5.95	0.16	5.84	0.42	0.015
Male	WT	Total_Intestinal	13	87	6.29	699.96	172.11	83.98	0.33	17.51	0.52	0.000
Male	KO	SB1_A	13	95	6.86	2.07	1.12	0.14	0.11	1.19	0.31	0.046

**Table 2:** Summary of machine learning classification models tested, featuring linear discriminant analysis (LDA), support vector machines (SVMs), K-nearest neighbors, and random forest (RF) in two-class and three-class classifications.

Two Classes				
	LDA	KNN	SVM	RF
Accuracy	0.67	0.55	0.69	0.64
Kappa	0.33	0.1	0.38	0.27
**Three Classes**				
Accuracy	0.62	0.62	0.63	0.62
Kappa	0.1	0.07	0.0	0.1

**Table 3:** Summary of the sample size of male and female mice used from the 14 different lines of the collaborative cross mouse population.

F1 (Samd4^KO^ X CCxxx)			Sex		Total
			F	M	
CC037	Genotype	WT	17	15	32
		KO	14	19	33
	Total		31	34	65
CC004	Genotype	WT	4	5	9
		KO	11	8	19
	Total		15	13	28
CC040	Genotype	WT	1	2	3
		KO	1	2	3
	Total		2	4	6
CC005	Genotype	WT	4	4	8
		KO	3	3	6
	Total		7	7	14
CC019	Genotype	WT	4	3	7
		KO	12	4	16
	Total		16	7	23
CC006	Genotype	WT	7	5	12
		KO	10	7	17
	Total		17	12	29
CC084	Genotype	WT	12	12	24
		KO	13	11	24
	Total		25	23	48
CC059	Genotype	WT	4	9	13
		KO	4	6	10
	Total		8	15	23
CC041	Genotype	WT	2	0	2
		KO	2	0	2
	Total		4		4
CC010	Genotype	WT	11	12	23
		KO	14	10	24
	Total		25	22	47
CC018	Genotype	WT	16	8	24
		KO	14	15	29
	Total		30	23	53
CC012	Genotype	WT	7	4	11
		KO	5	6	11
	Total		12	10	22
CC035	Genotype	WT	11	8	19
		KO	5	13	18
	Total		16	21	37
CC025	Genotype	WT	9	11	20
		KO	8	11	19
	Total		17	22	39
CC005	Genotype	WT	20	12	32
		KO	15	14	29
	Total		35	26	61
Total	Genotype	WT	129	110	239
		KO	131	129	260
	Total		260	239	499

## Data Availability

Data are contained within the article.

## Supplementary Materials

The following supporting information can be downloaded at https://www.mdpi.com/article/10.3390/ijms25115812/s1.
