# Supplementary material for: Studying the Effect of the Host Genetic Background of Juvenile Polyposis Development Using Collaborative Cross and Smad4 Knock-Out Mouse Models
Source: Int J Mol Sci. 2024 May 27;25(11):5812. doi: 10.3390/ijms25115812 (PMC11172477; doi:10.3390/ijms25115812)
Supplement: Supplementary file 1 [file ijms-25-05812-s001.zip › ijms-2962380-supplementary.pdf]

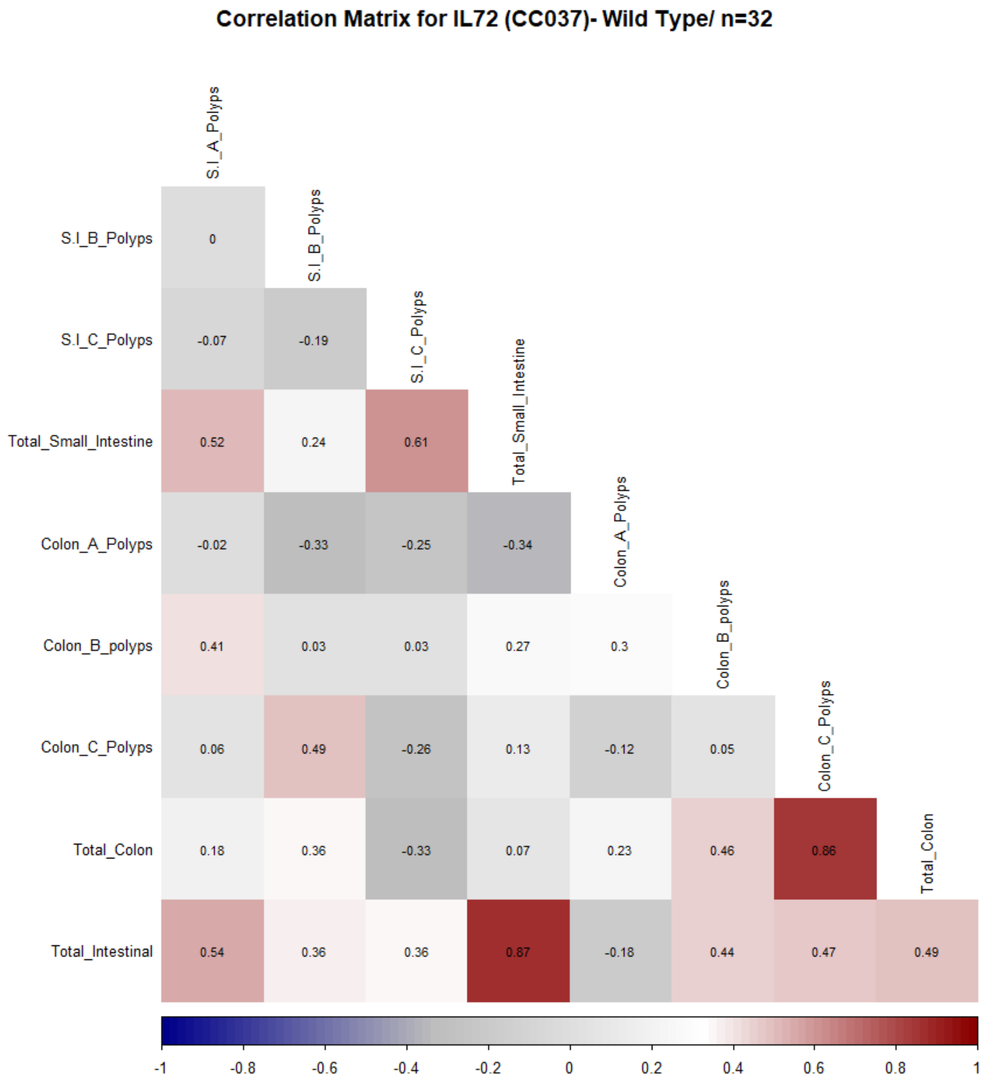

Supplementary Figure S1 Correlation Analysis of Polyp Development Patterns in Wild-Type Mice from line CC037.

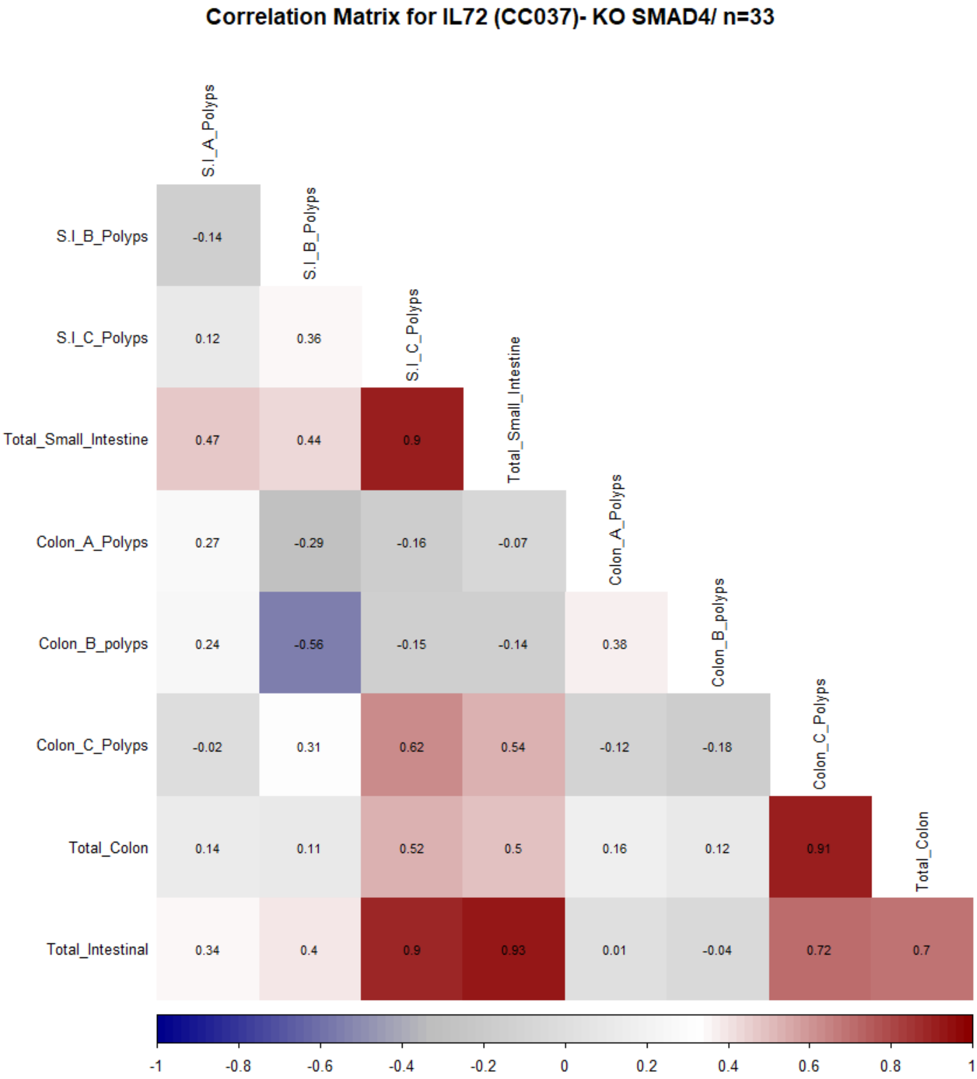

Supplementary Figure S2 Correlation Analysis of Polyp Development Patterns in HETEROZYGOUS KNOCK-OUT Mice from line CC037.

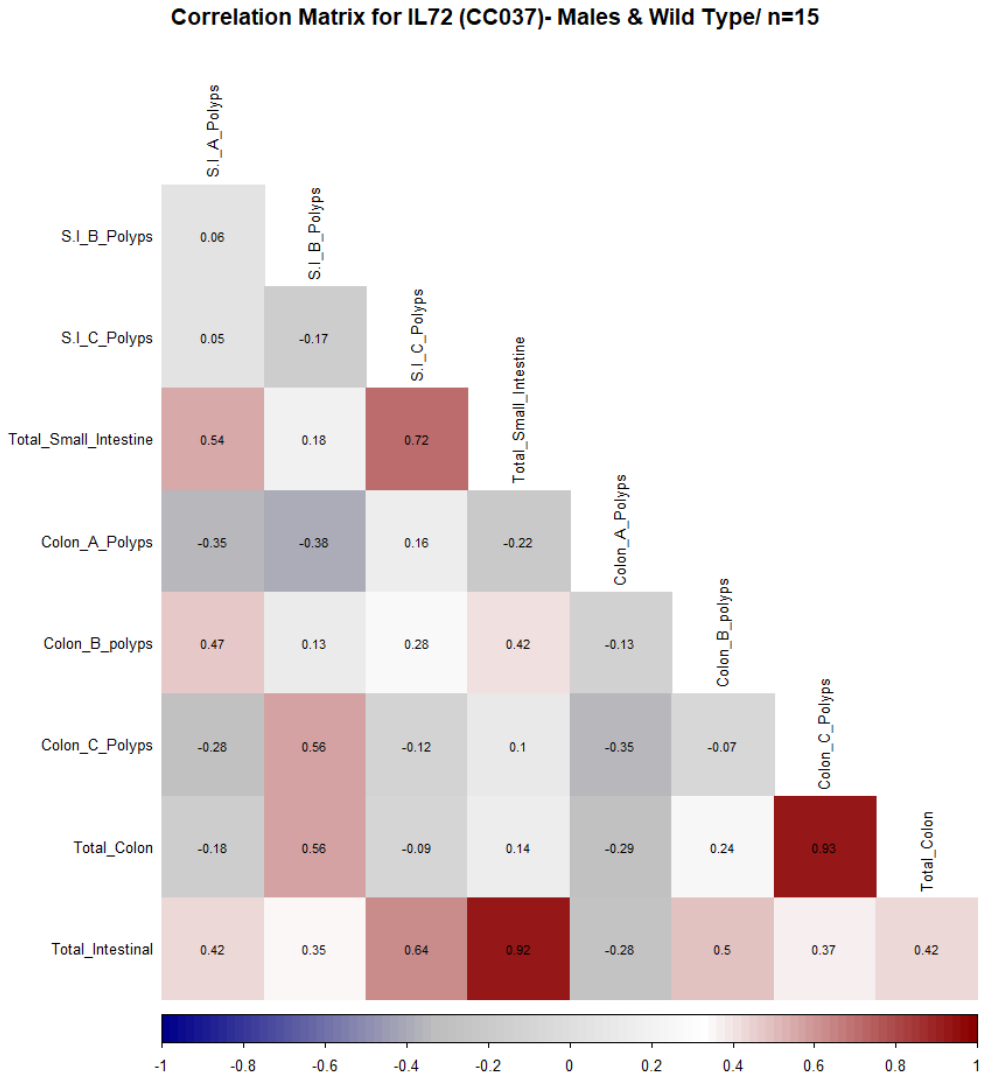

Supplementary Figure S3 Correlation Analysis of Polyp Development Patterns in male Wild-Type Mice from line CC037.

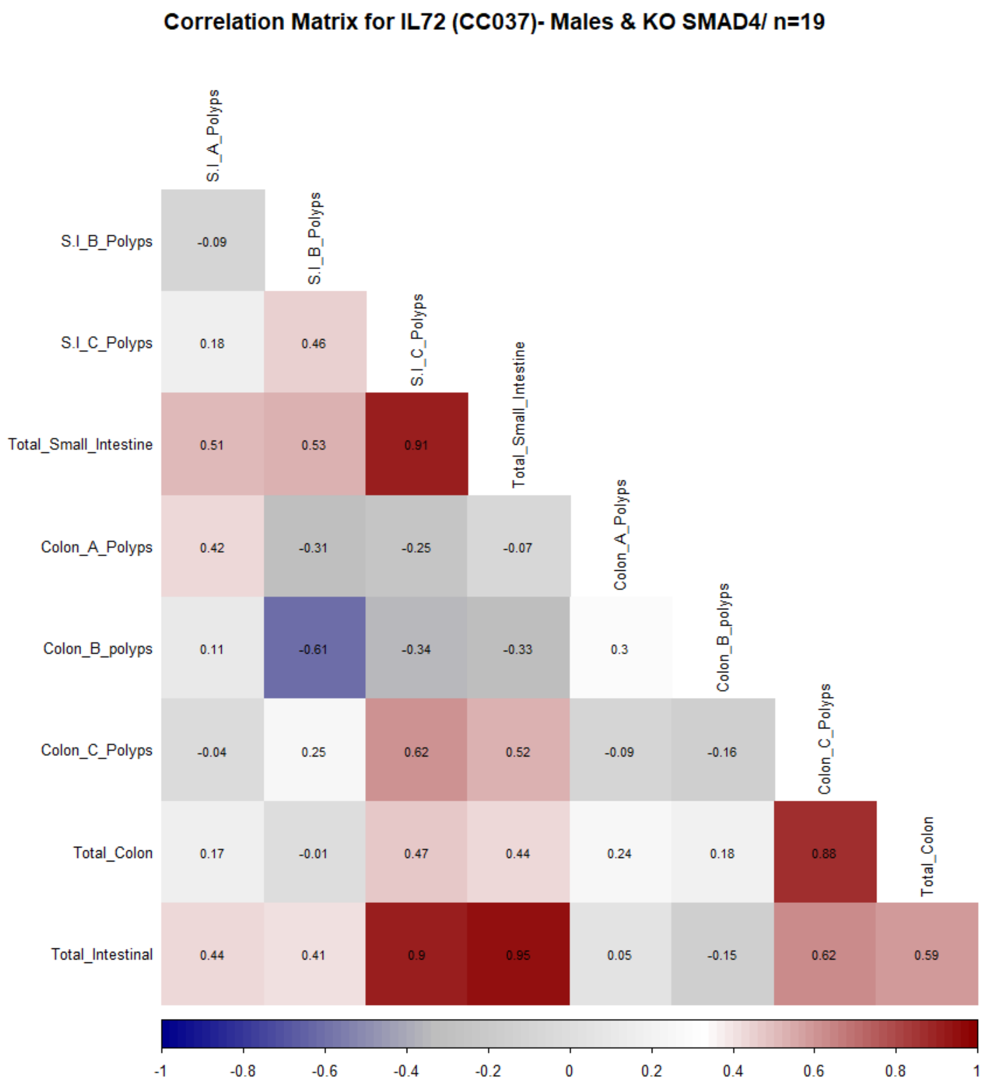

Supplementary Figure S4 Correlation Analysis of Polyp Development Patterns in HETEROZYGOUS KNOCK-OUT male Mice from line CC037.

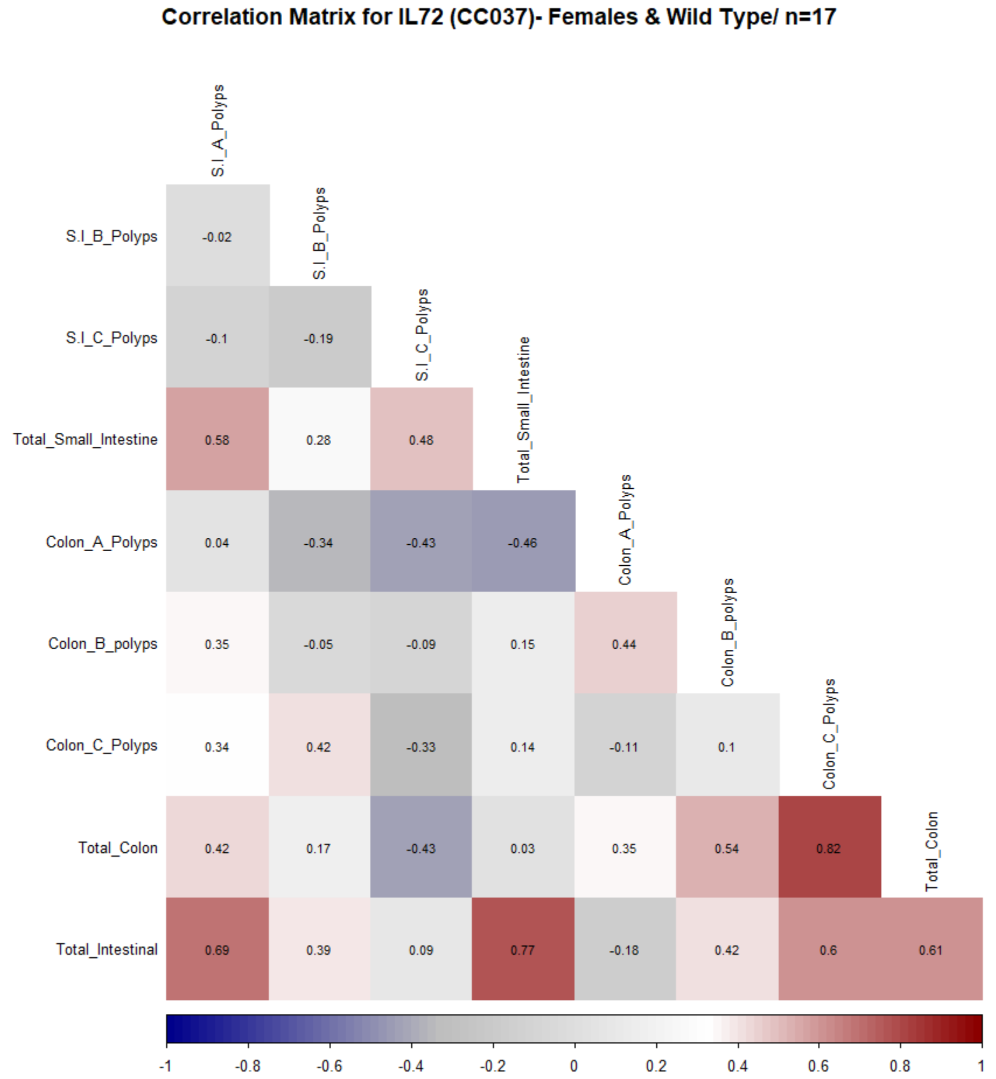

Supplementary Figure S5 Correlation Analysis of Polyp Development Patterns in female Wild-Type Mice from line CC037.

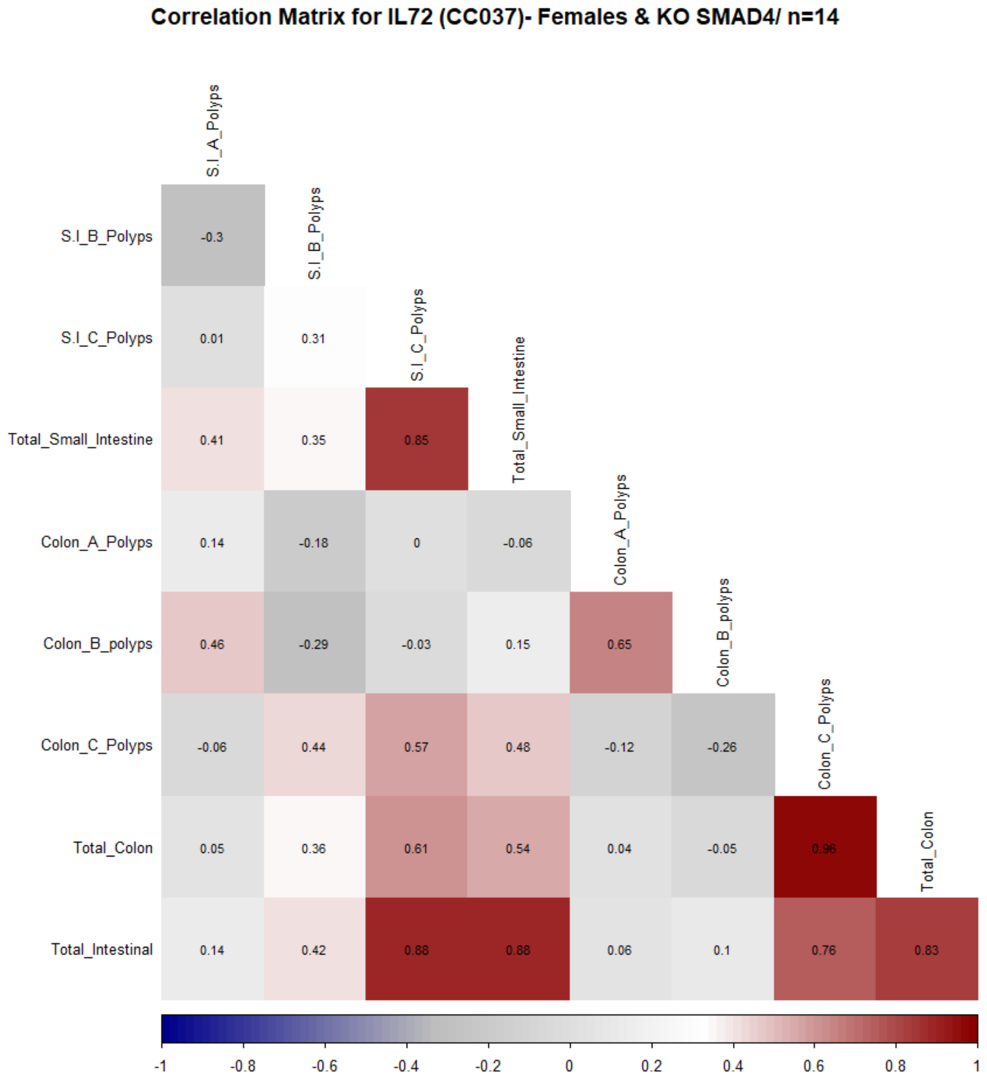

Supplementary Figure S6 Correlation Analysis of Polyp Development Patterns in female HETEROZYGOUS KNOCK-OUT Mice from line CC037.

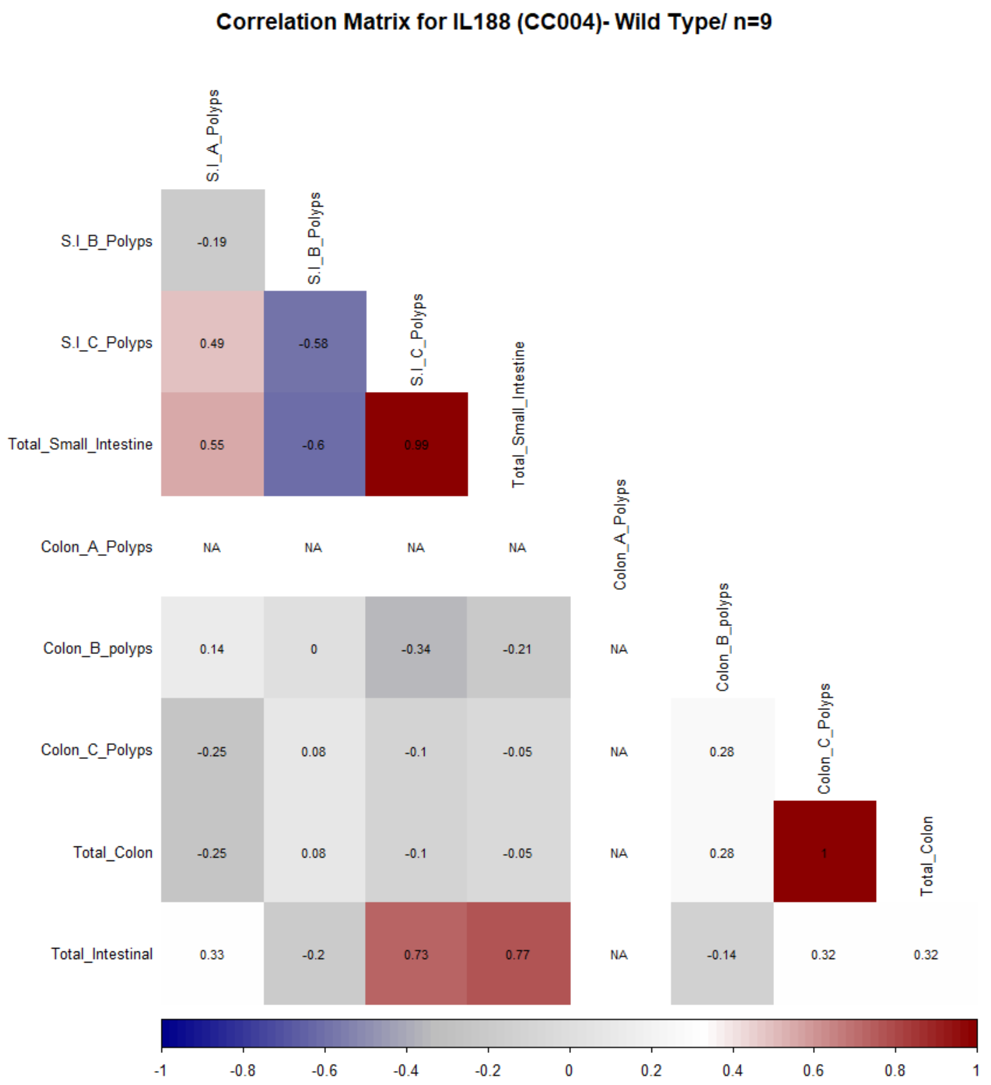

Supplementary Figure S7 Correlation Analysis of Polyp Development Patterns in Wild-Type Mice from line CC004.

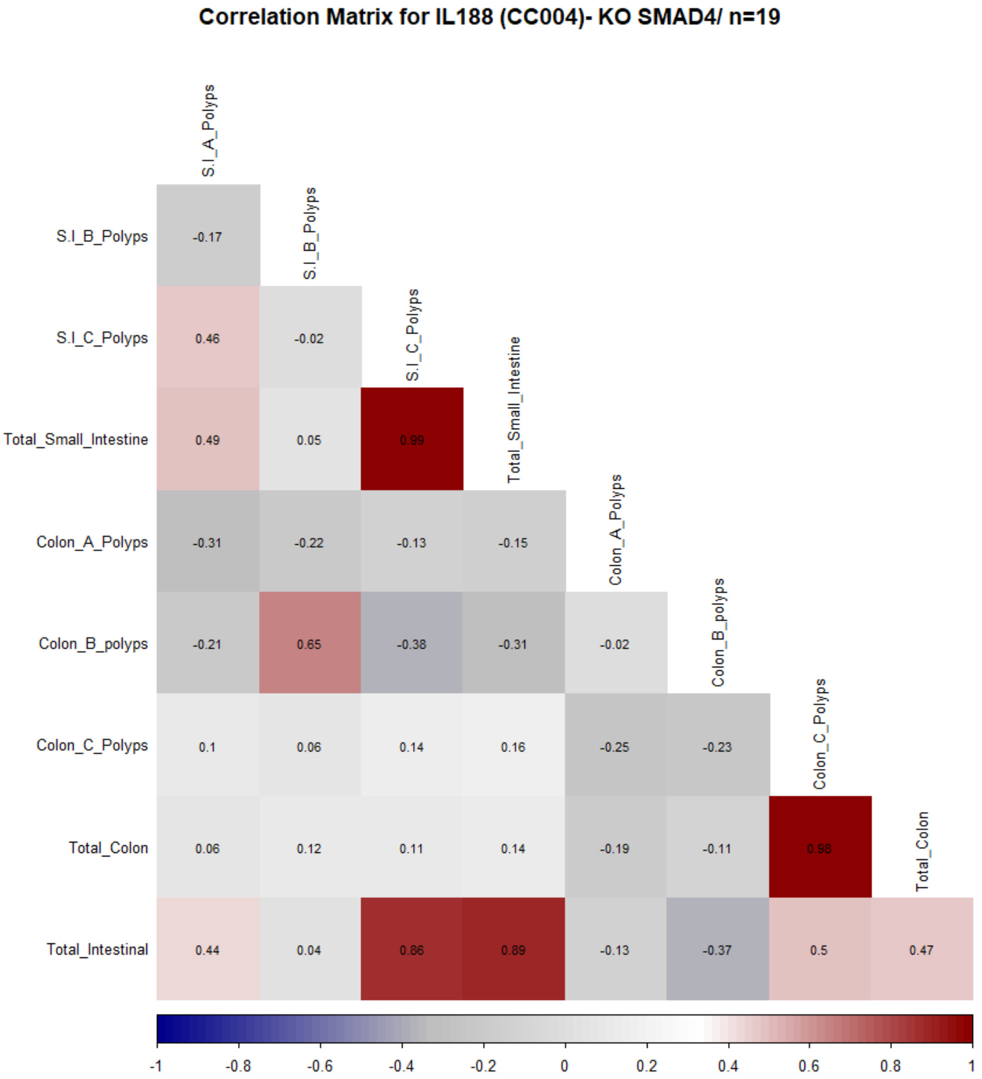

Supplementary Figure S8 Correlation Analysis of Polyp Development Patterns in HETEROZYGOUS KNOCK-OUT Mice from line CC004.

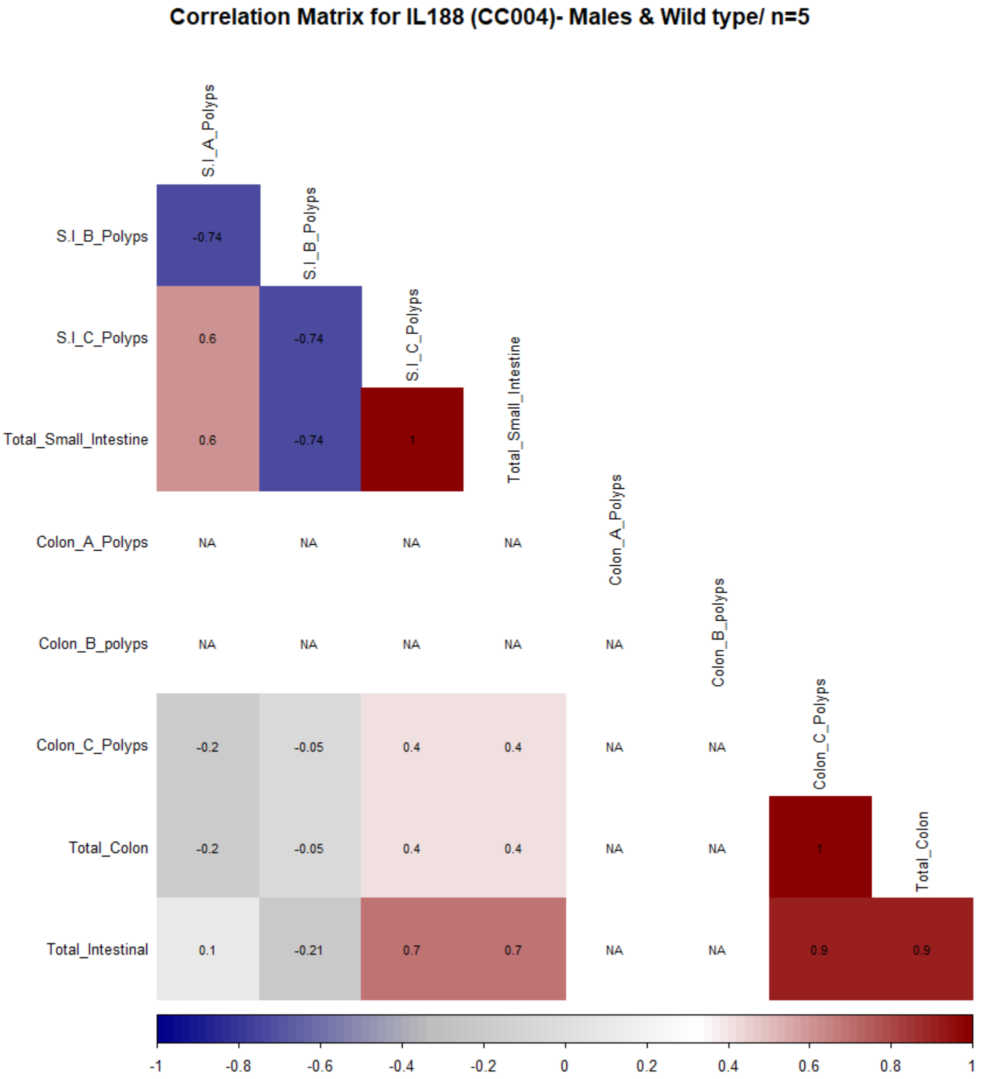

Supplementary Figure S9 Correlation Analysis of Polyp Development Patterns in Wild-Type male Mice from line CC004.

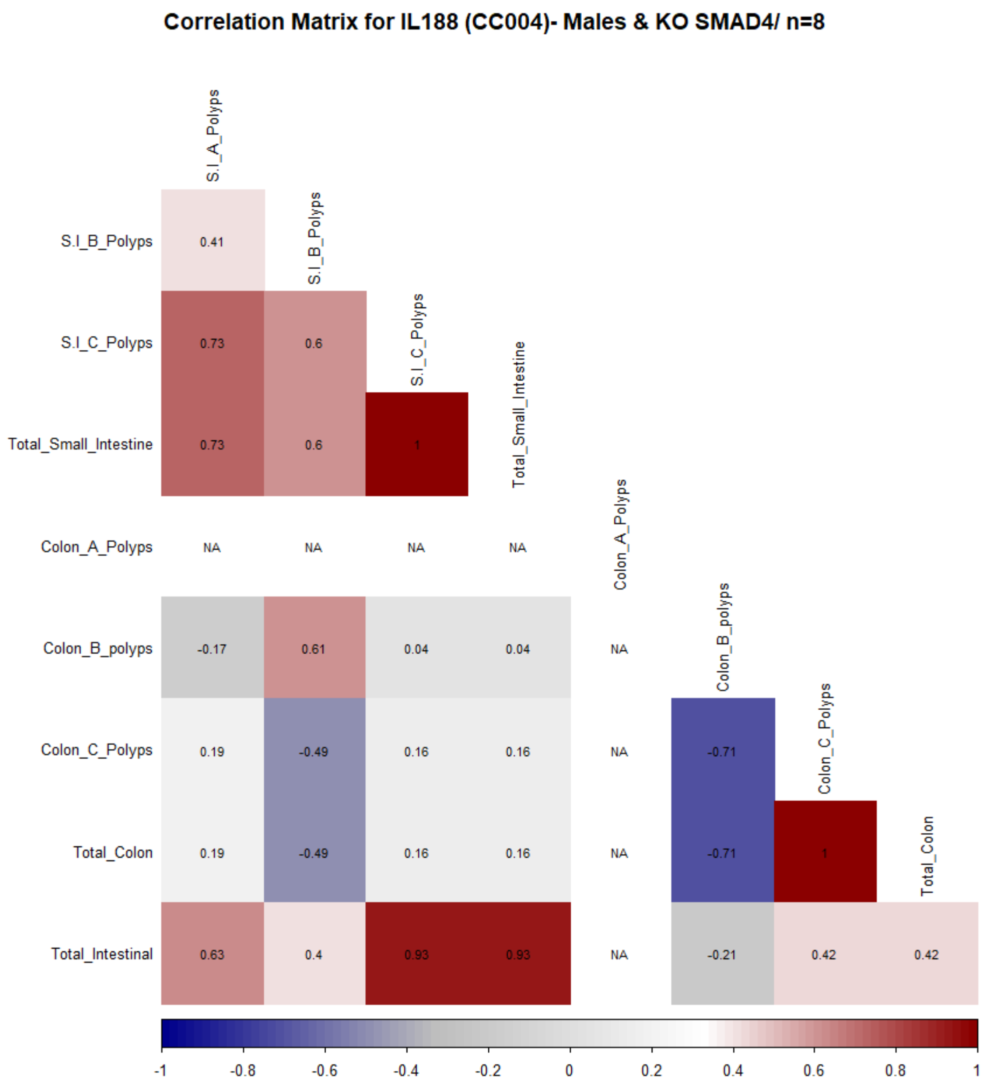

Supplementary Figure S10 Correlation Analysis of Polyp Development Patterns in HETEROZYGOUS KNOCK-OUT male Mice from line CC004.

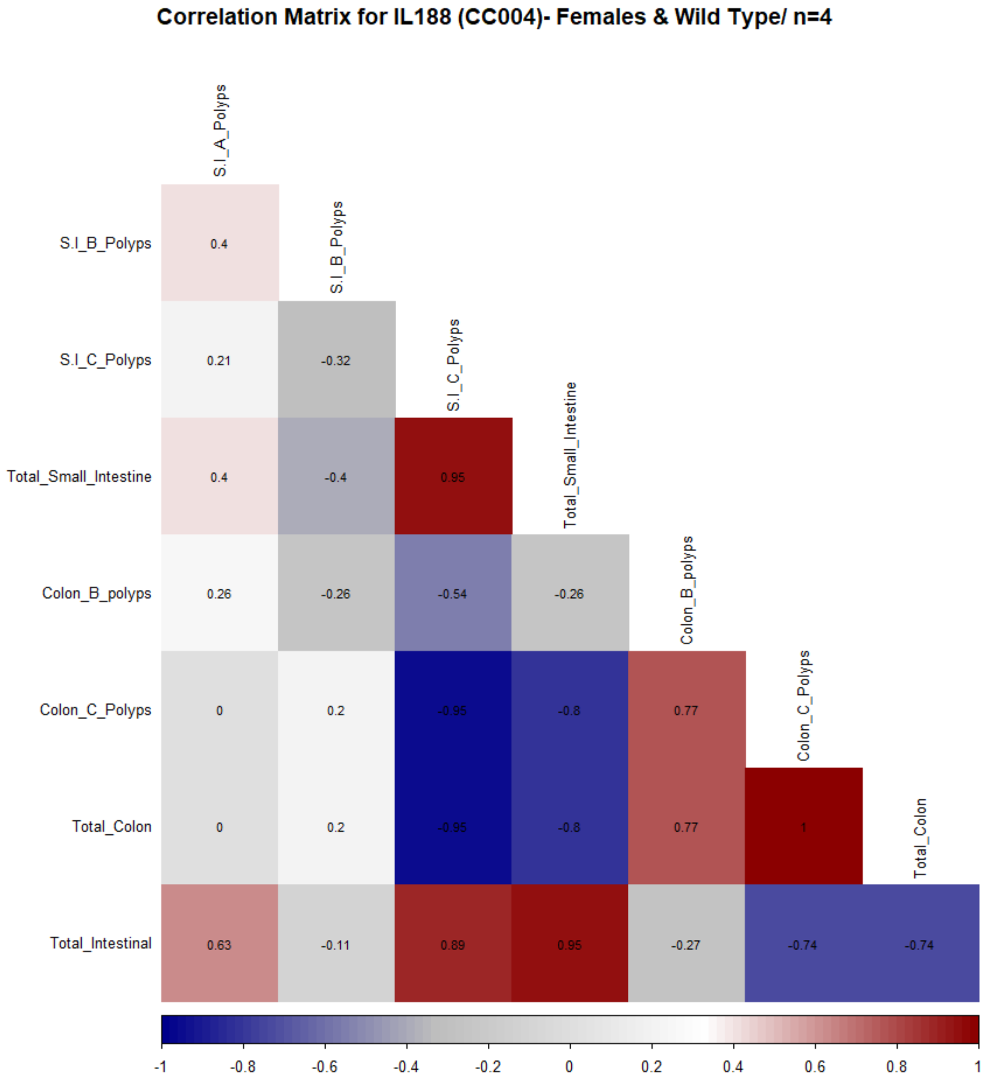

Supplementary Figure S11 Correlation Analysis of Polyp Development Patterns in Wild-Type female Mice from line CC004.

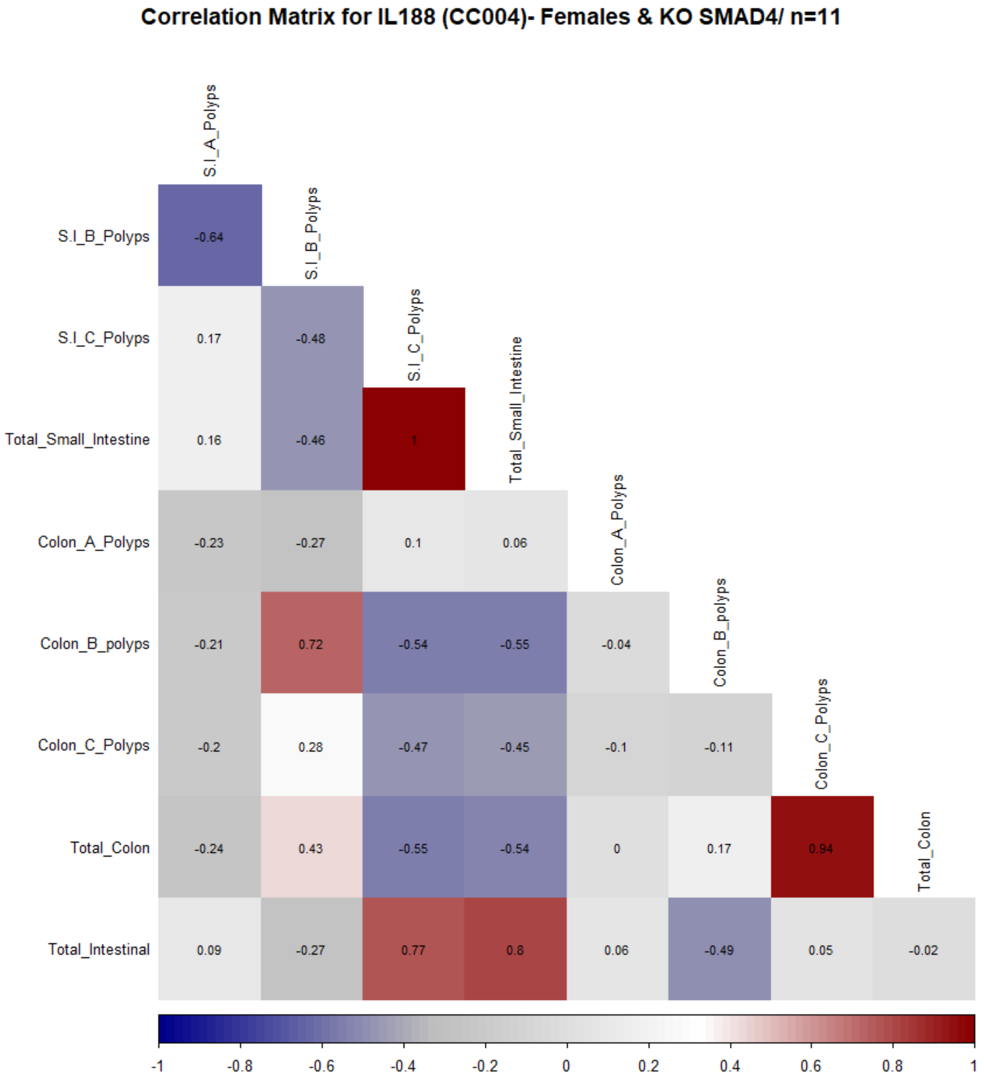

Supplementary Figure S12 Correlation Analysis of Polyp Development Patterns in HETEROZYGOUS KNOCK-OUT female Mice from line CC004.

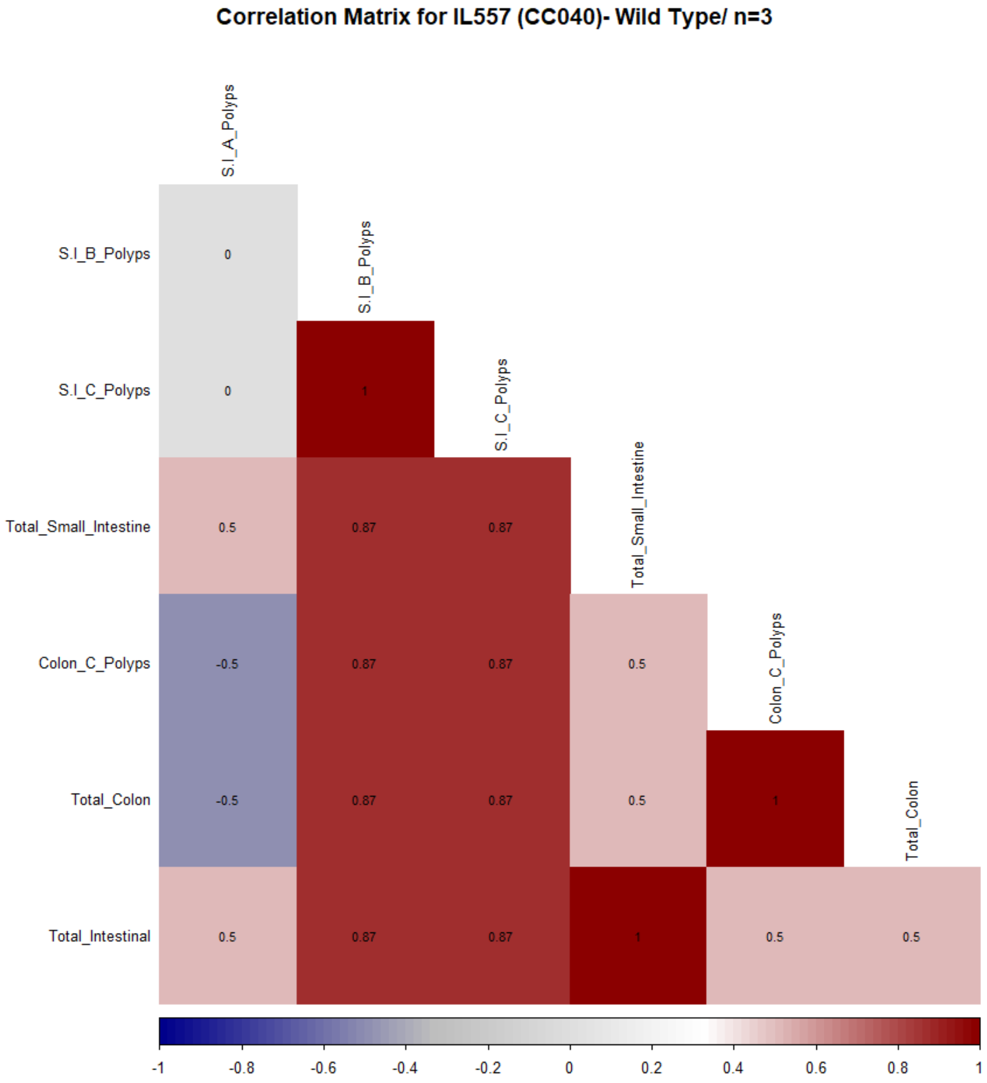

Supplementary Figure S13 Correlation Analysis of Polyp Development Patterns in Wild-Type Mice from line CC040.

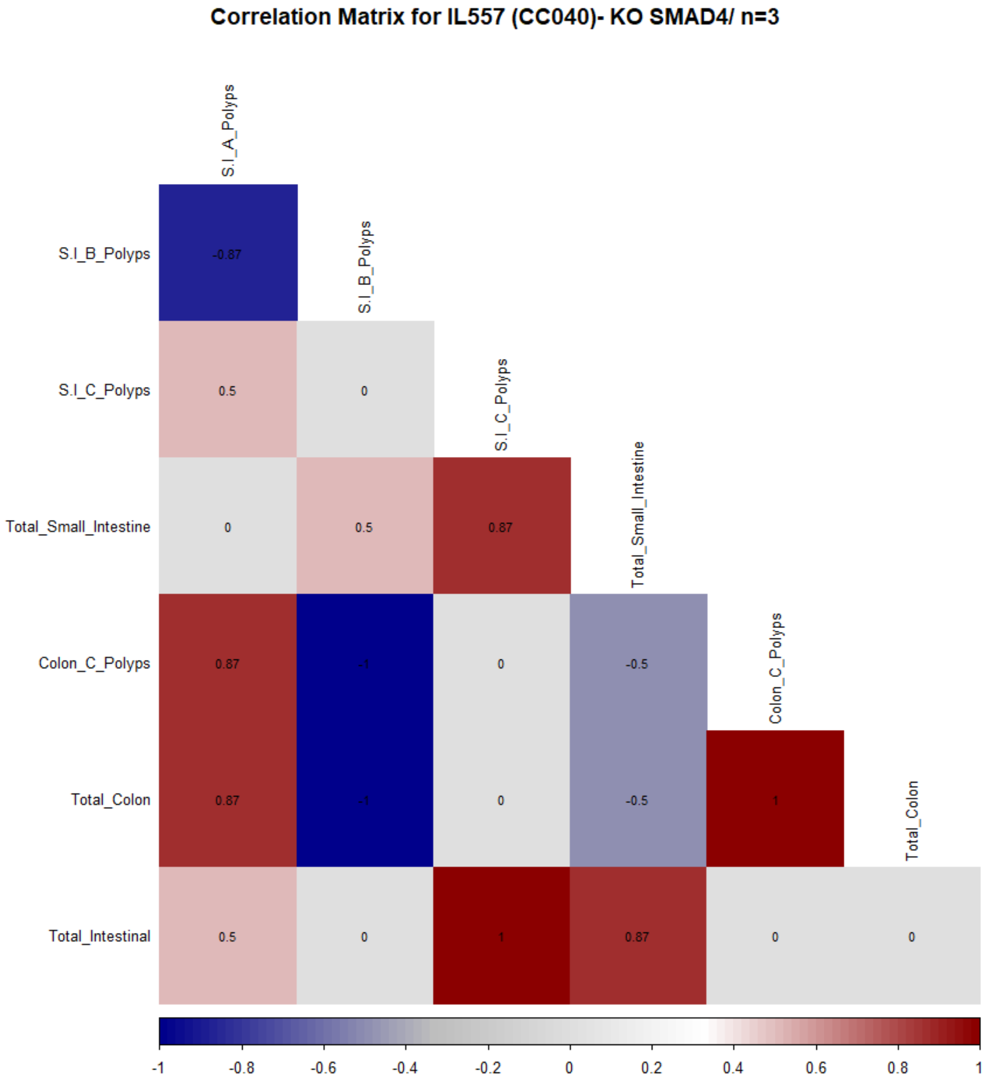

Supplementary Figure S14 Correlation Analysis of Polyp Development Patterns in HETEROZYGOUS KNOCK-OUT Mice from line CC040.

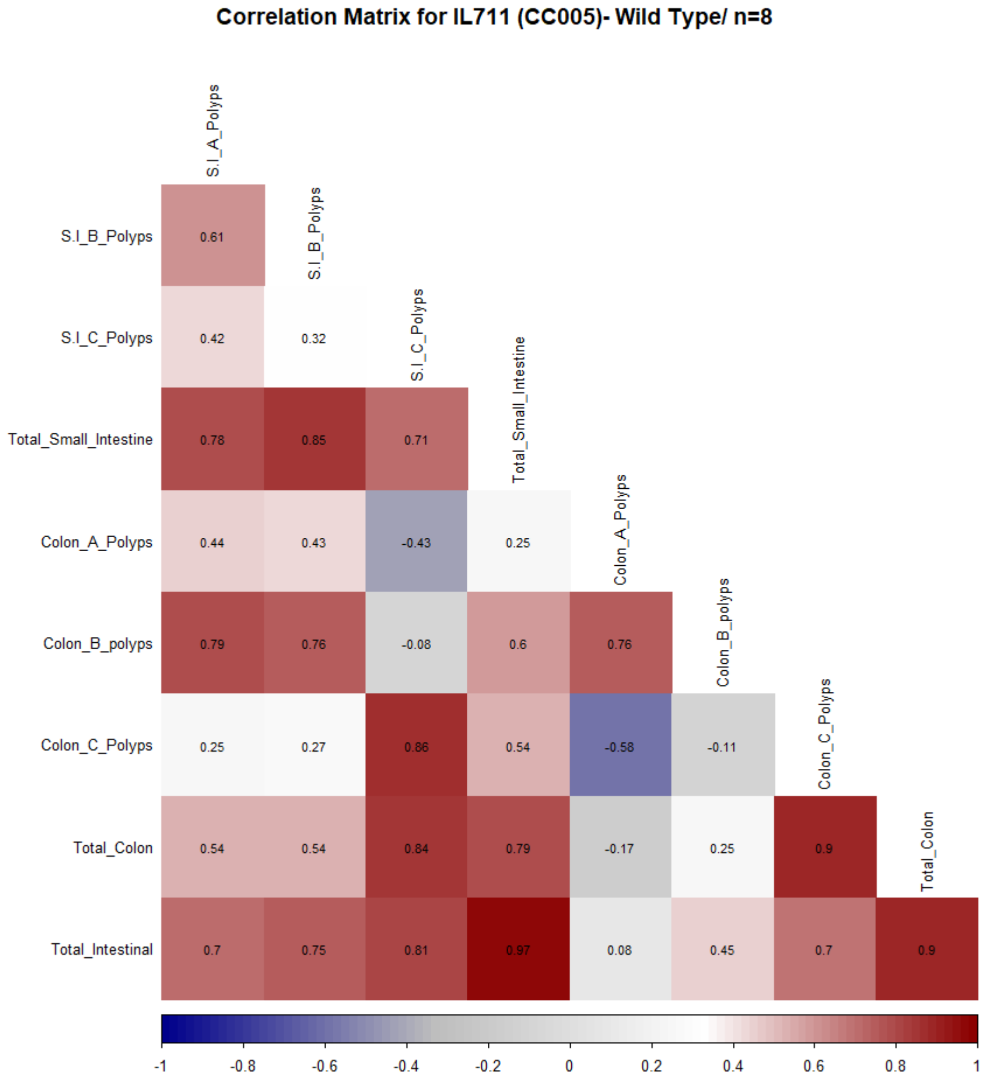

Supplementary Figure S15 Correlation Analysis of Polyp Development Patterns in Wild-Type Mice from line CC005.

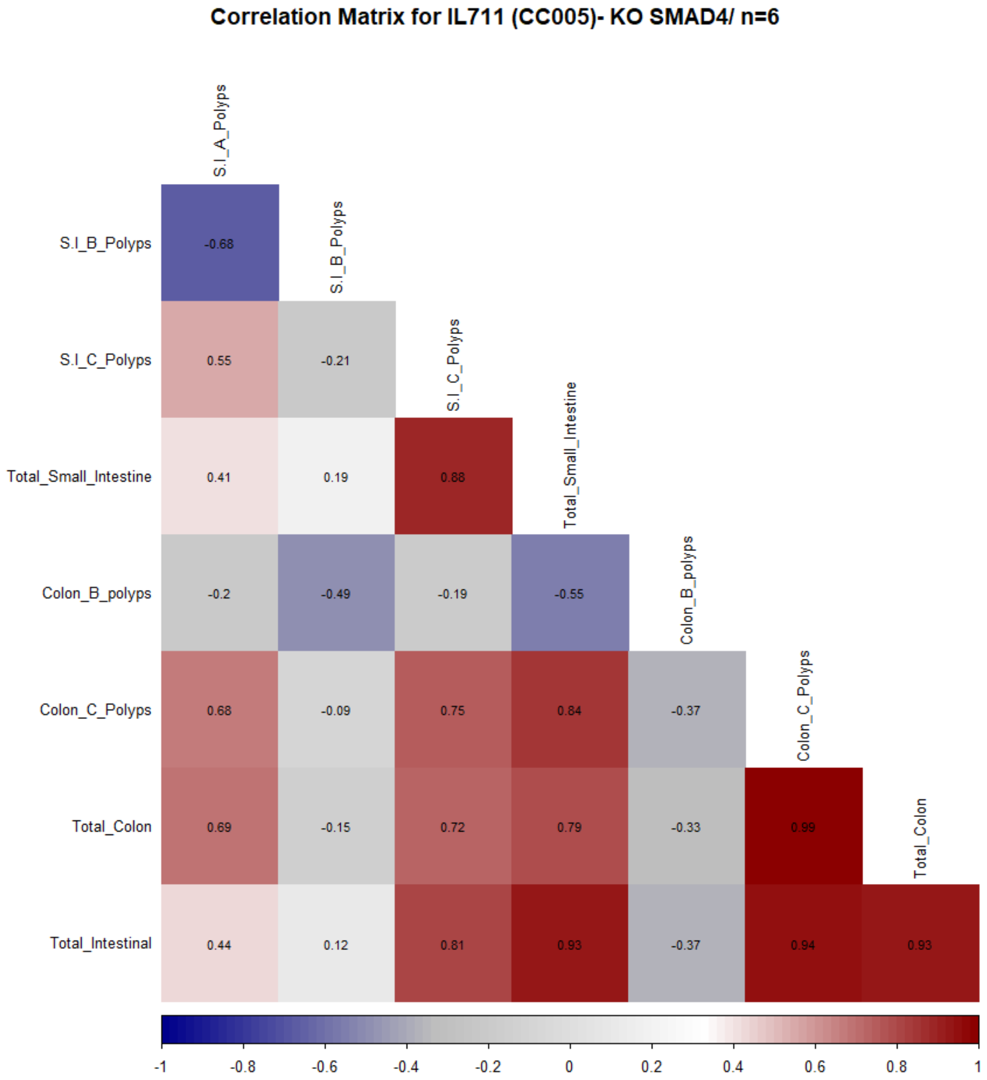

Supplementary Figure S16 Correlation Analysis of Polyp Development Patterns in HETEROZYGOUS KNOCK-OUT Mice from line CC005.

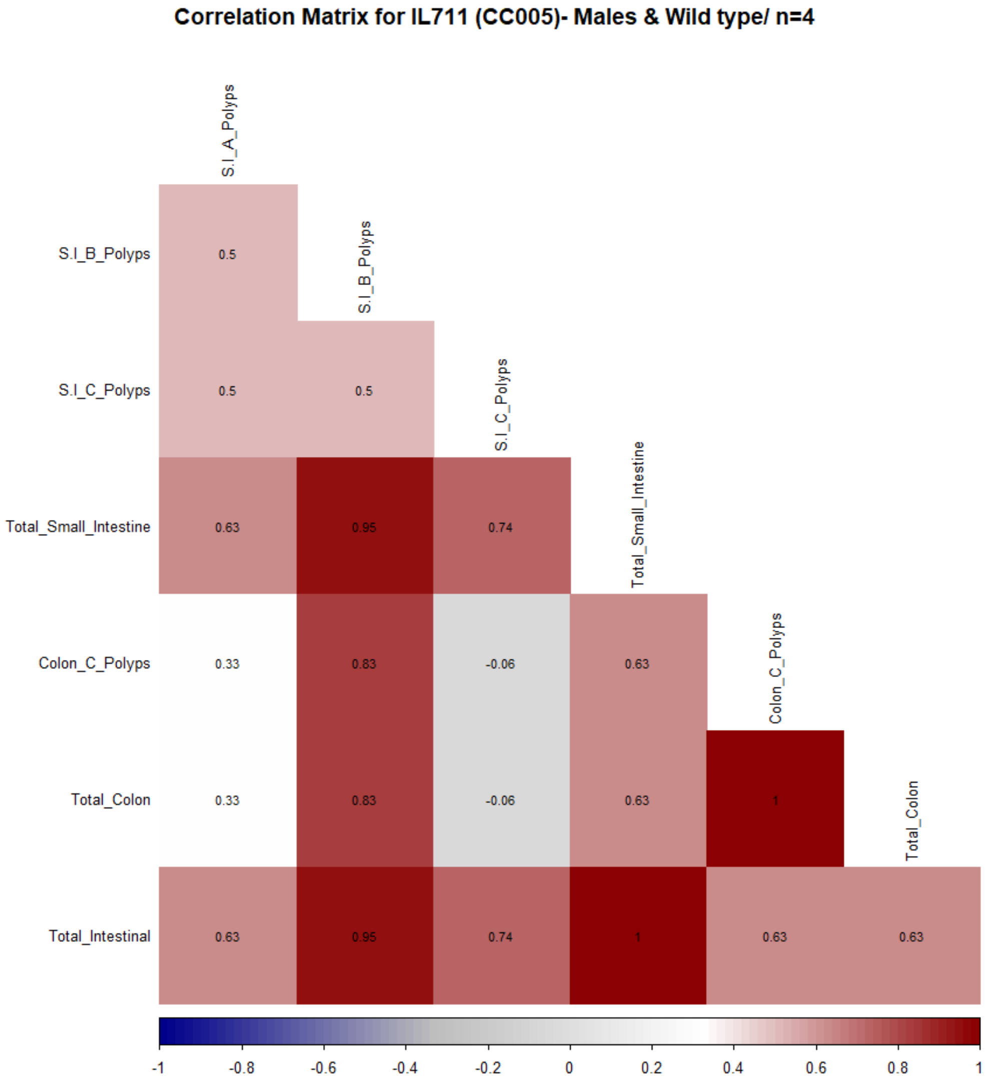

Supplementary Figure S17 Correlation Analysis of Polyp Development Patterns in Wild-Type male Mice from line CC005.

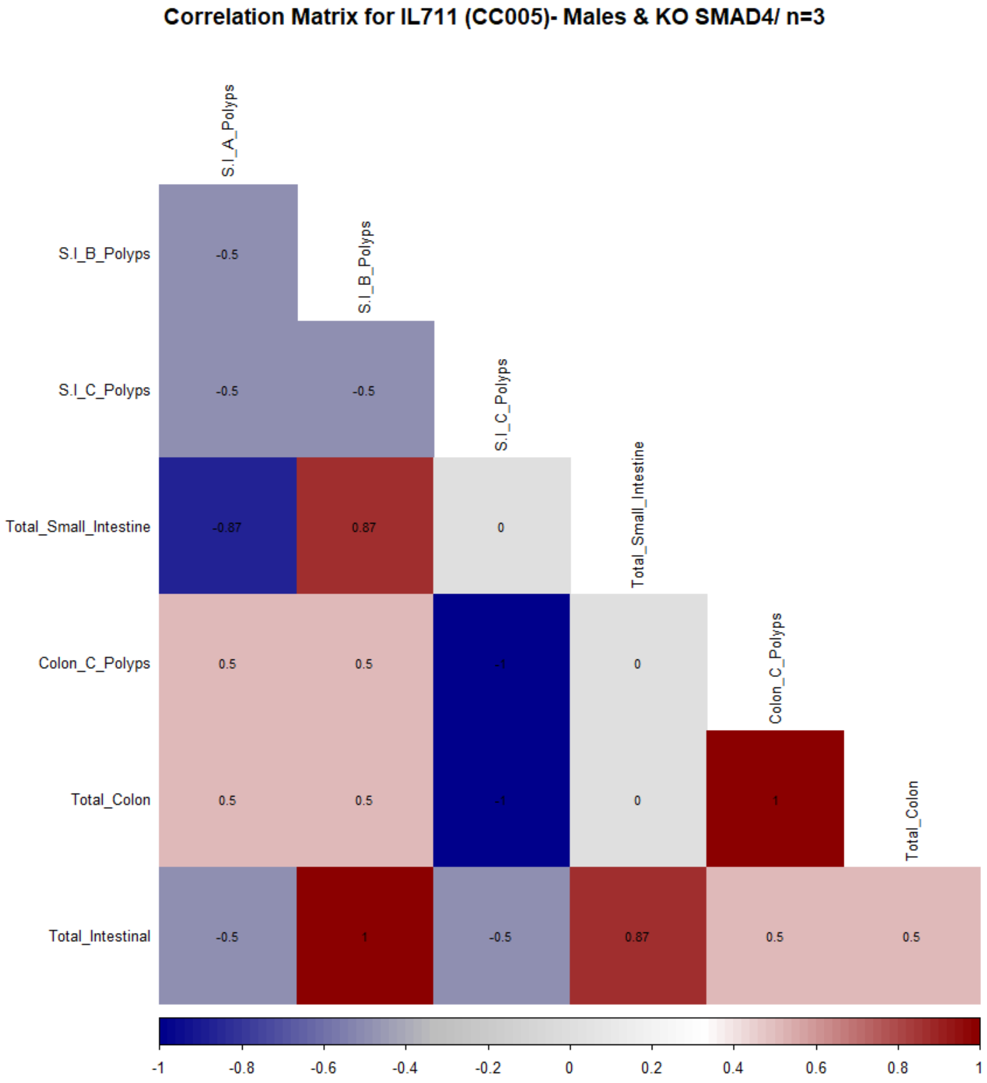

Supplementary Figure S18 Correlation Analysis of Polyp Development Patterns in HETEROZYGOUS KNOCK-OUT male Mice from line CC005.

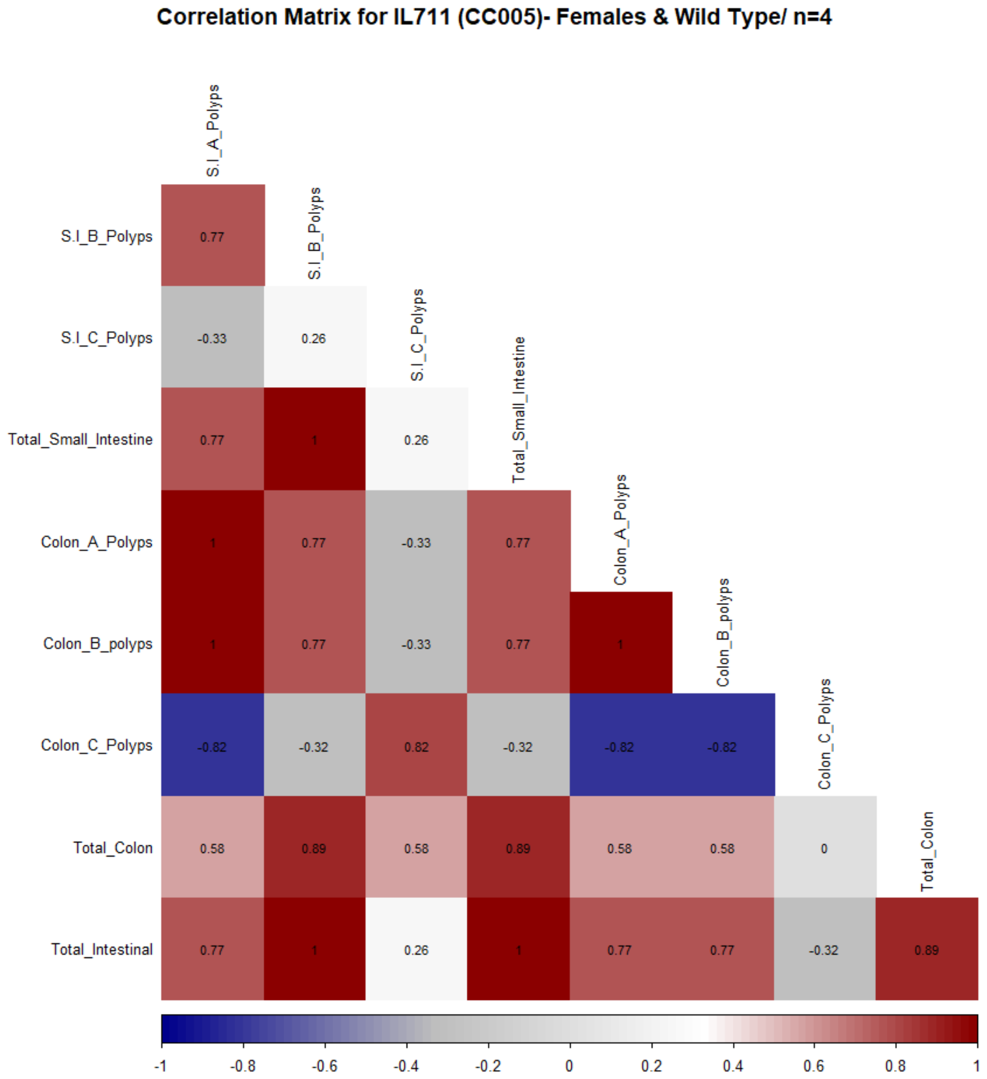

Supplementary Figure S19 Correlation Analysis of Polyp Development Patterns in Wild-Type female Mice from line CC005.

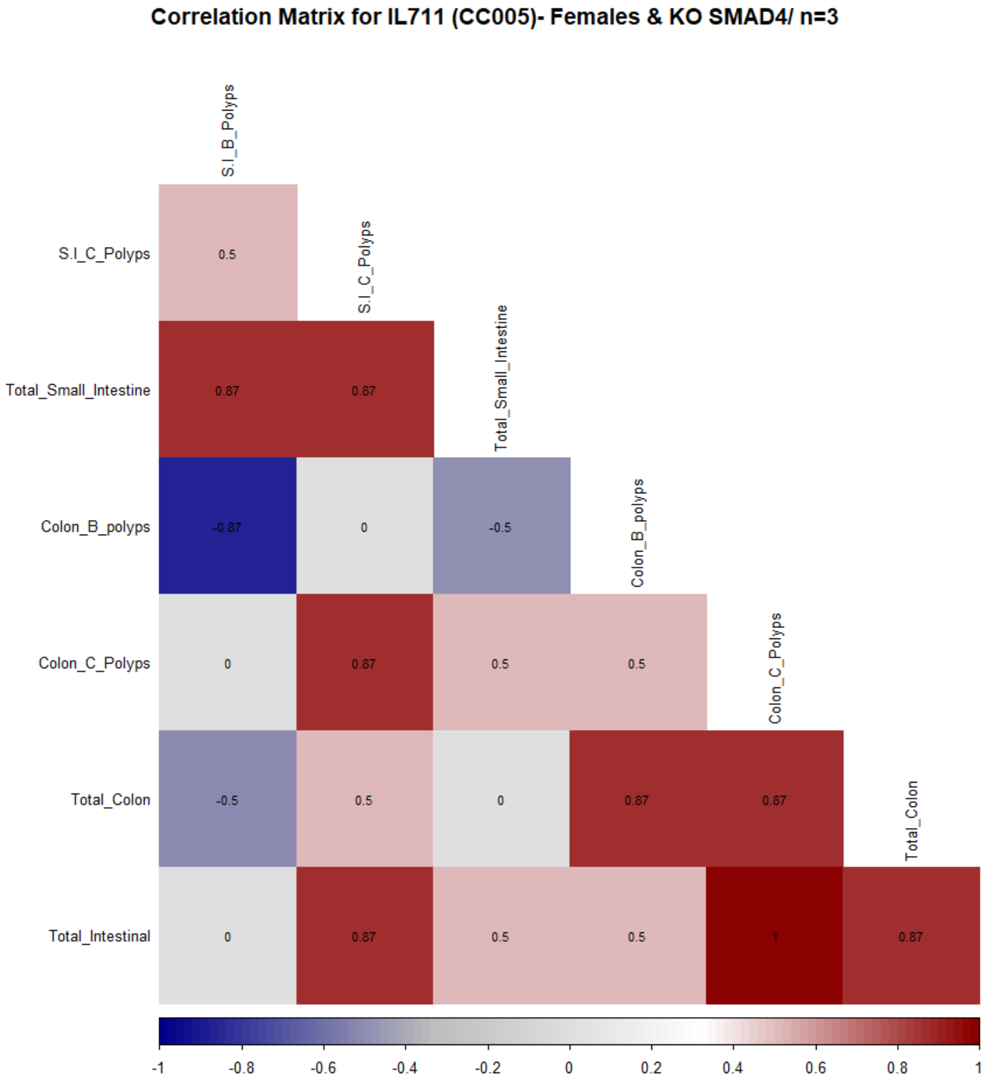

Supplementary Figure S20 Correlation Analysis of Polyp Development Patterns in HETEROZYGOUS KNOCK-OUT female Mice from line CC005.

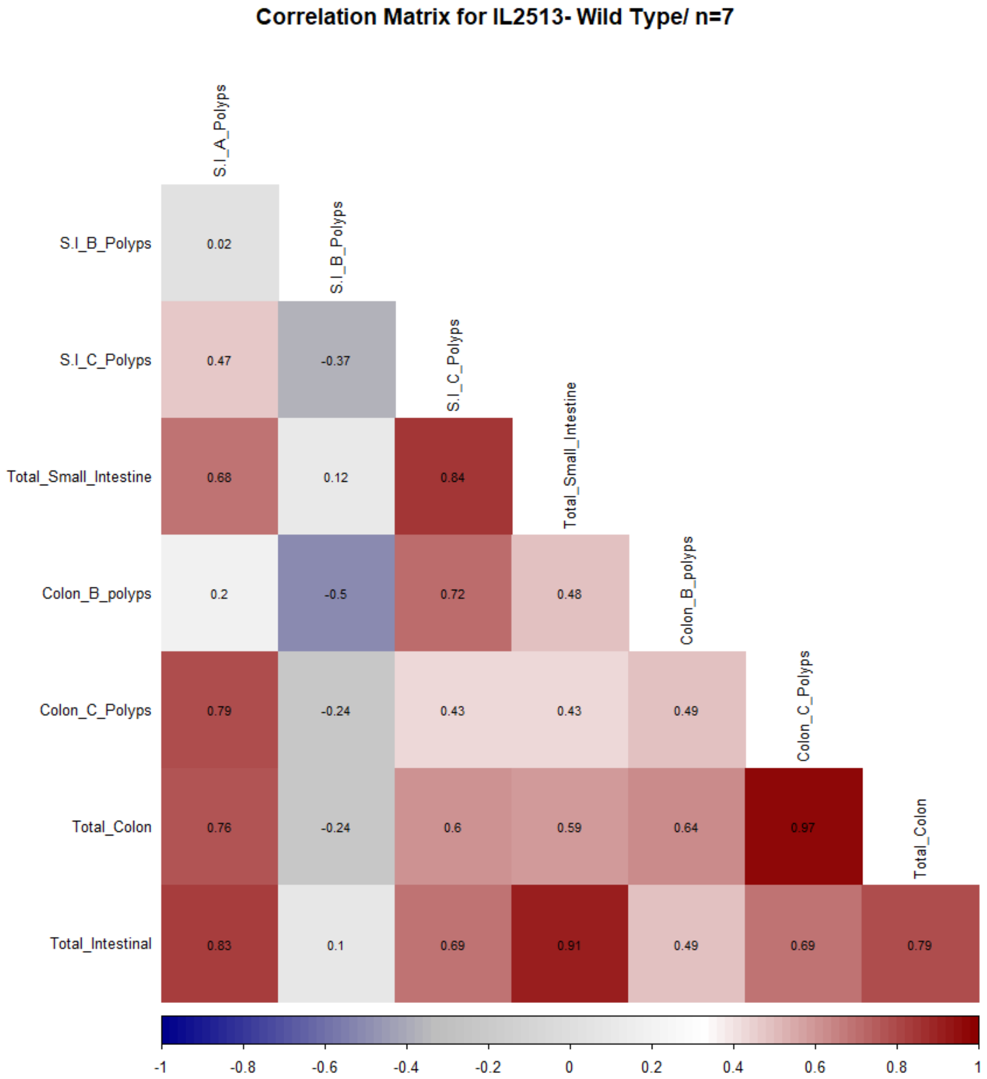

Supplementary Figure S21 Correlation Analysis of Polyp Development Patterns in Wild-Type Mice from line CC019.

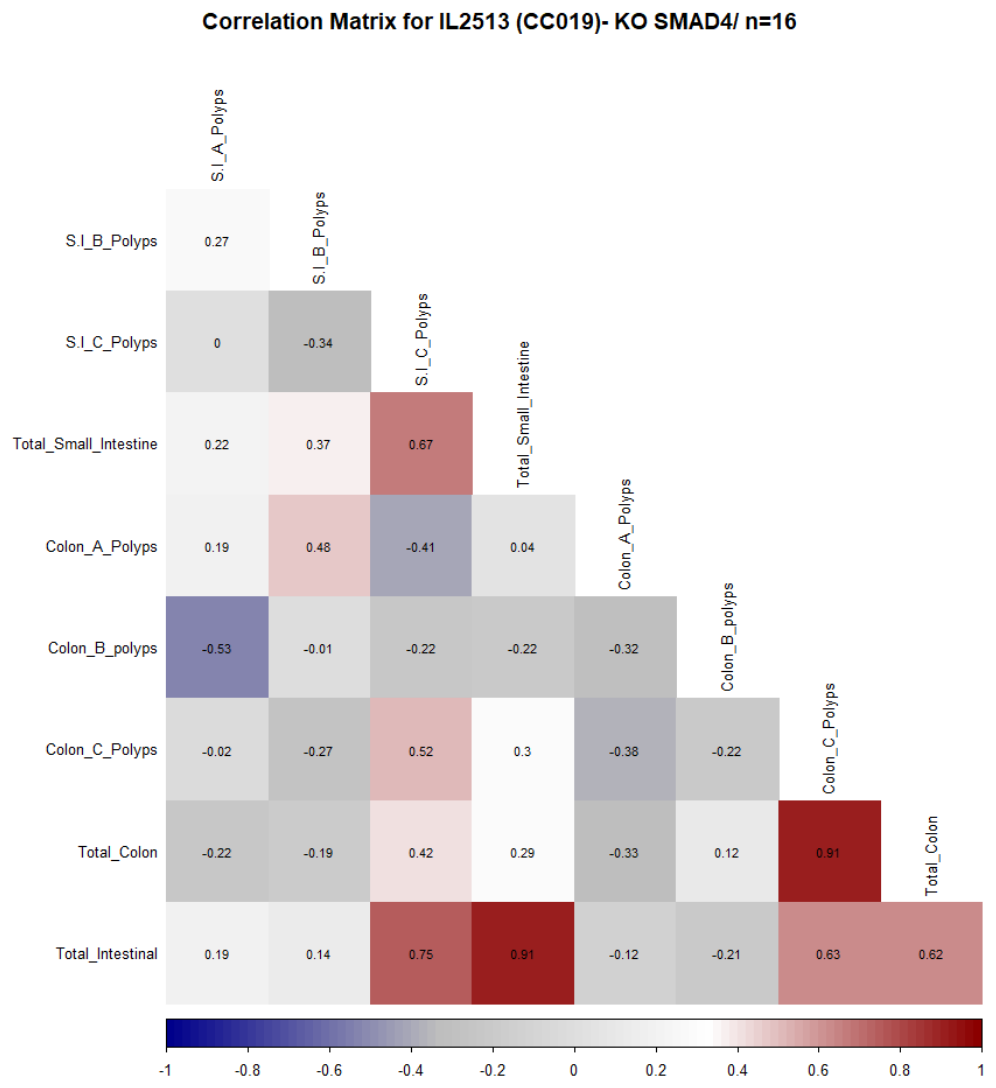

Supplementary Figure S22 Correlation Analysis of Polyp Development Patterns in HETEROZYGOUS KNOCK-OUT Mice from line CC019.

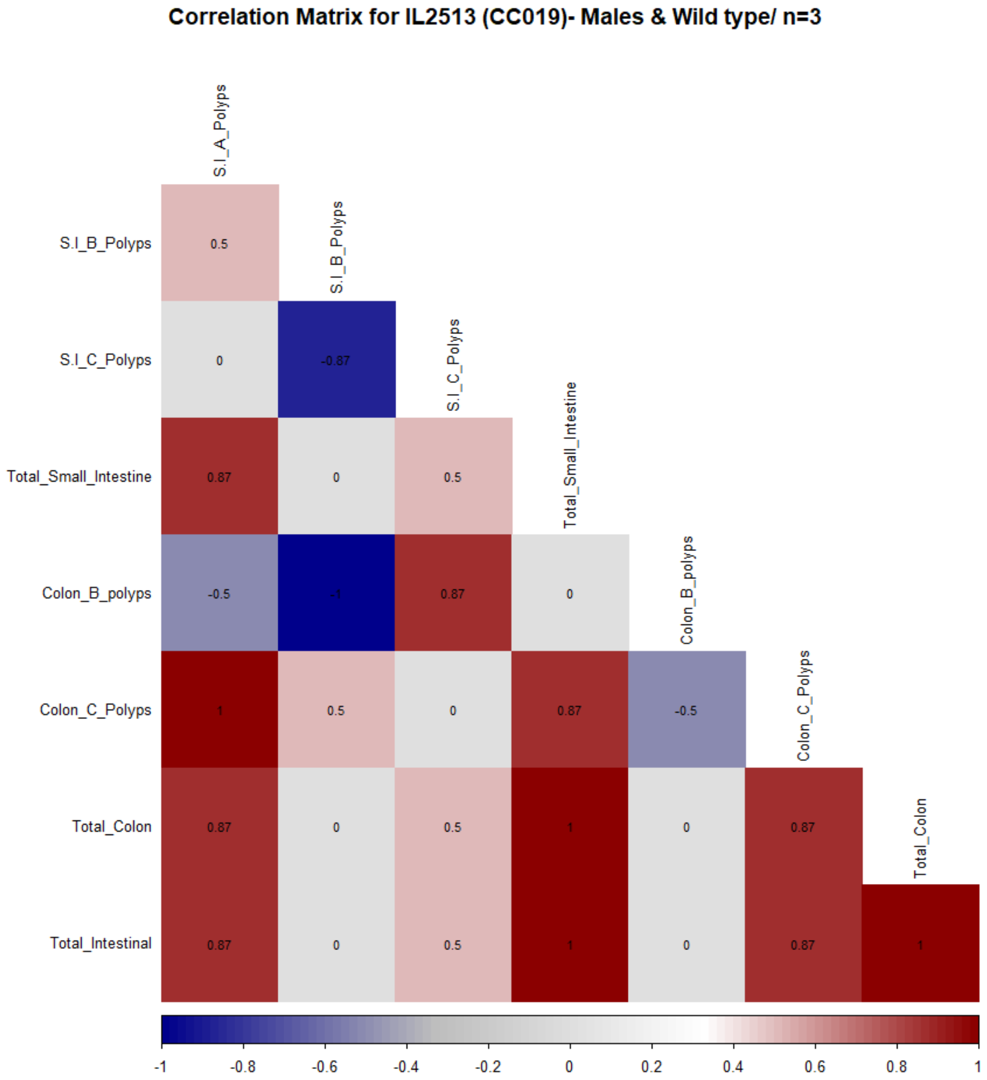

Supplementary Figure S23 Correlation Analysis of Polyp Development Patterns in Wild-Type male Mice from line CC019.

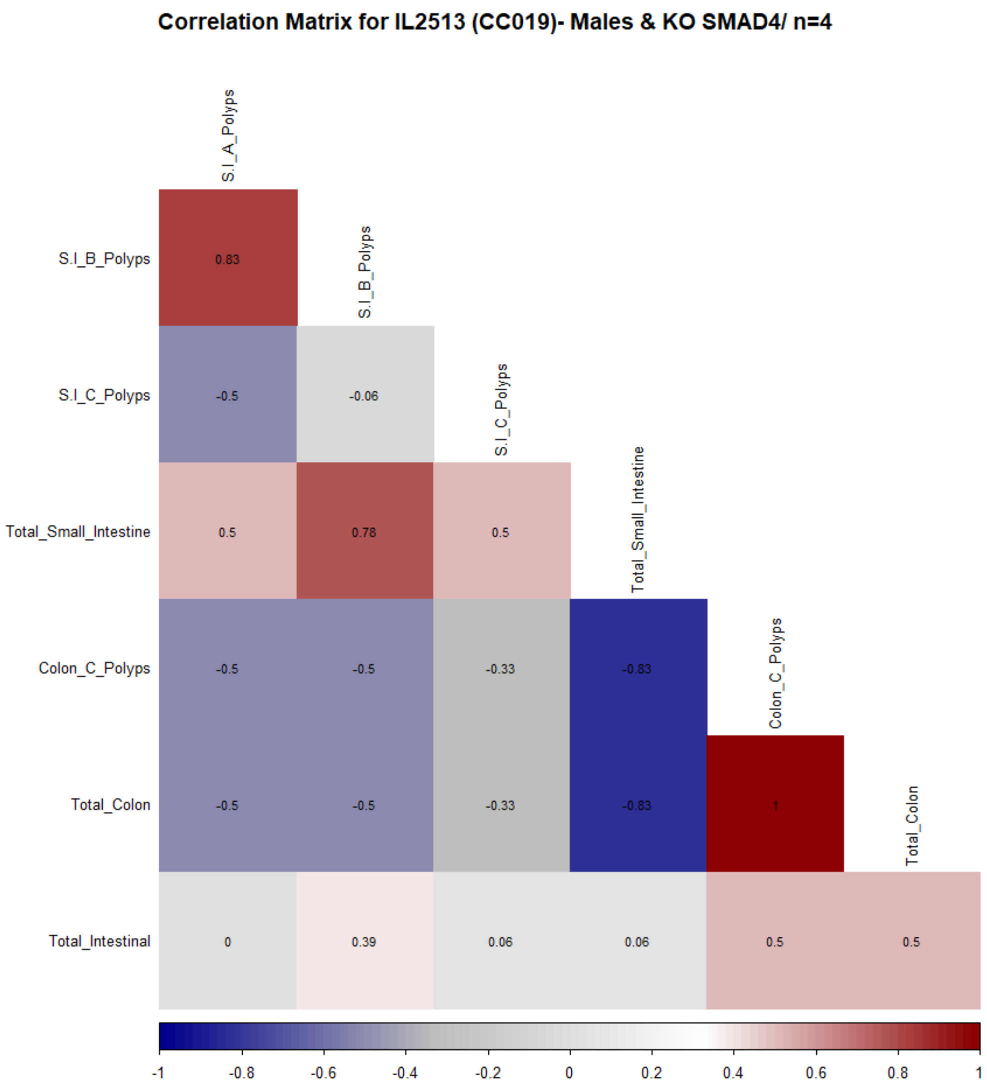

Supplementary Figure S24 Correlation Analysis of Polyp Development Patterns in HETEROZYGOUS KNOCK-OUT male Mice from line CC019.

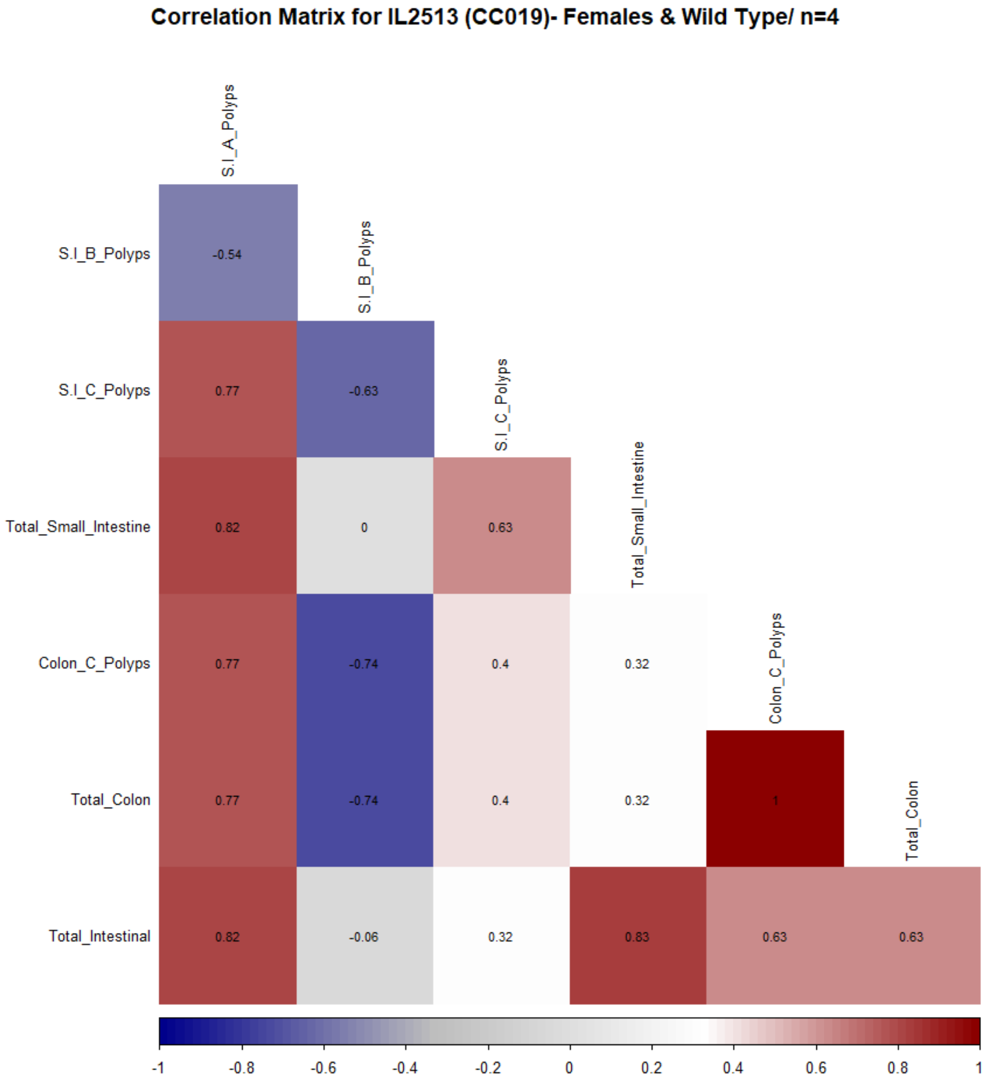

Supplementary Figure S25 Correlation Analysis of Polyp Development Patterns in Wild-Type female Mice from line CC019.

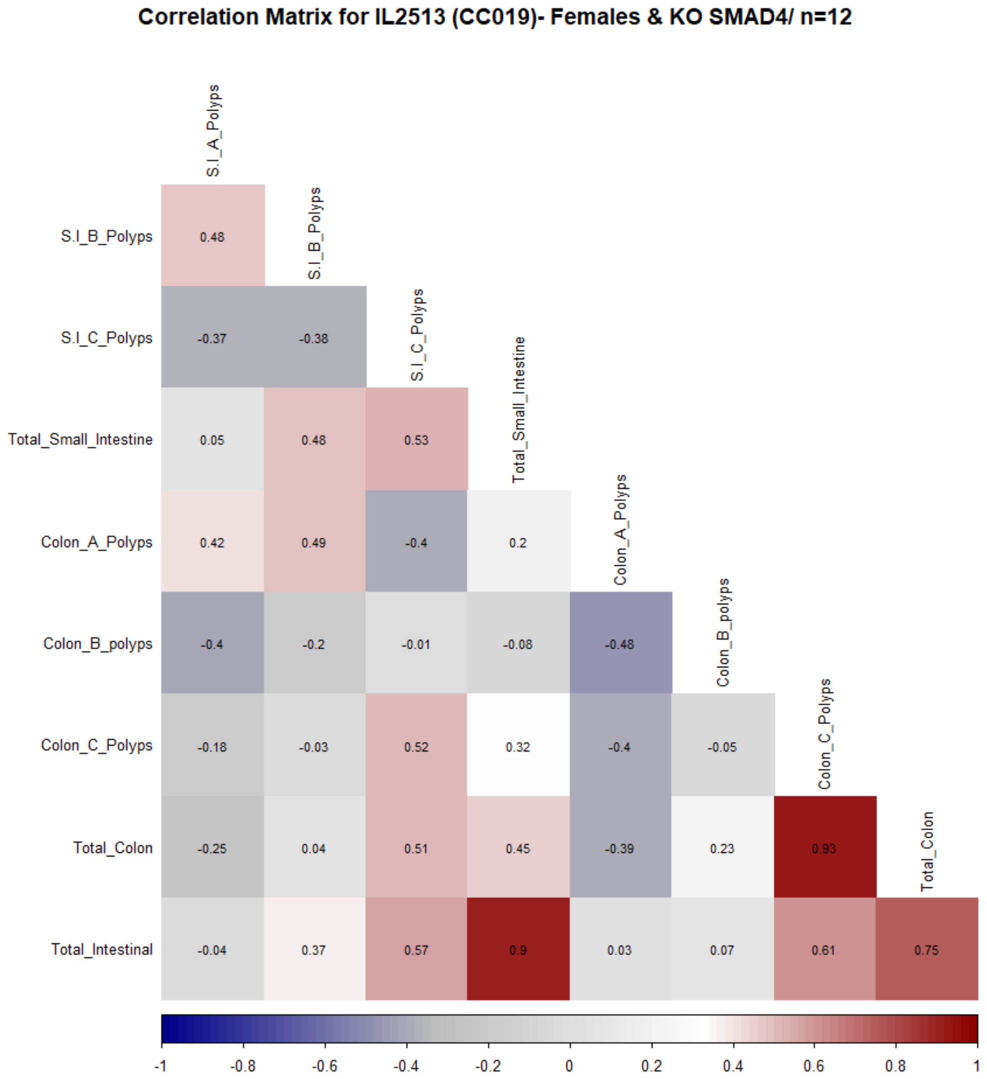

Supplementary Figure S26 Correlation Analysis of Polyp Development Patterns in HETEROZYGOUS KNOCK-OUT female Mice from line CC019.

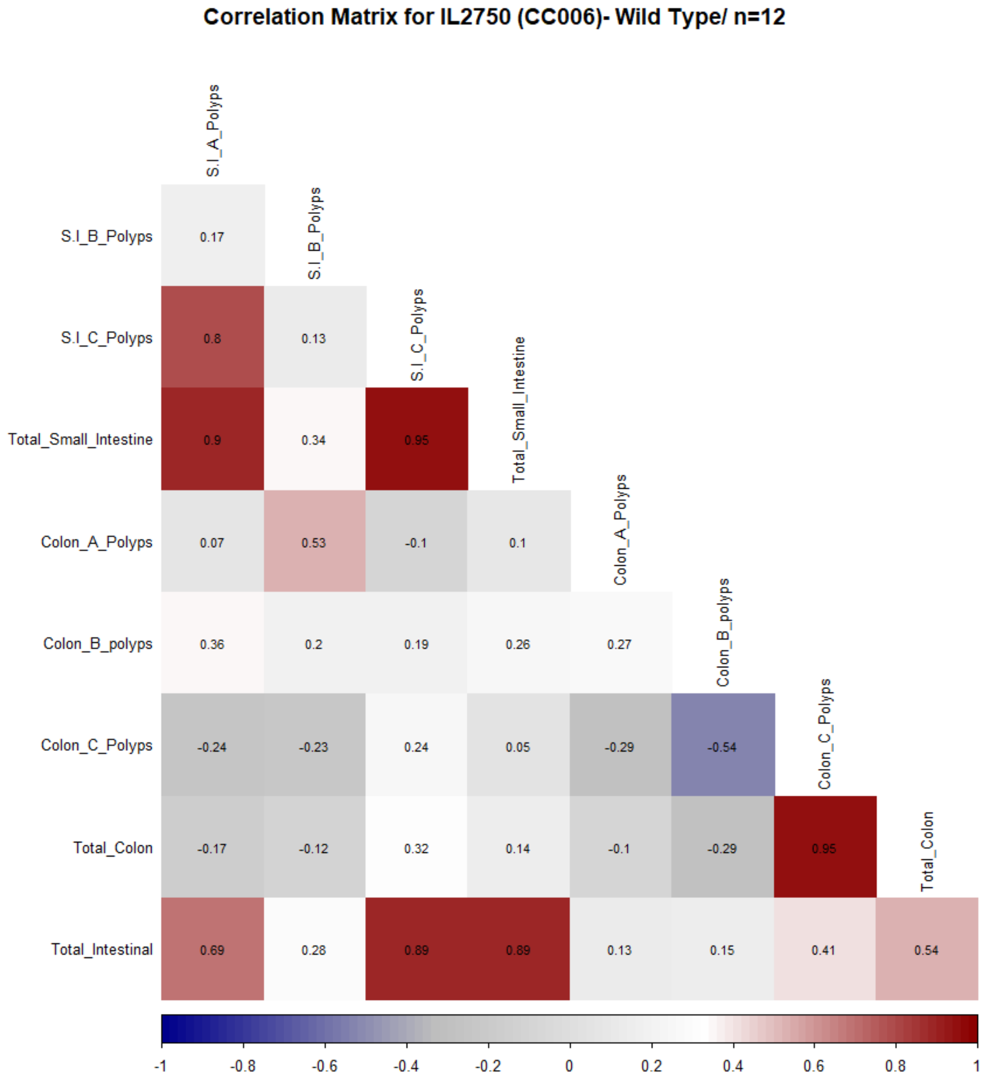

Supplementary Figure S27: Correlation Analysis of Polyp Development Patterns in Wild-Type Mice from line CC006.

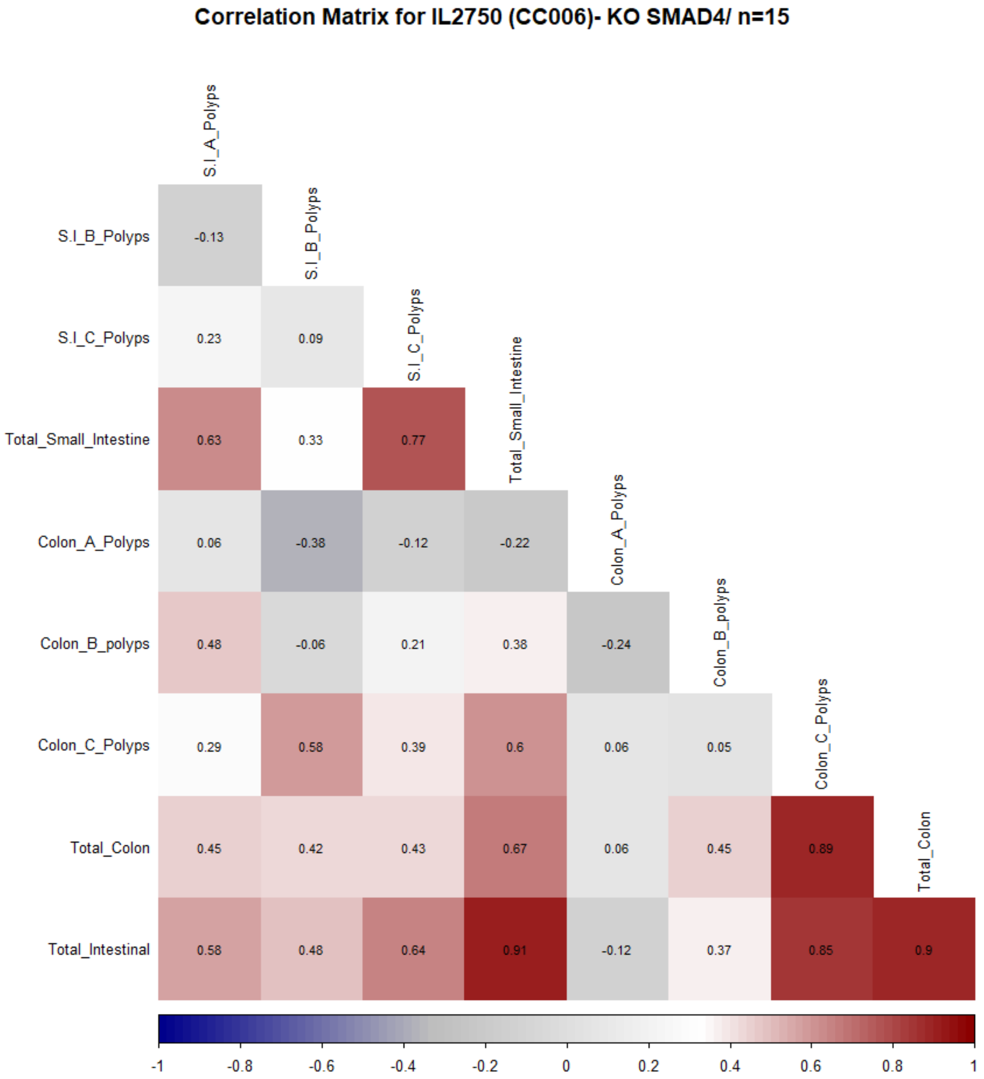

Supplementary Figure S28 Correlation Analysis of Polyp Development Patterns in HETEROZYGOUS KNOCK-OUT Mice from line CC006.

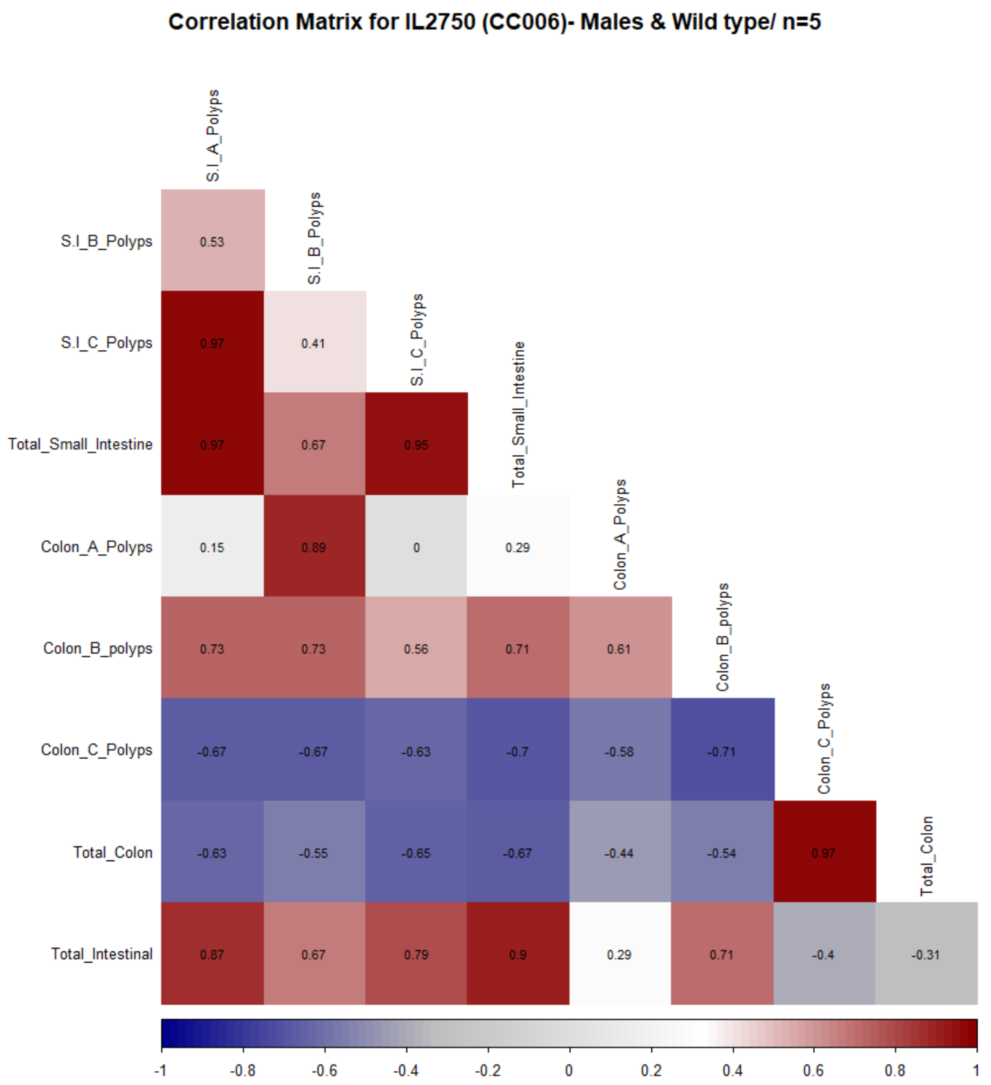

Supplementary Figure S29 Correlation Analysis of Polyp Development Patterns in Wild-Type male Mice from line CC006.

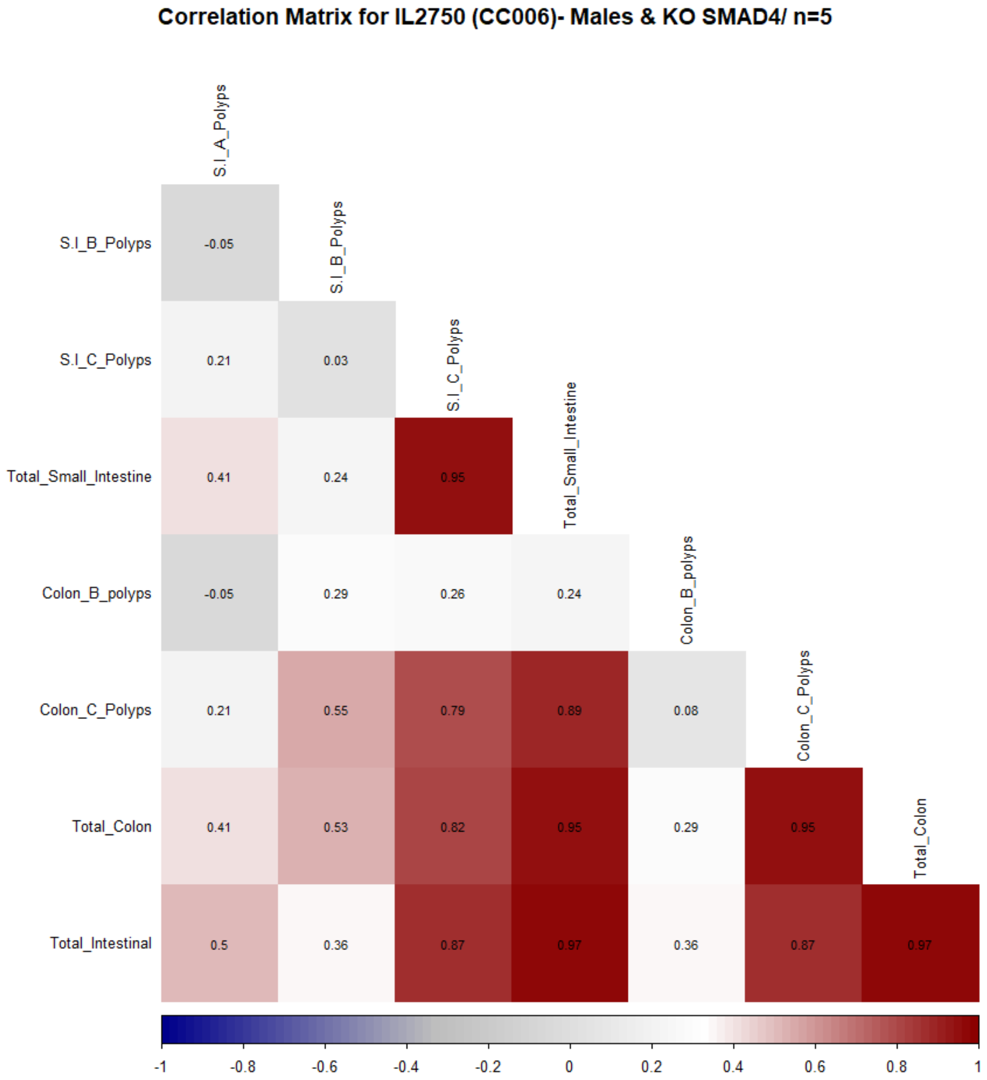

Supplementary Figure S30 Correlation Analysis of Polyp Development Patterns in HETEROZYGOUS KNOCK-OUT male Mice from line CC006.

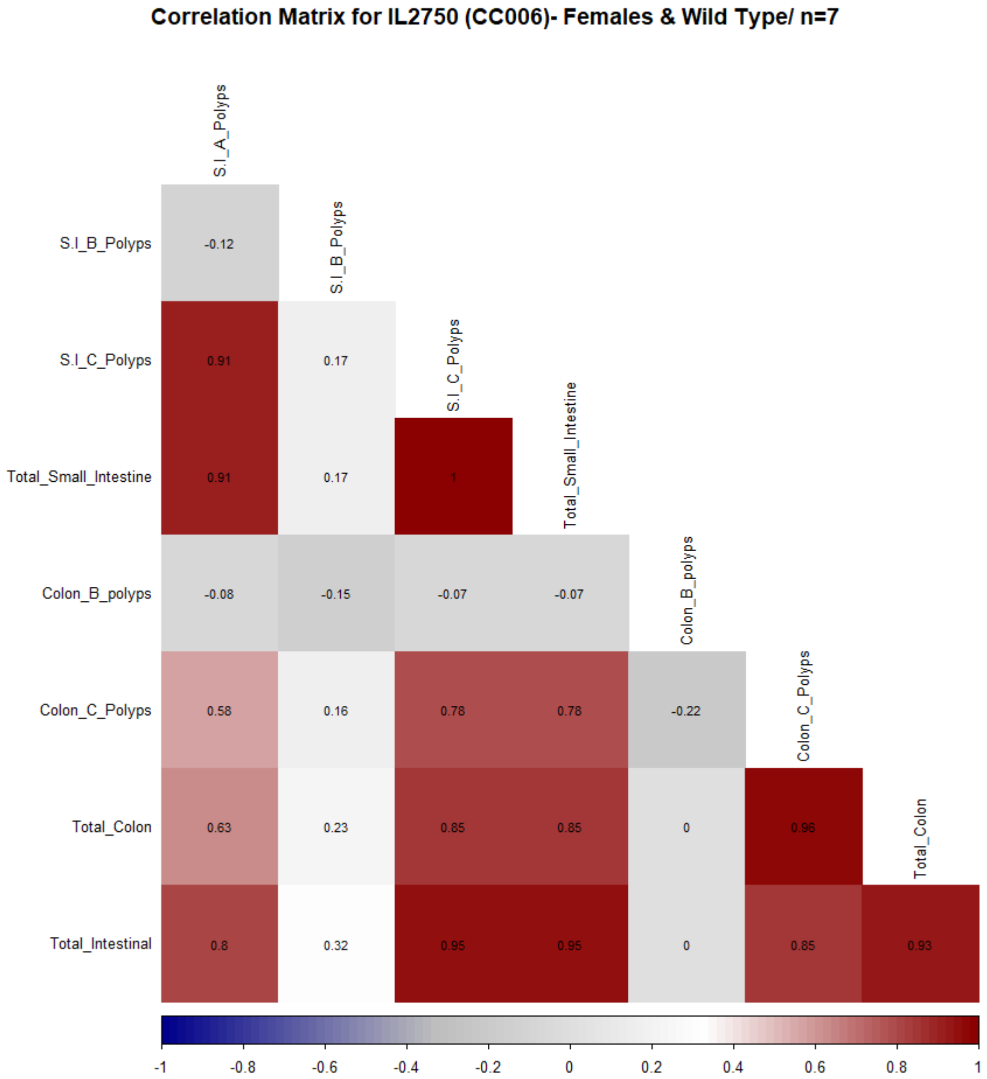

Supplementary Figure S31 Correlation Analysis of Polyp Development Patterns in Wild-Type female Mice from line CC006.

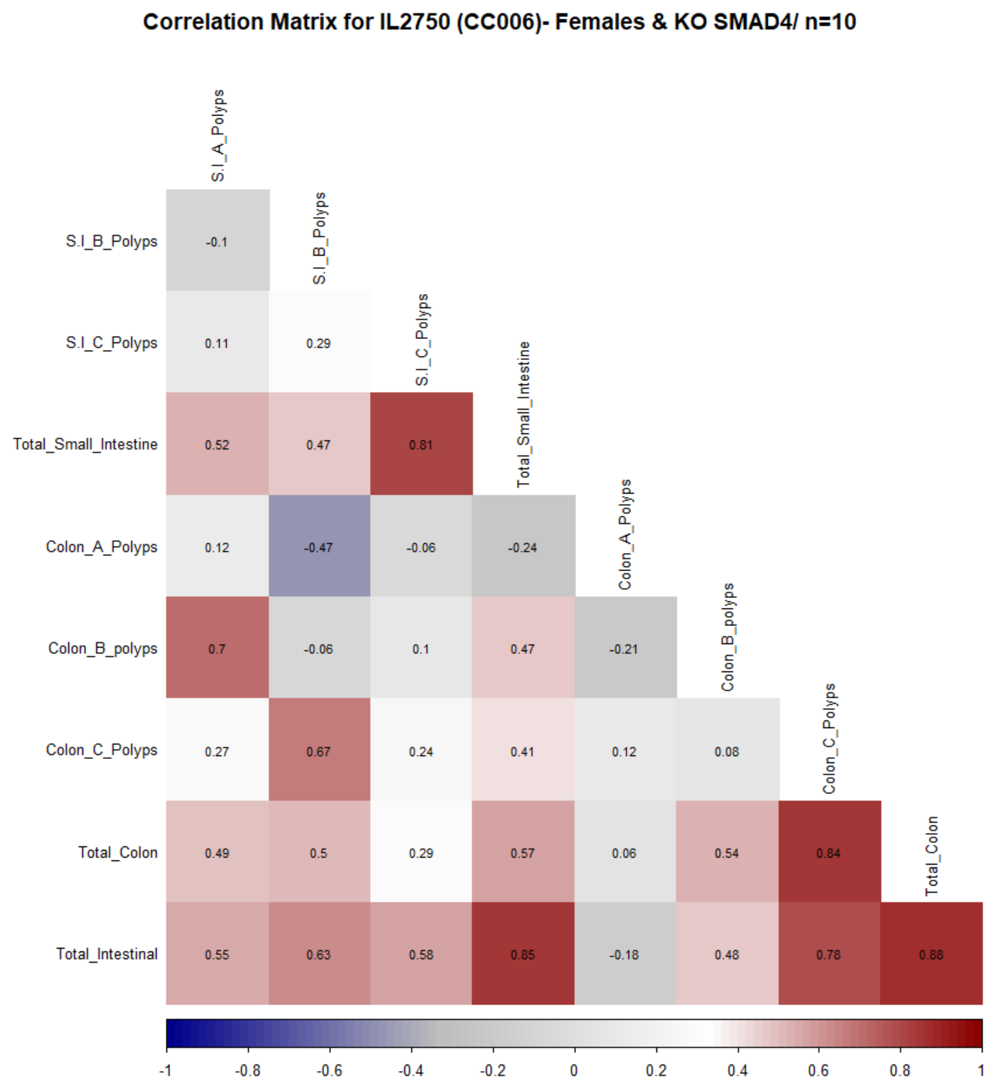

Supplementary Figure S32 Correlation Analysis of Polyp Development Patterns in HETEROZYGOUS KNOCK-OUT female Mice from line CC006.

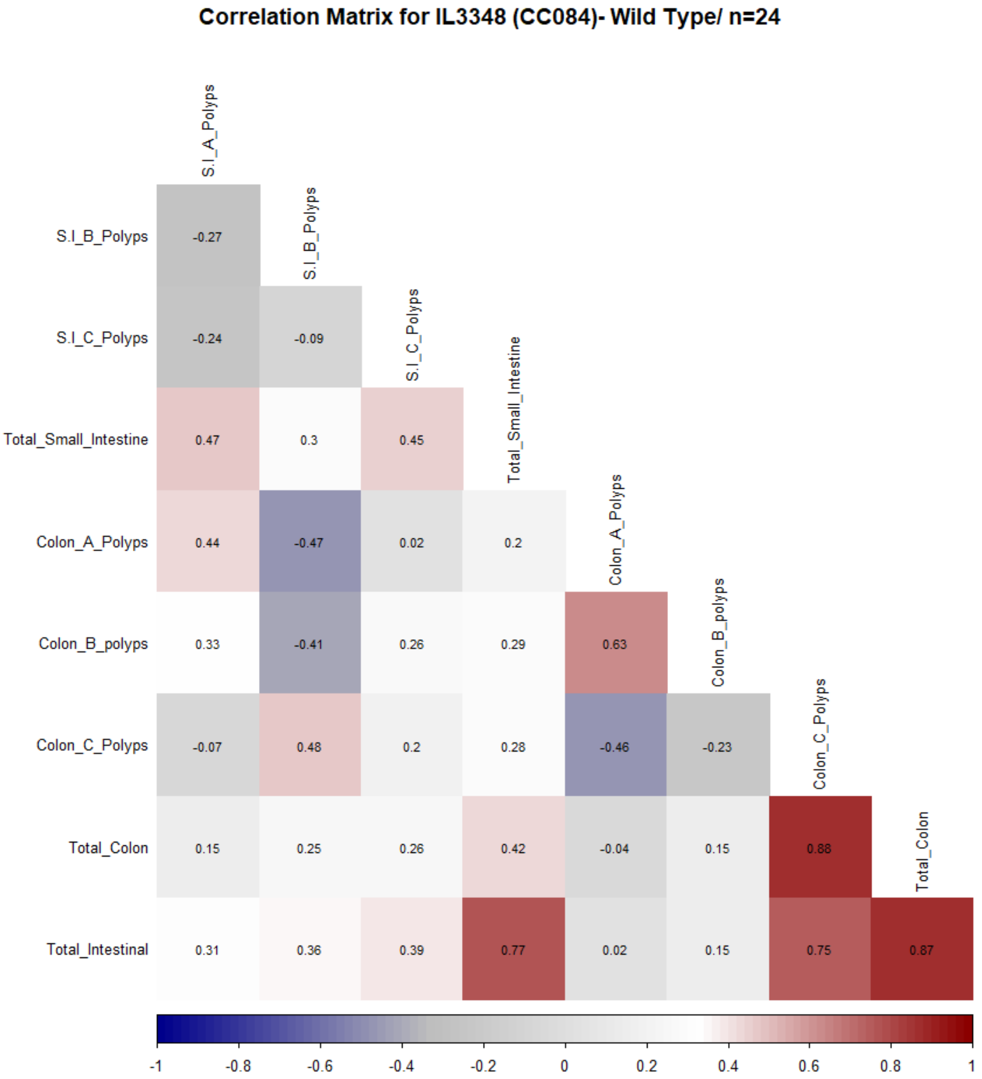

Supplementary Figure S33 Correlation Analysis of Polyp Development Patterns in Wild-Type Mice from line CC084.

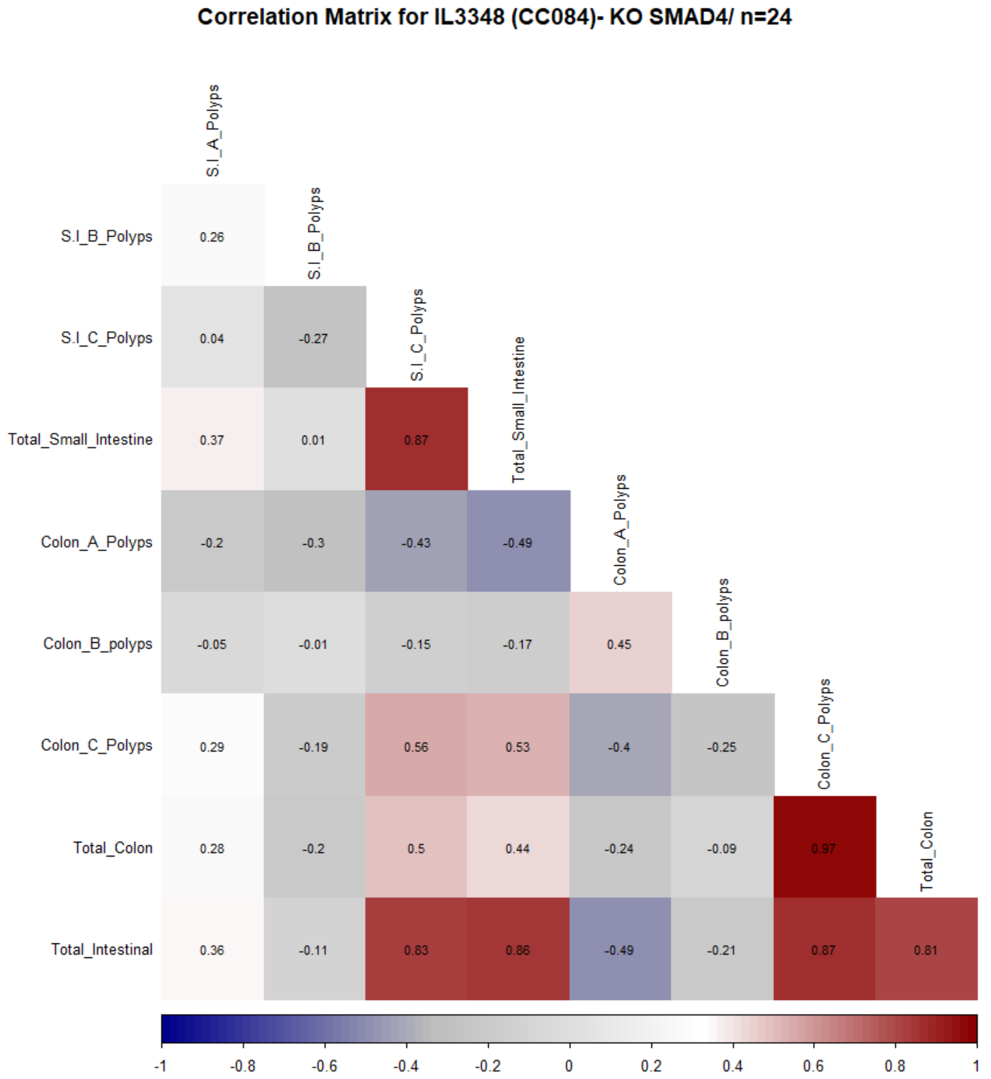

Supplementary Figure S34 Correlation Analysis of Polyp Development Patterns in HETEROZYGOUS KNOCK-OUT Mice from line CC084.

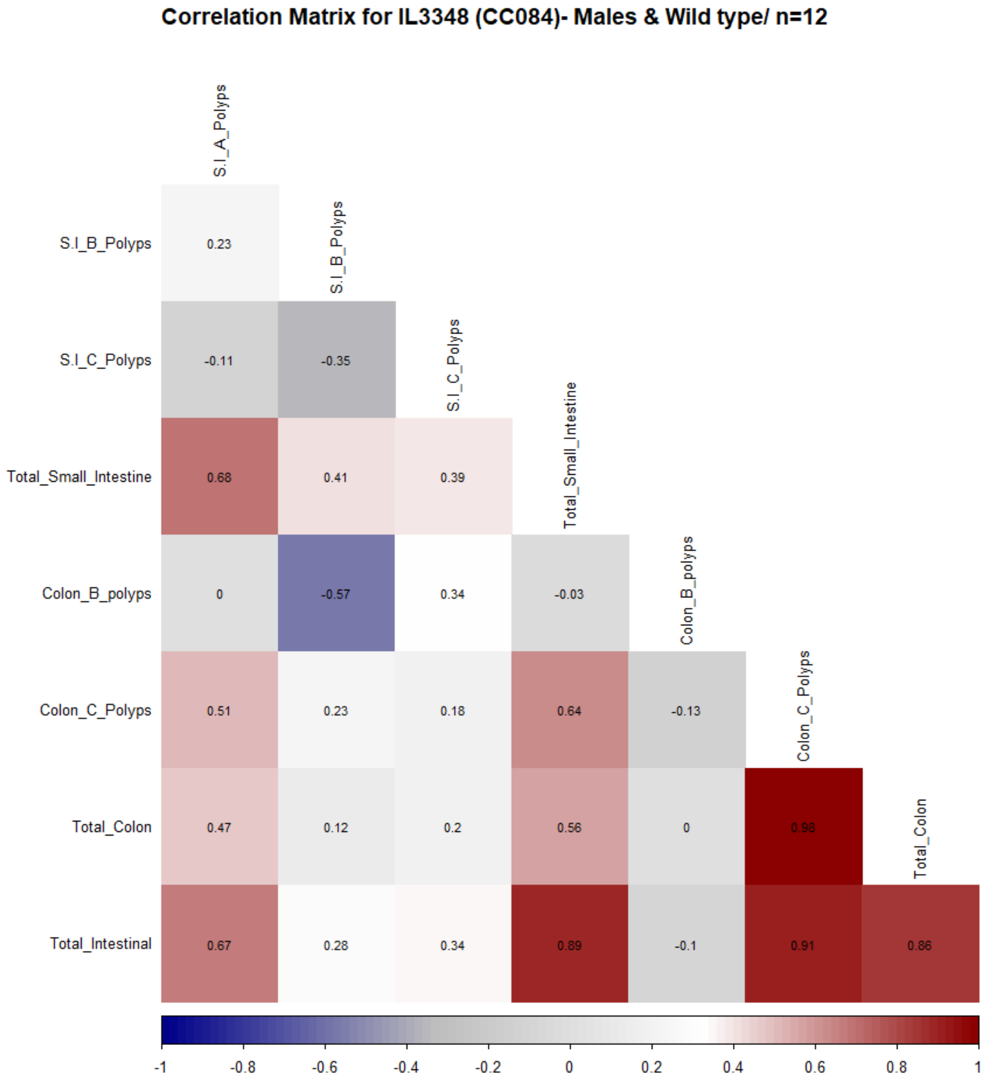

Supplementary Figure S35 Correlation Analysis of Polyp Development Patterns in Wild-Type male Mice from line CC084.

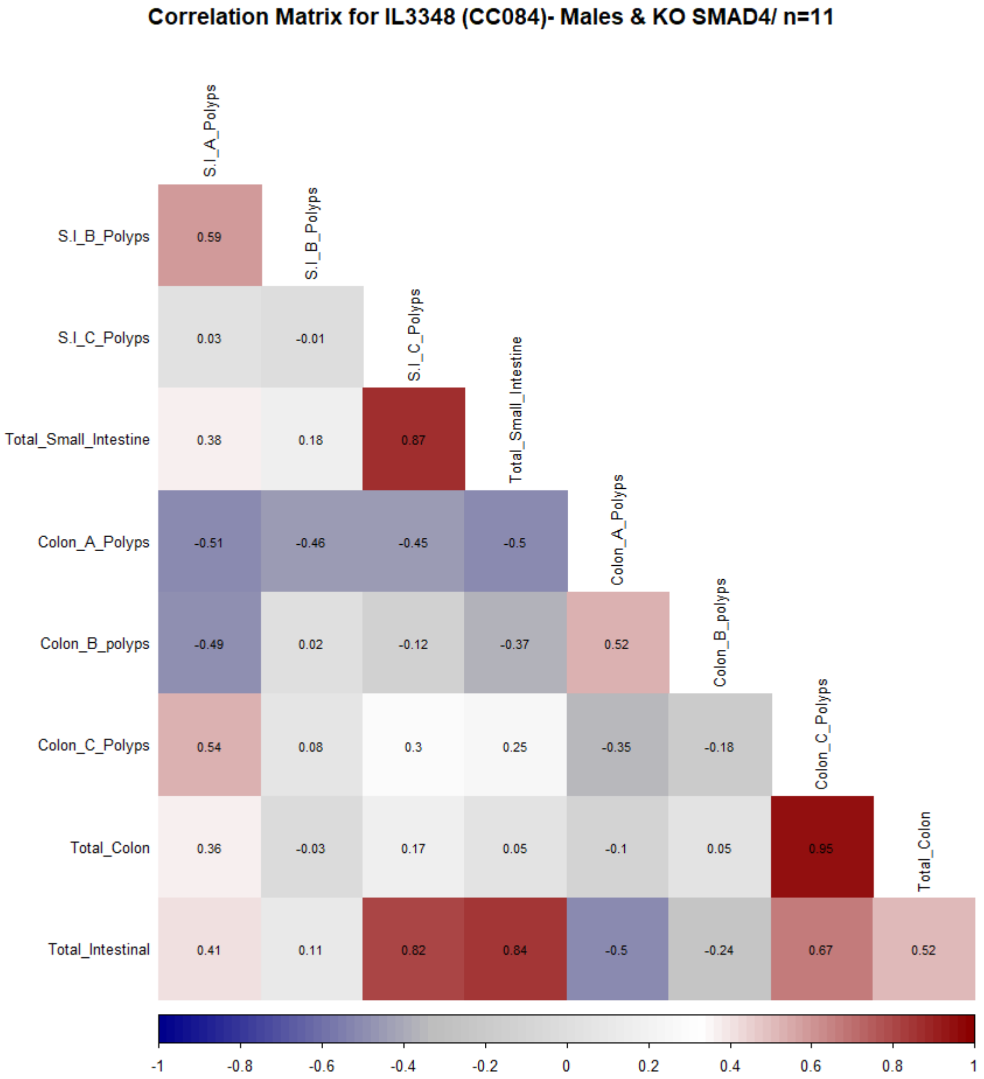

Supplementary Figure S36 Correlation Analysis of Polyp Development Patterns in HETEROZYGOUS KNOCK-OUT male Mice from line CC084.

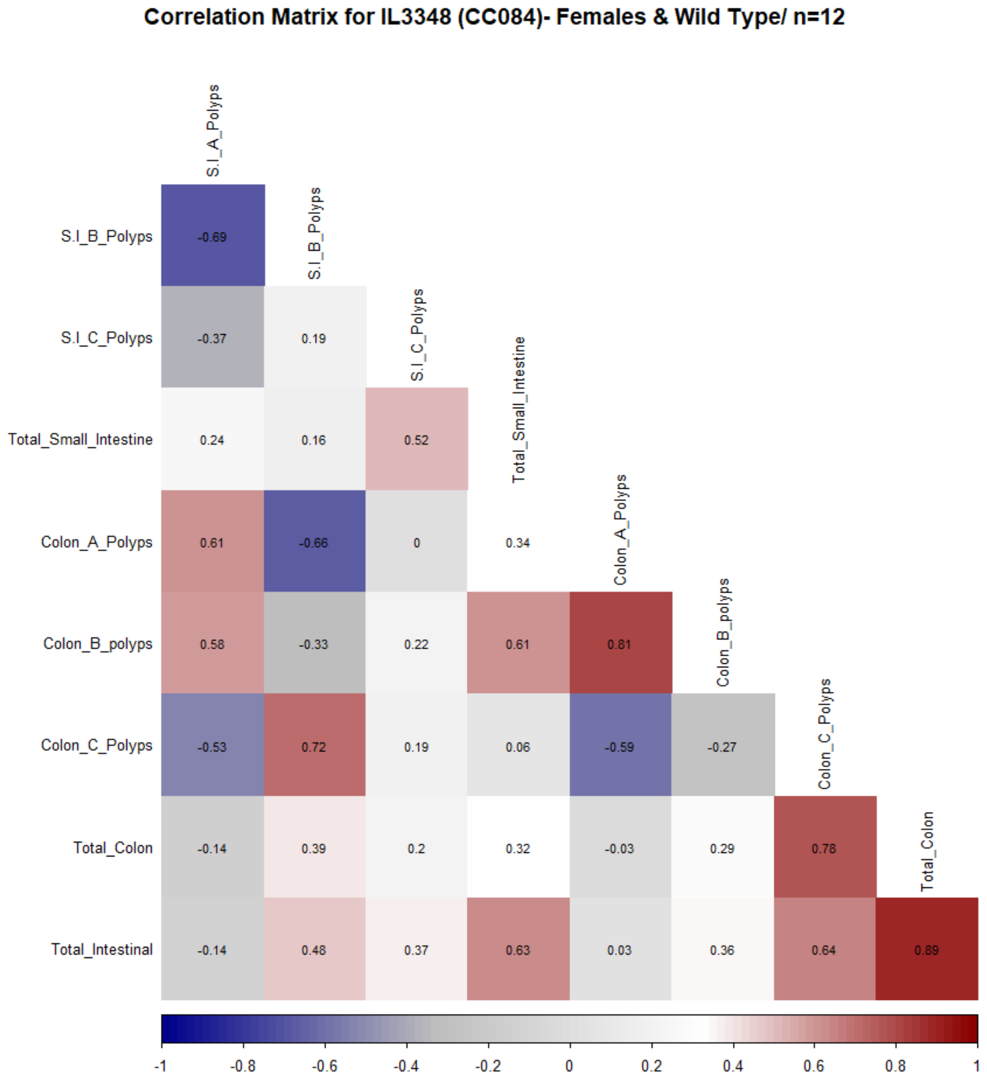

Supplementary Figure S37 Correlation Analysis of Polyp Development Patterns in Wild-Type female Mice from line CC084.

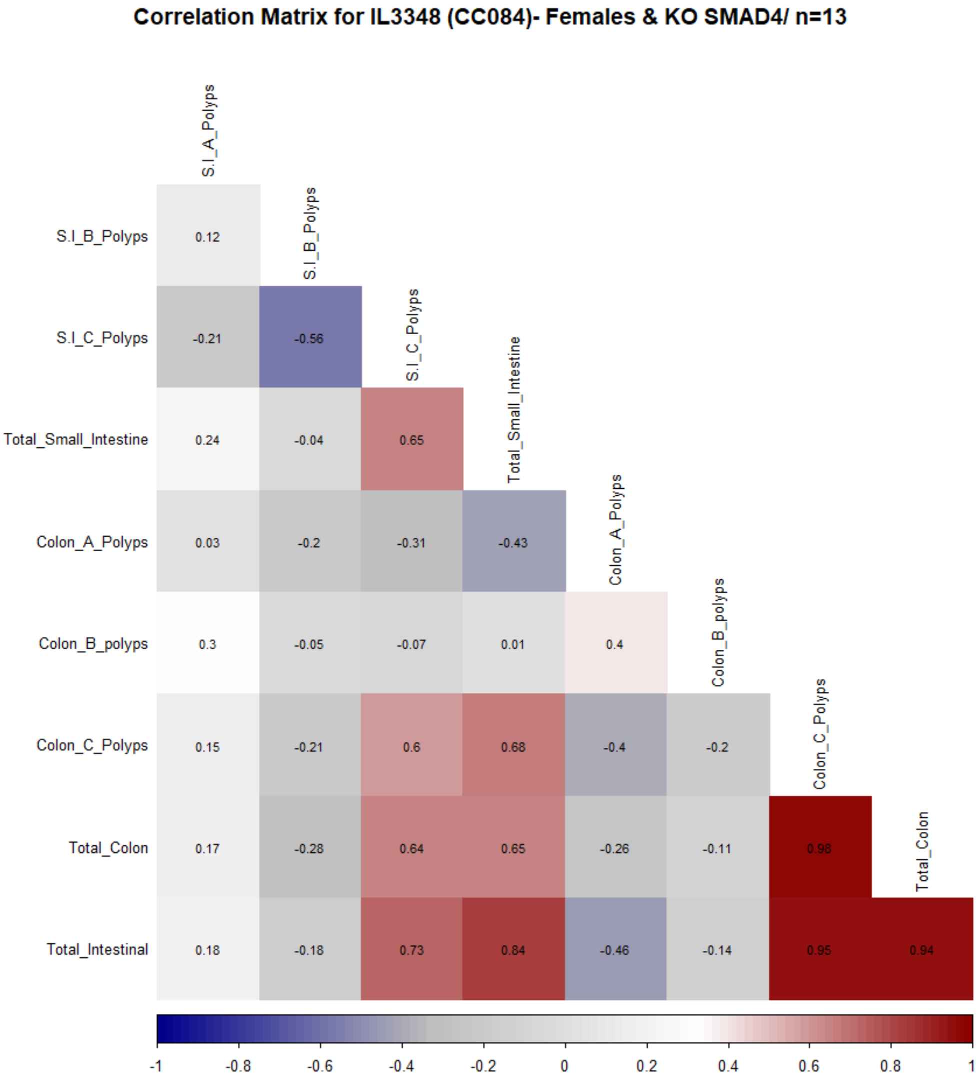

Supplementary Figure S38 Correlation Analysis of Polyp Development Patterns in HETEROZYGOUS KNOCK-OUT female Mice from line CC084.

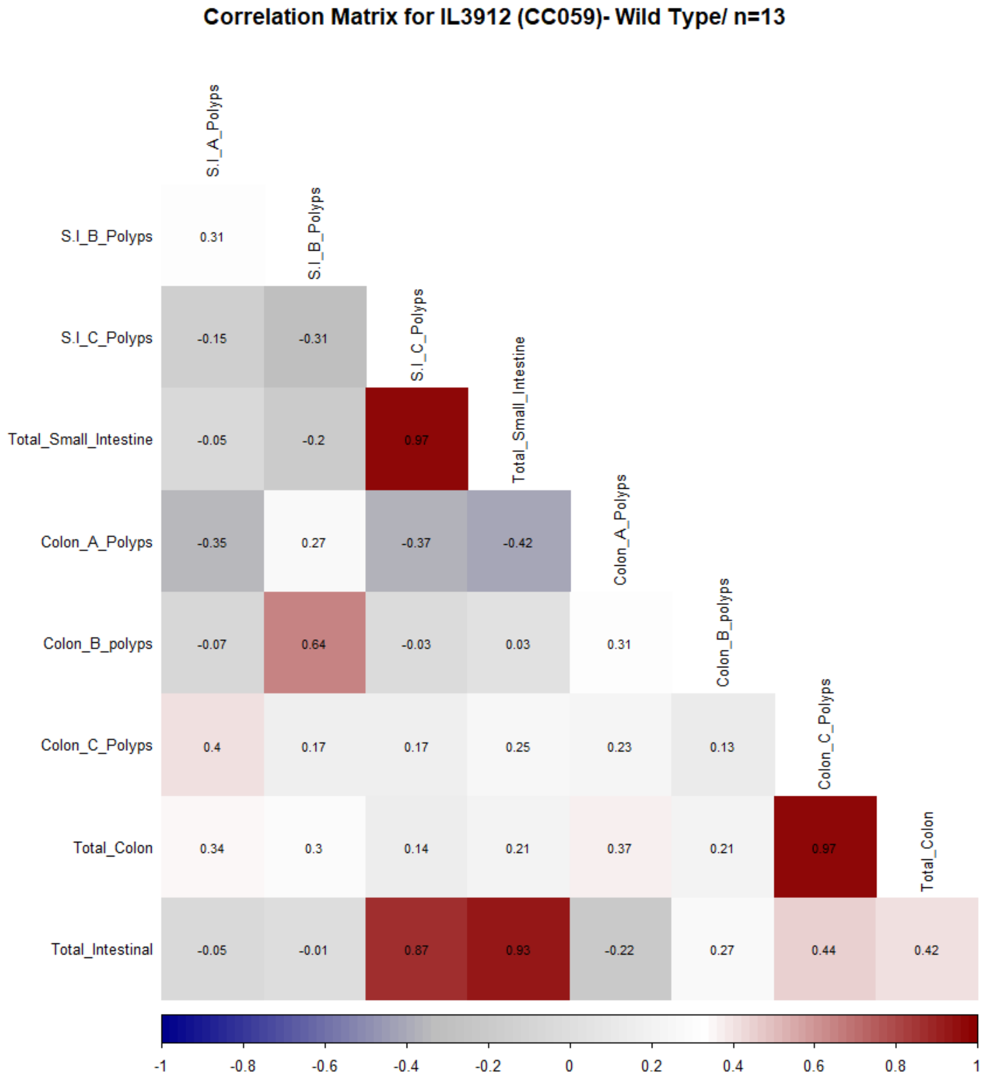

Supplementary Figure S39 Correlation Analysis of Polyp Development Patterns in Wild-Type Mice from line CC059.

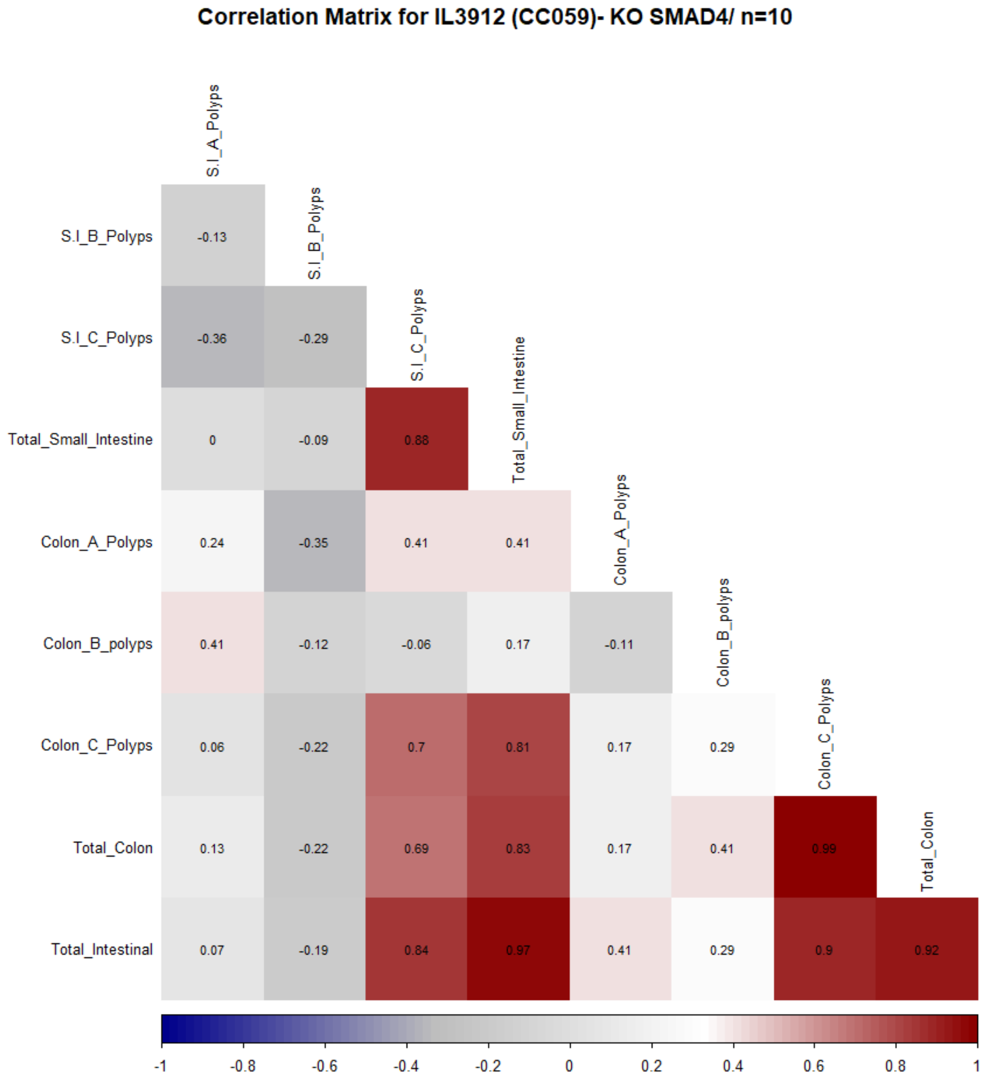

Supplementary Figure S40 Correlation Analysis of Polyp Development Patterns in HETEROZYGOUS KNOCK-OUT Mice from line CC059.

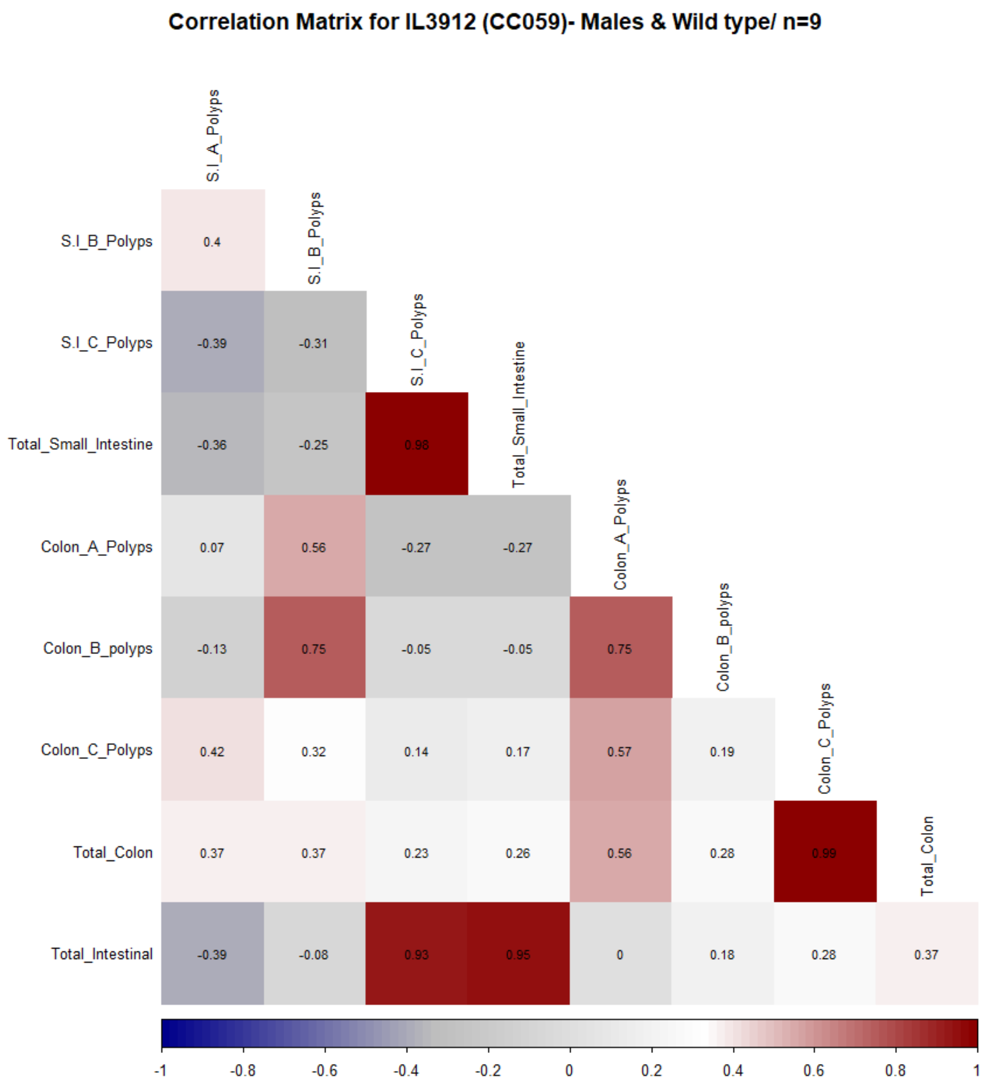

Supplementary Figure S41 Correlation Analysis of Polyp Development Patterns in Wild-Type male Mice from line CC059.

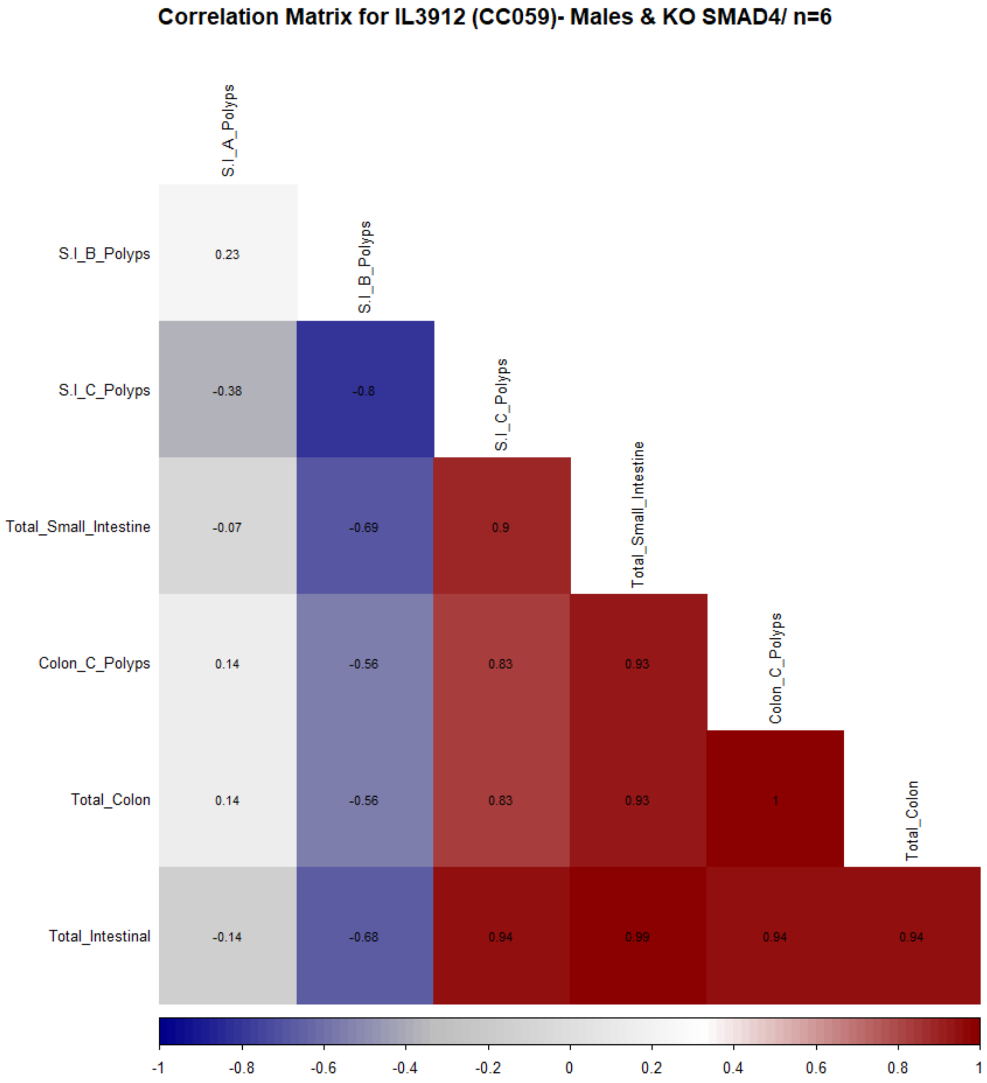

Supplementary Figure S42 Correlation Analysis of Polyp Development Patterns in HETEROZYGOUS KNOCK-OUT male Mice from line CC059.

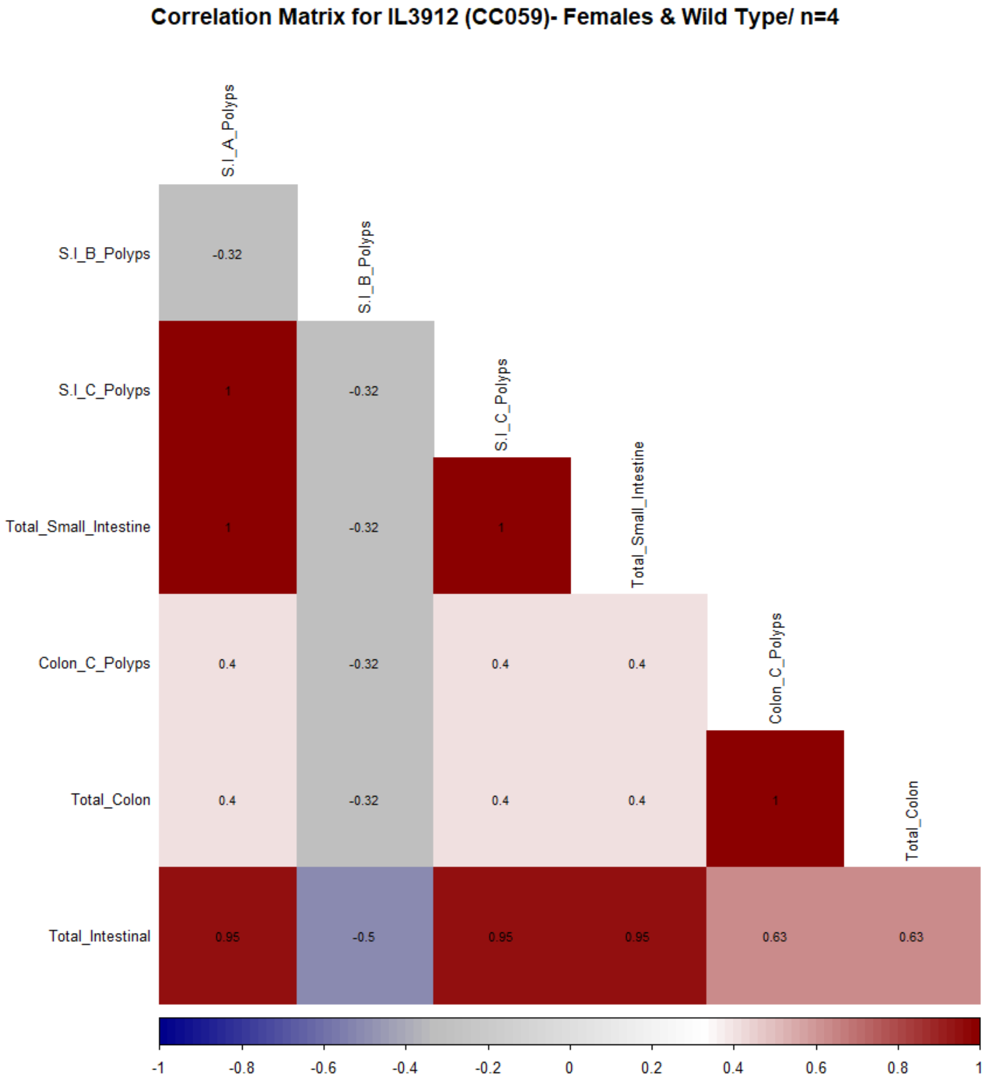

Supplementary Figure S43 Correlation Analysis of Polyp Development Patterns in Wild-Type female Mice from line CC059.

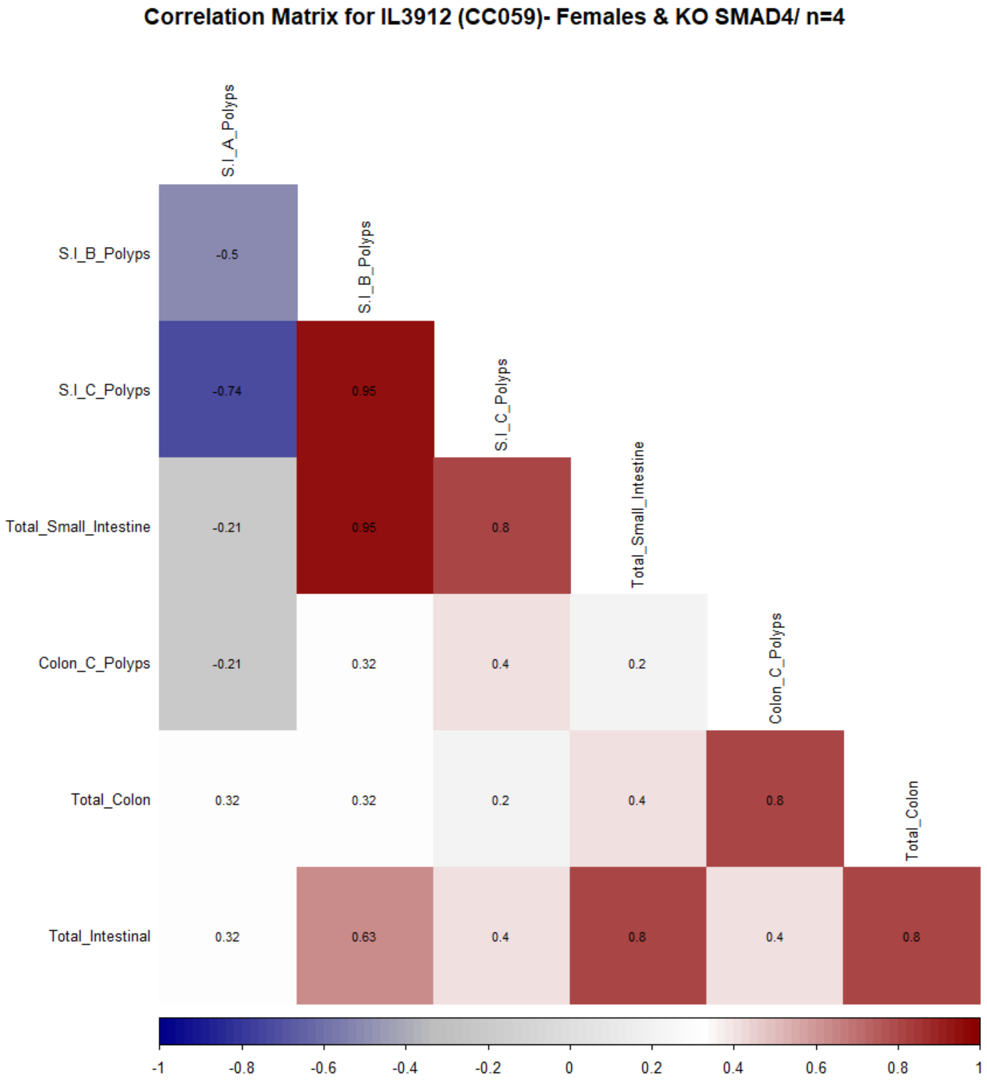

Supplementary Figure S44 Correlation Analysis of Polyp Development Patterns in HETEROZYGOUS KNOCK-OUT female Mice from line CC059.

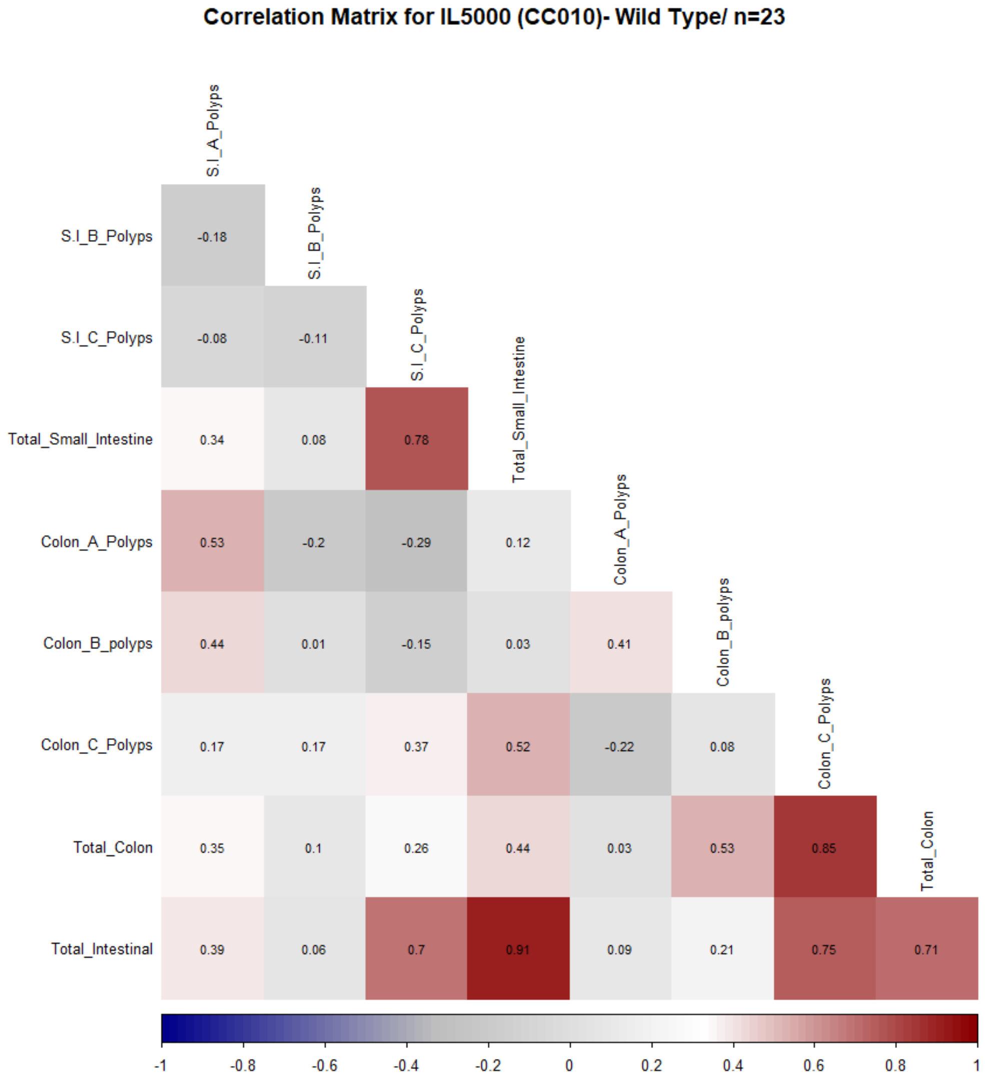

Supplementary Figure S45 Correlation Analysis of Polyp Development Patterns in Wild-Type Mice from line CC010.

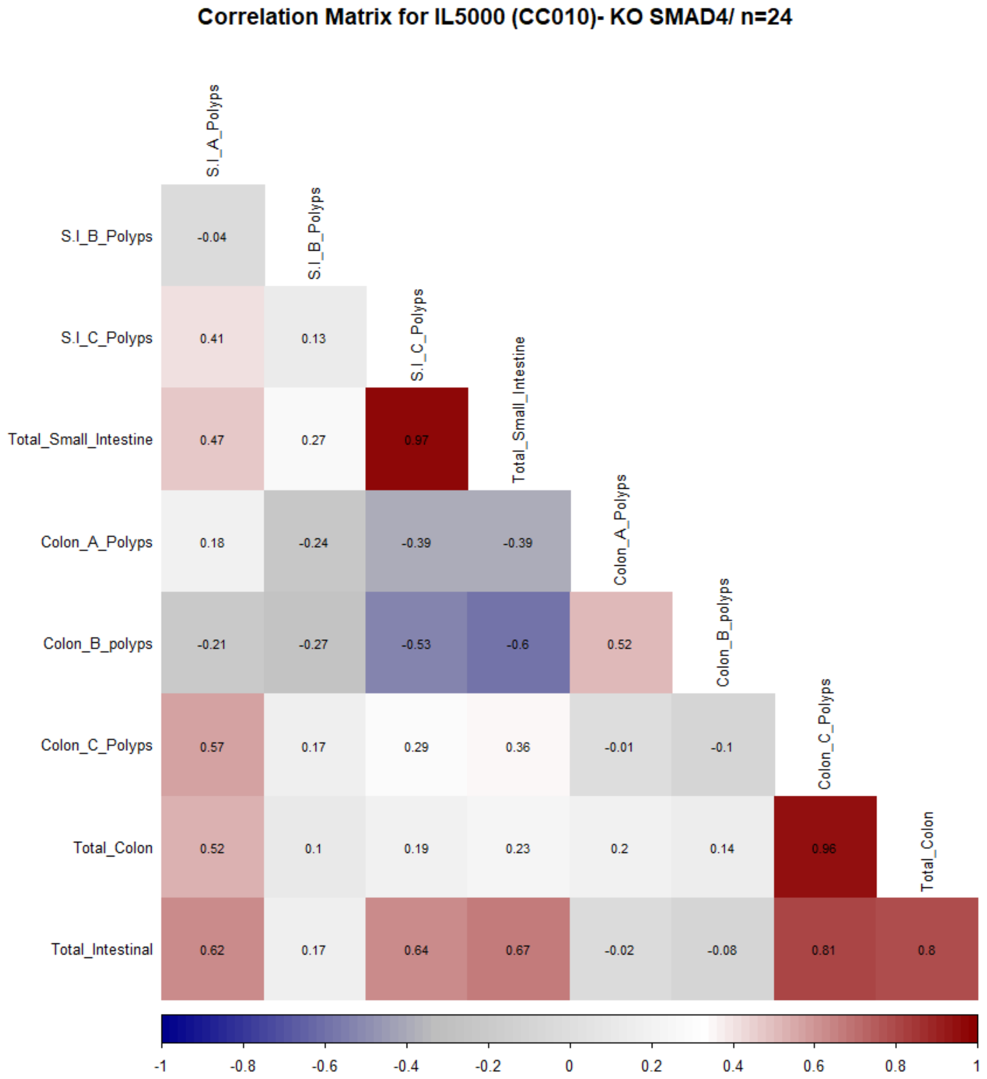

Supplementary Figure S46 Correlation Analysis of Polyp Development Patterns in HETEROZYGOUS KNOCK-OUT Mice from line CC010.

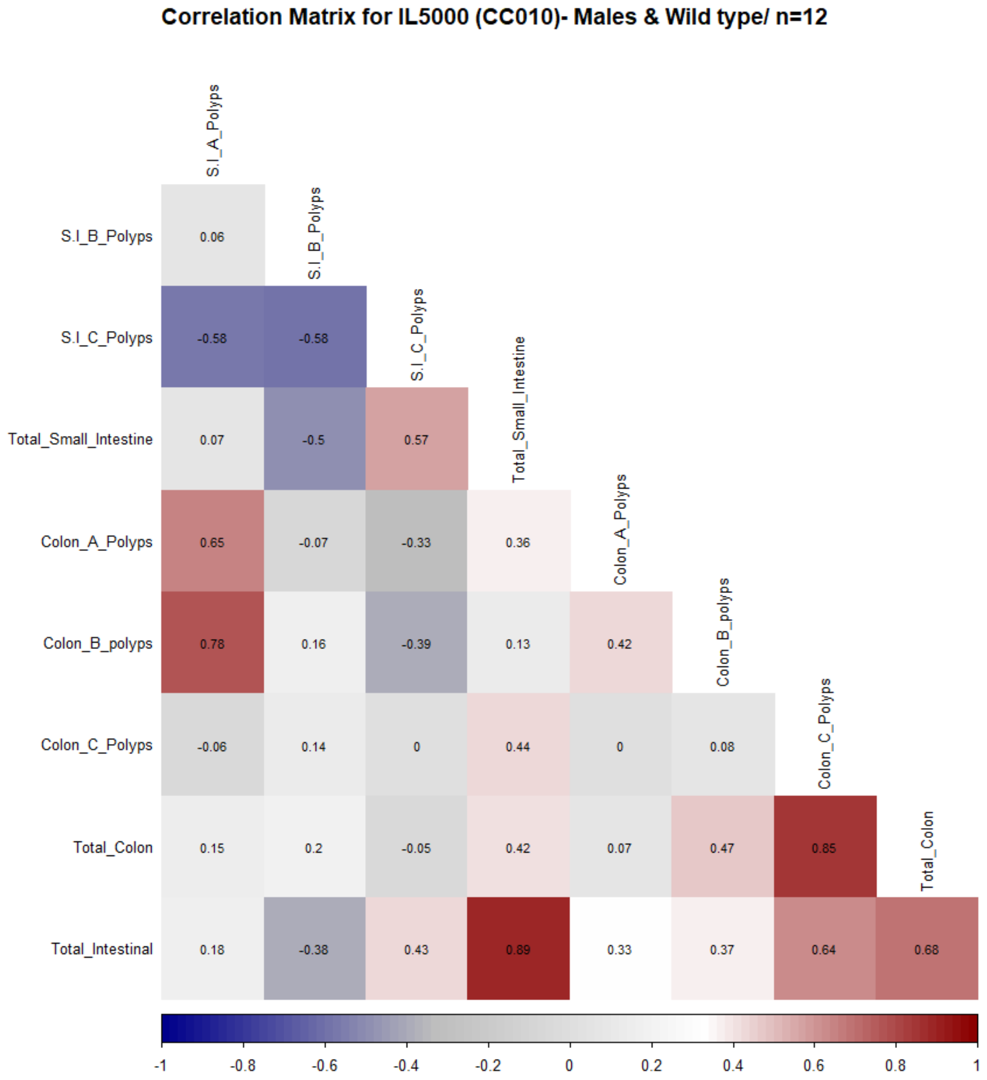

Supplementary Figure S47 Correlation Analysis of Polyp Development Patterns in Wild-Type male Mice from line CC010.

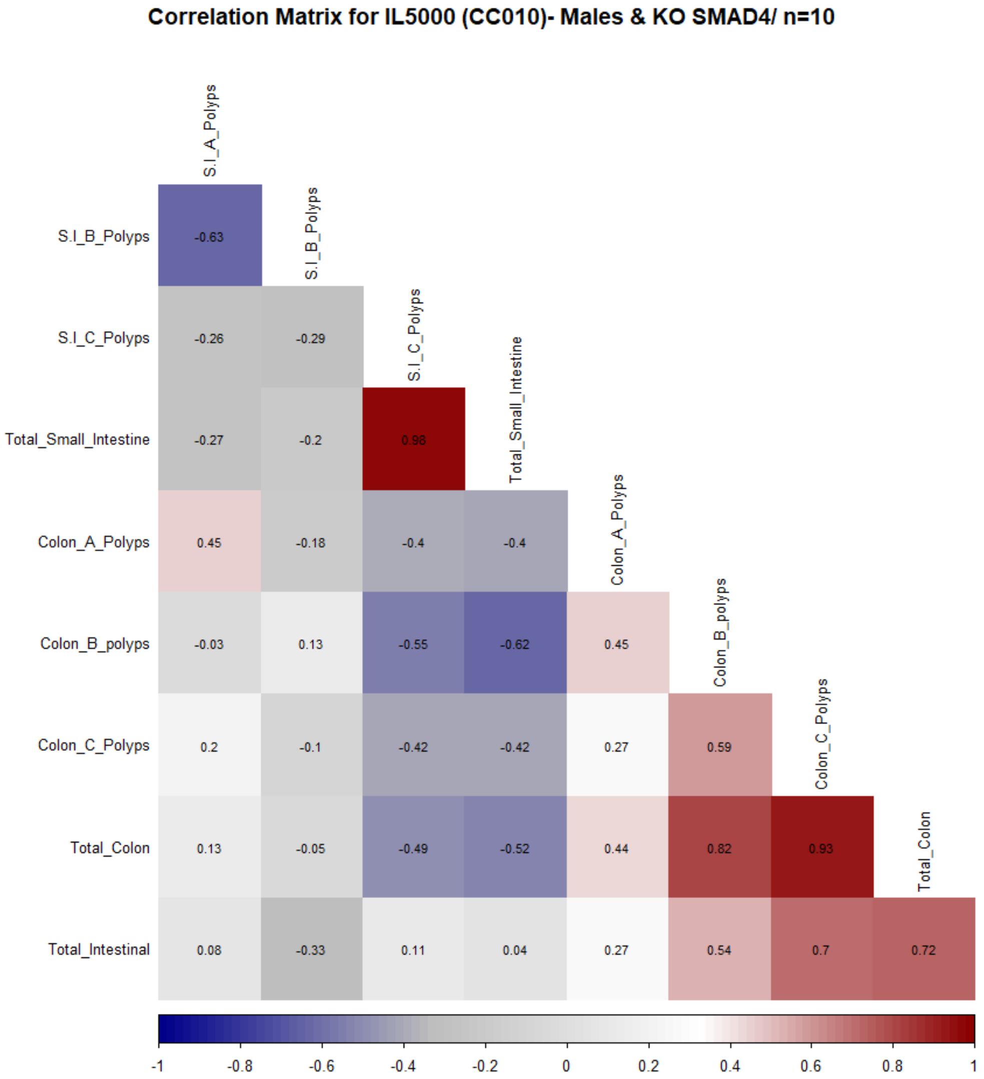

Supplementary Figure S48 Correlation Analysis of Polyp Development Patterns in HETEROZYGOUS KNOCK-OUT male Mice from line CC010.

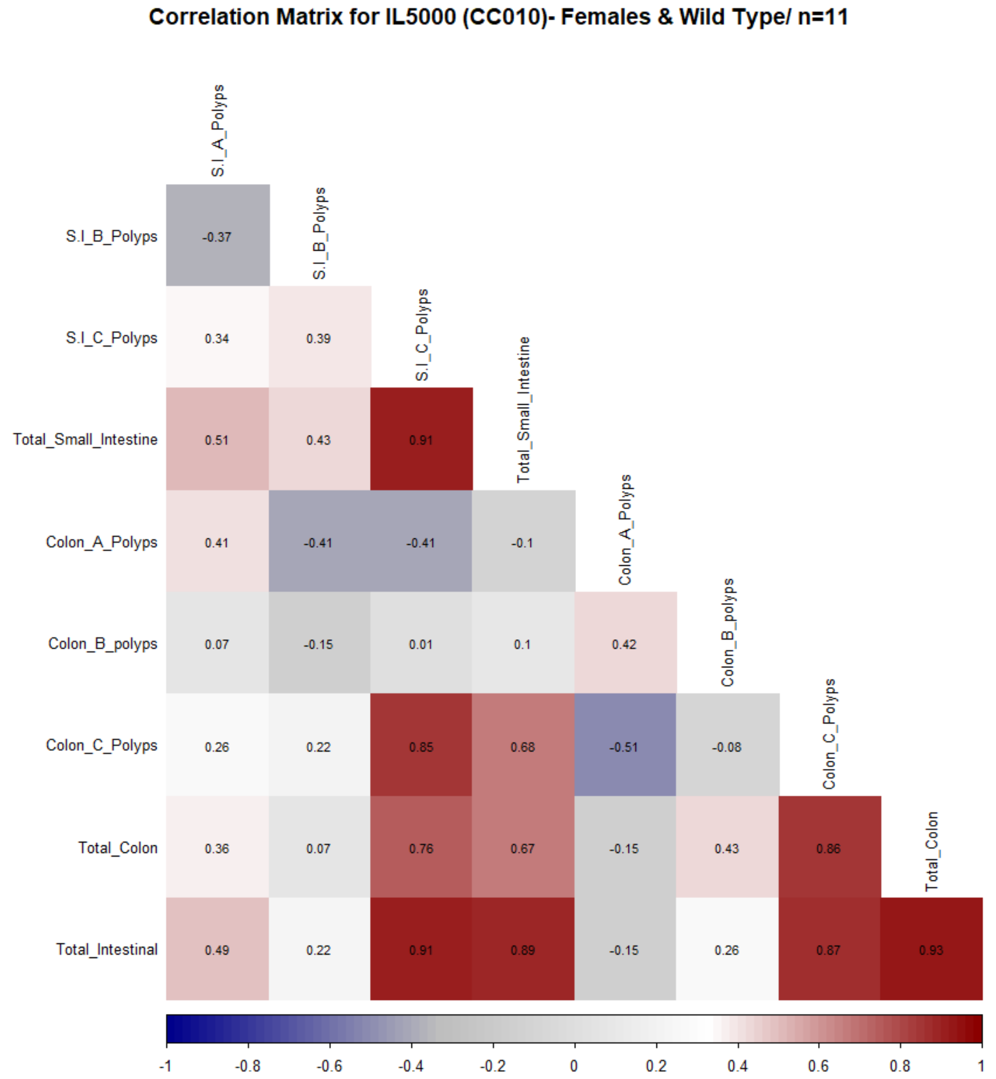

Supplementary Figure S49 Correlation Analysis of Polyp Development Patterns in Wild-Type female Mice from line CC010.

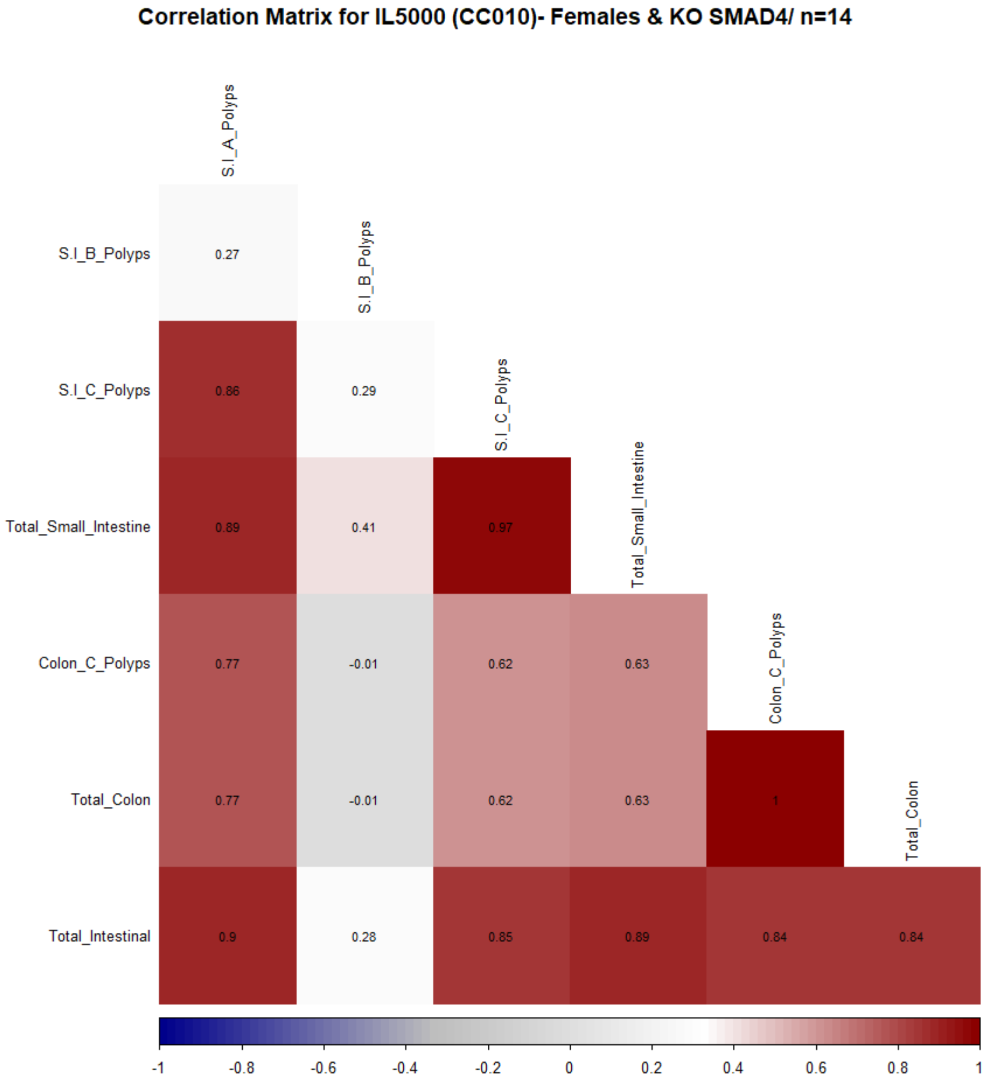

Supplementary Figure S50 Correlation Analysis of Polyp Development Patterns in HETEROZYGOUS KNOCK-OUT female Mice from line CC010.

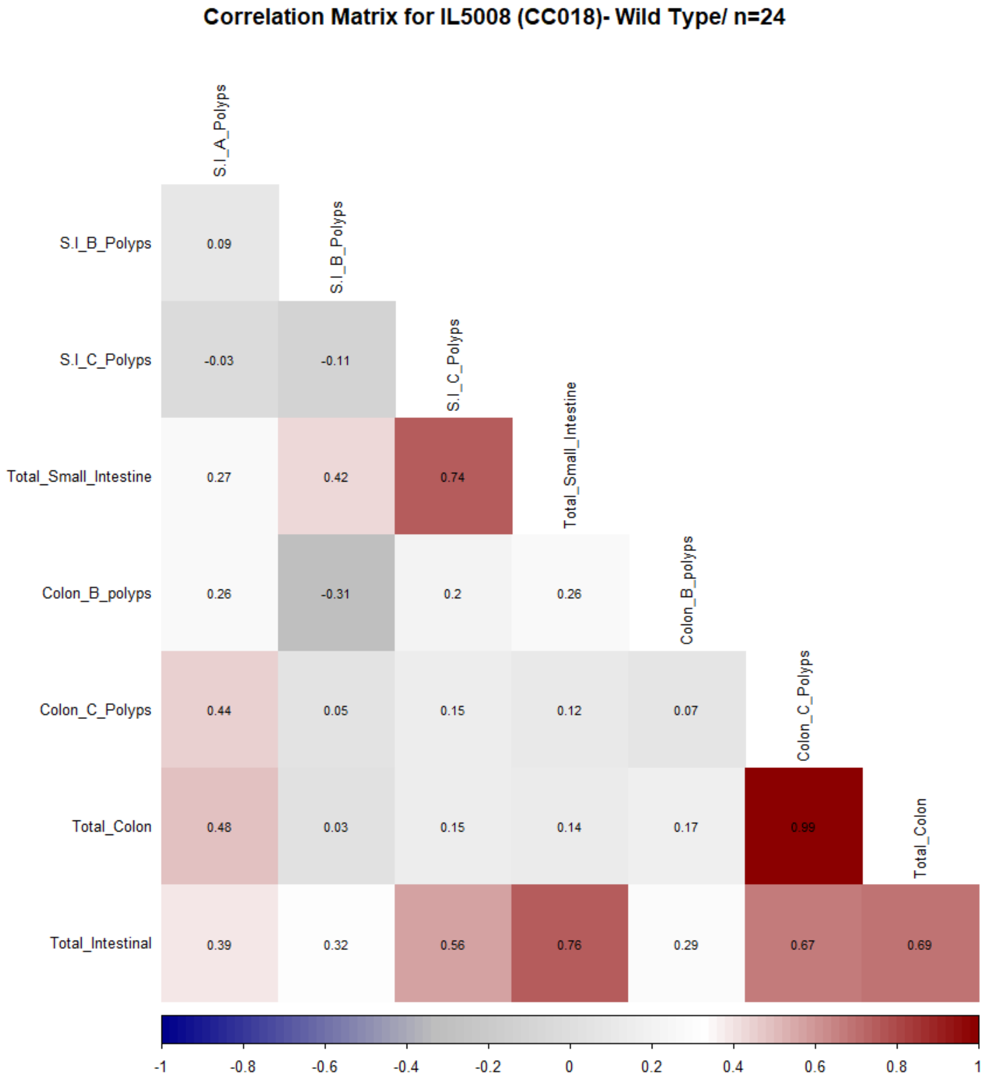

Supplementary Figure S51 Correlation Analysis of Polyp Development Patterns in Wild-Type Mice from line CC018.

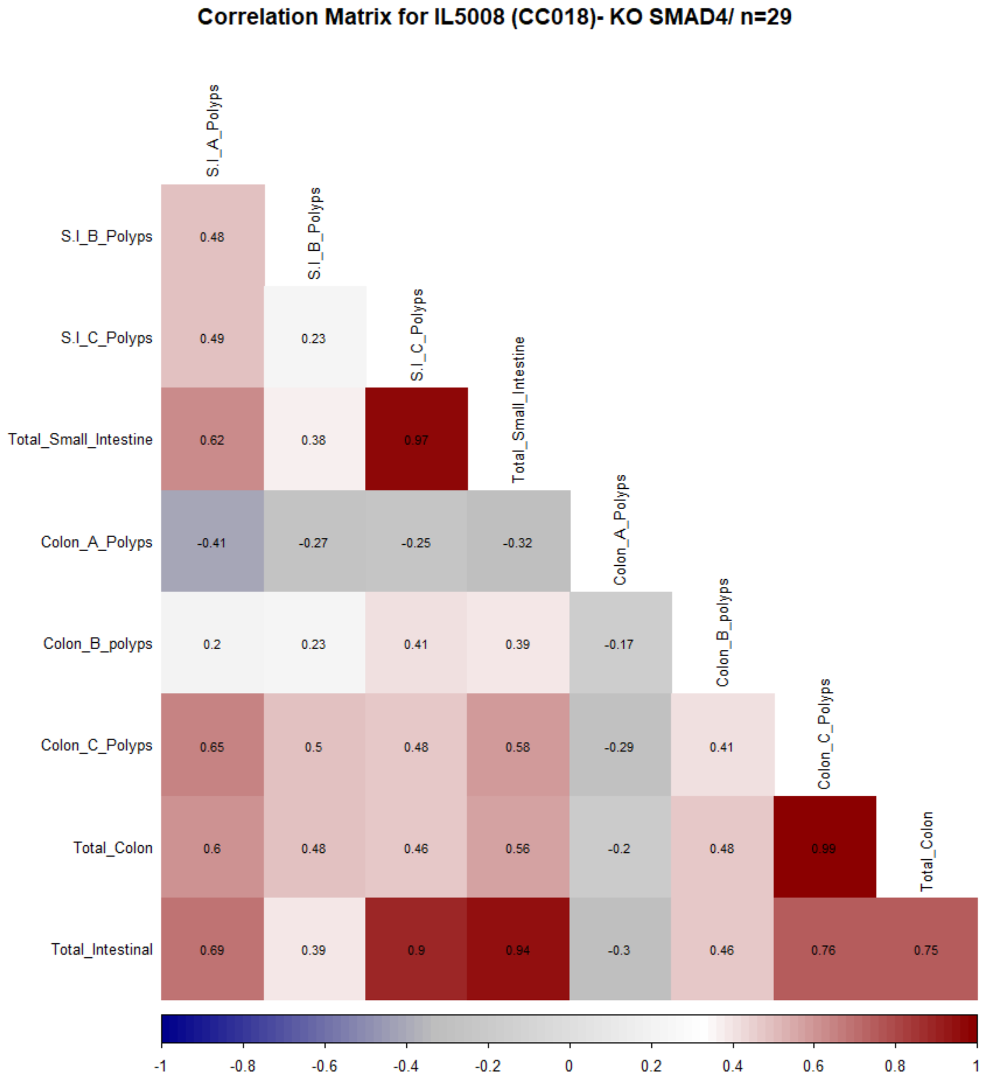

Supplementary Figure S52 Correlation Analysis of Polyp Development Patterns in HETEROZYGOUS KNOCK-OUT Mice from line CC018.

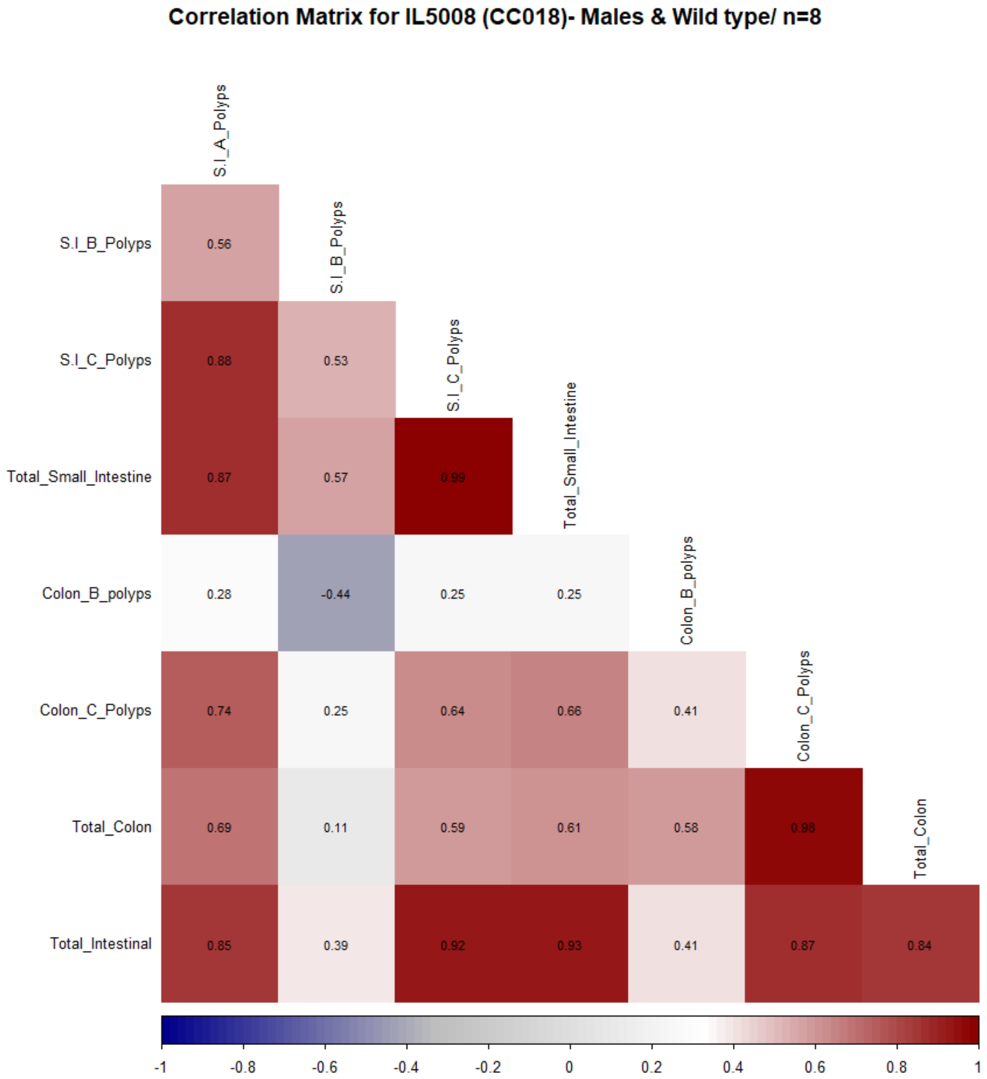

Supplementary Figure S53 Correlation Analysis of Polyp Development Patterns in Wild-Type male Mice from line CC018.

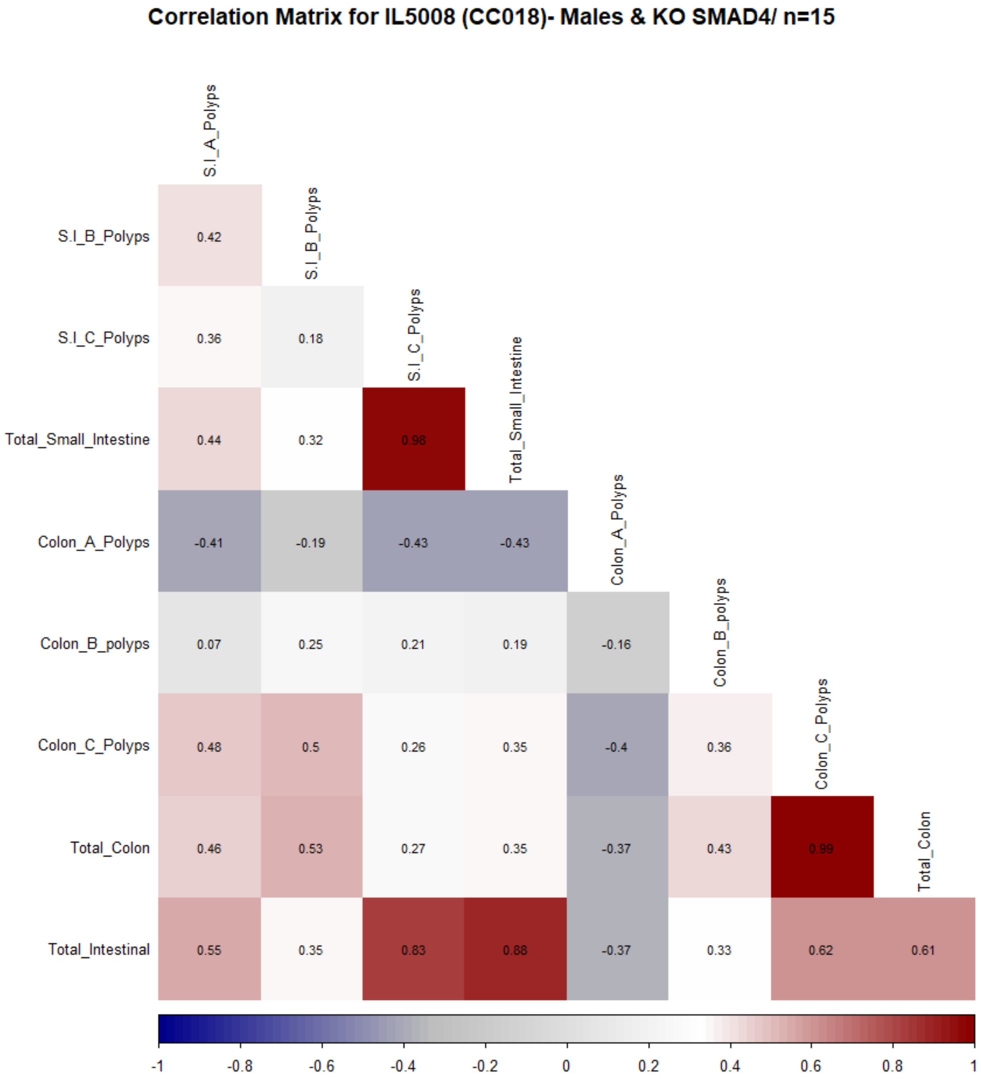

Supplementary Figure S54 Correlation Analysis of Polyp Development Patterns in Heterozygous Knock-Out male Mice from line CC018.

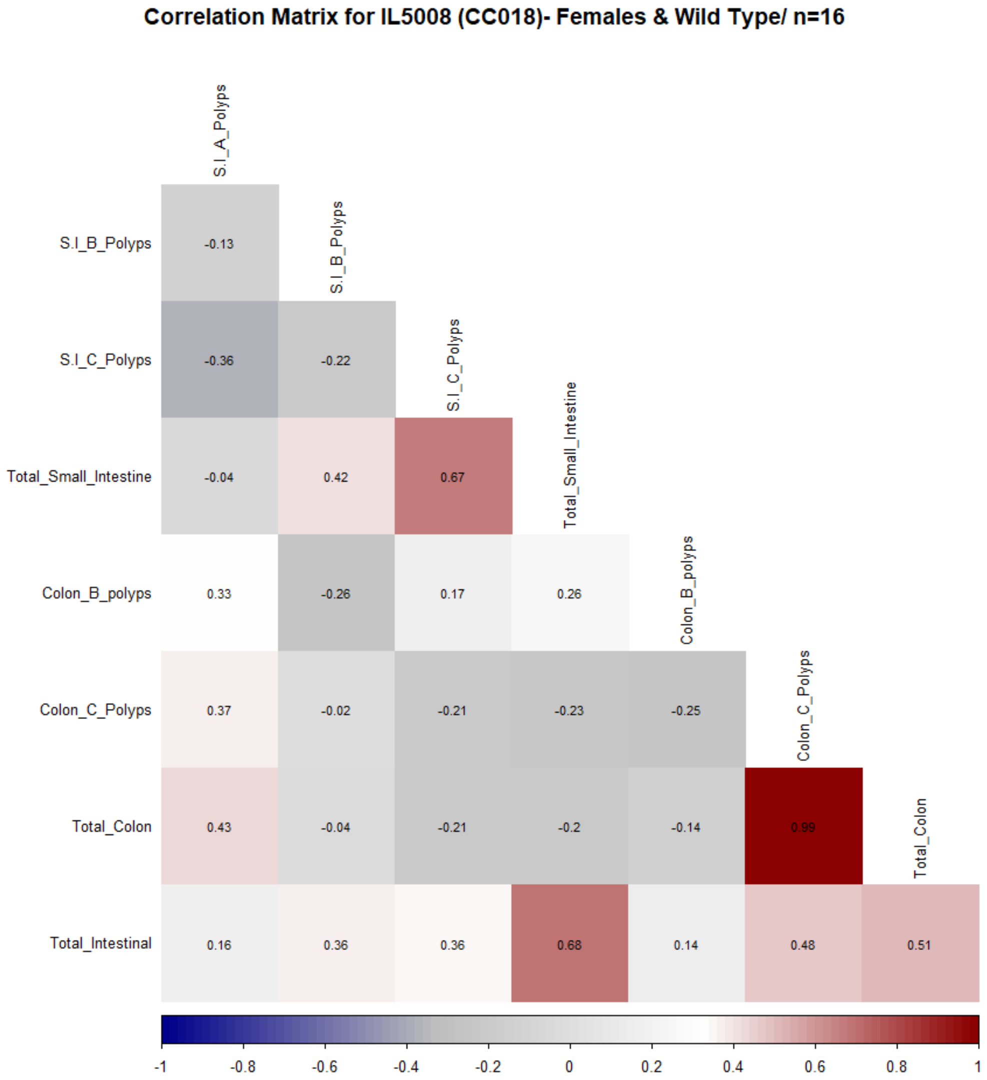

Supplementary Figure S55 Correlation Analysis of Polyp Development Patterns in Wild-Type female Mice from line CC018.

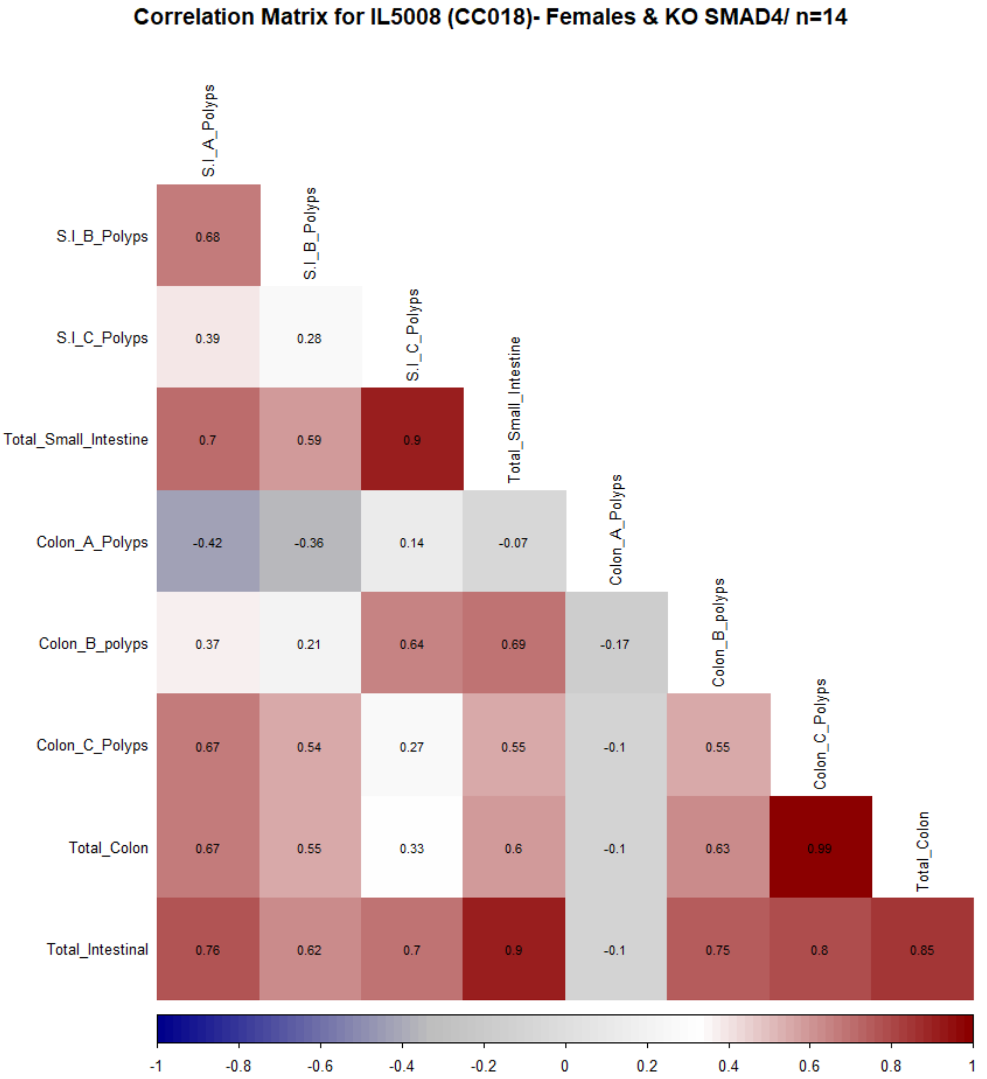

Supplementary Figure S56 Correlation Analysis of Polyp Development Patterns in HETEROZYGOUS KNOCK-OUT female Mice from line CC018.

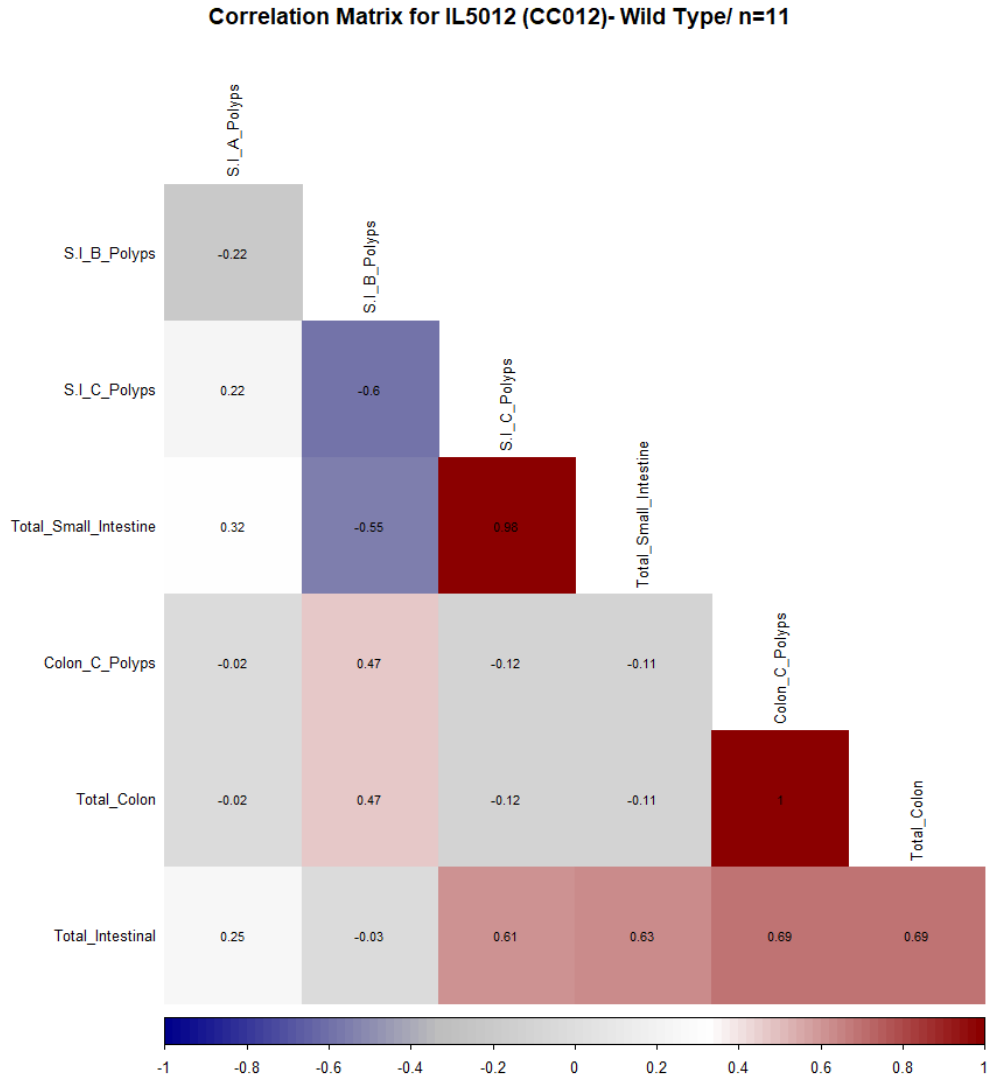

Supplementary Figure S57 Correlation Analysis of Polyp Development Patterns in Wild-Type Mice from line CC012.

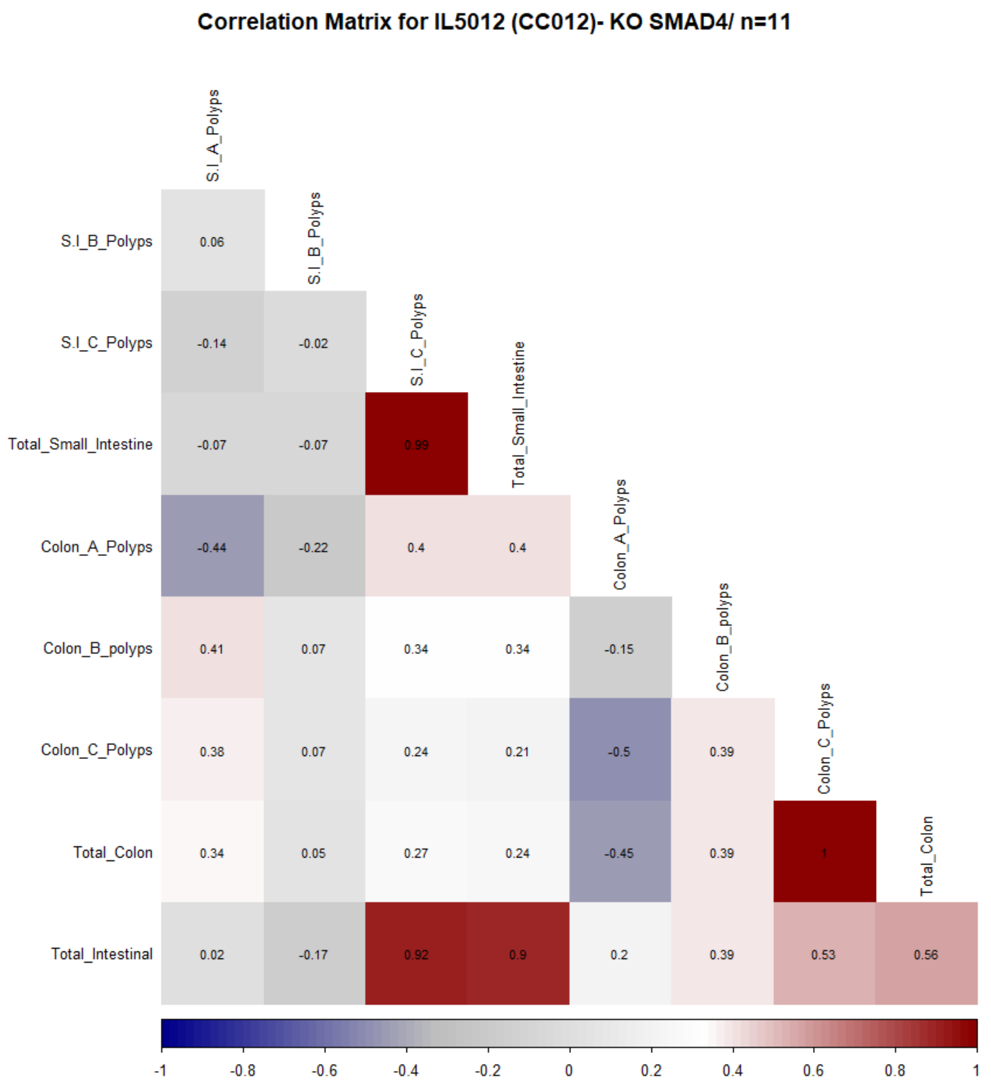

Supplementary Figure S58 Correlation Analysis of Polyp Development Patterns in HETEROZYGOUS KNOCK-OUT Mice from line CC012.

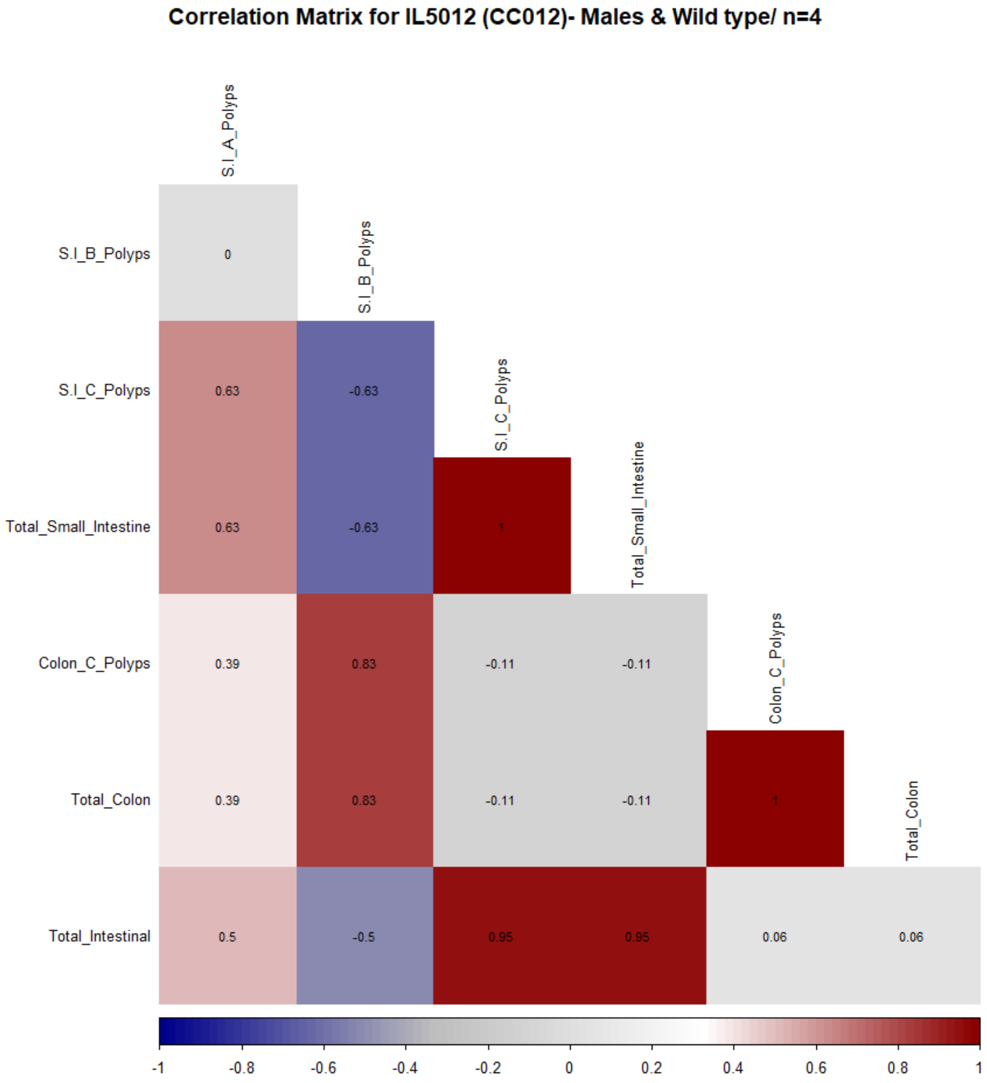

Supplementary Figure S59 Correlation Analysis of Polyp Development Patterns in Wild-Type male Mice from line CC012.

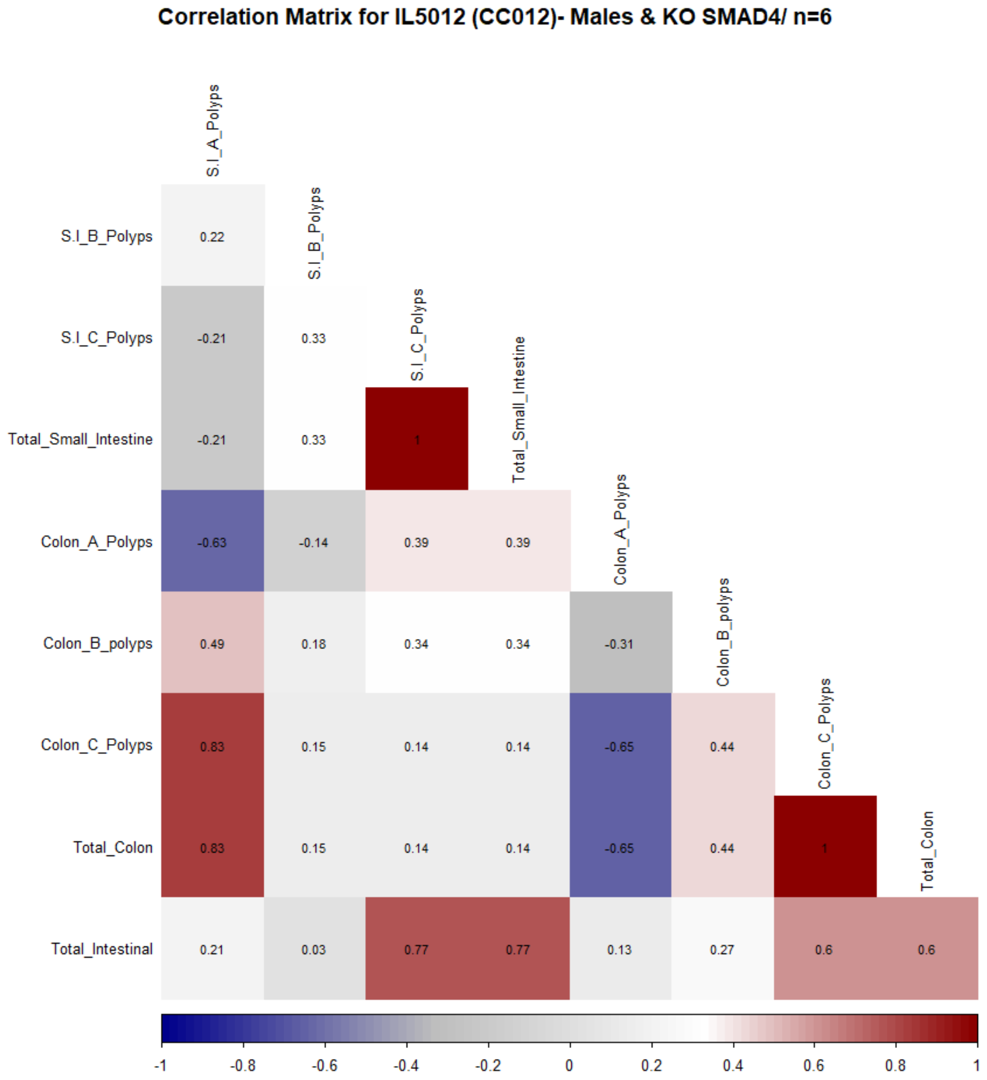

Supplementary Figure S60 Correlation Analysis of Polyp Development Patterns in HETEROZYGOUS KNOCK-OUT male Mice from line CC012.

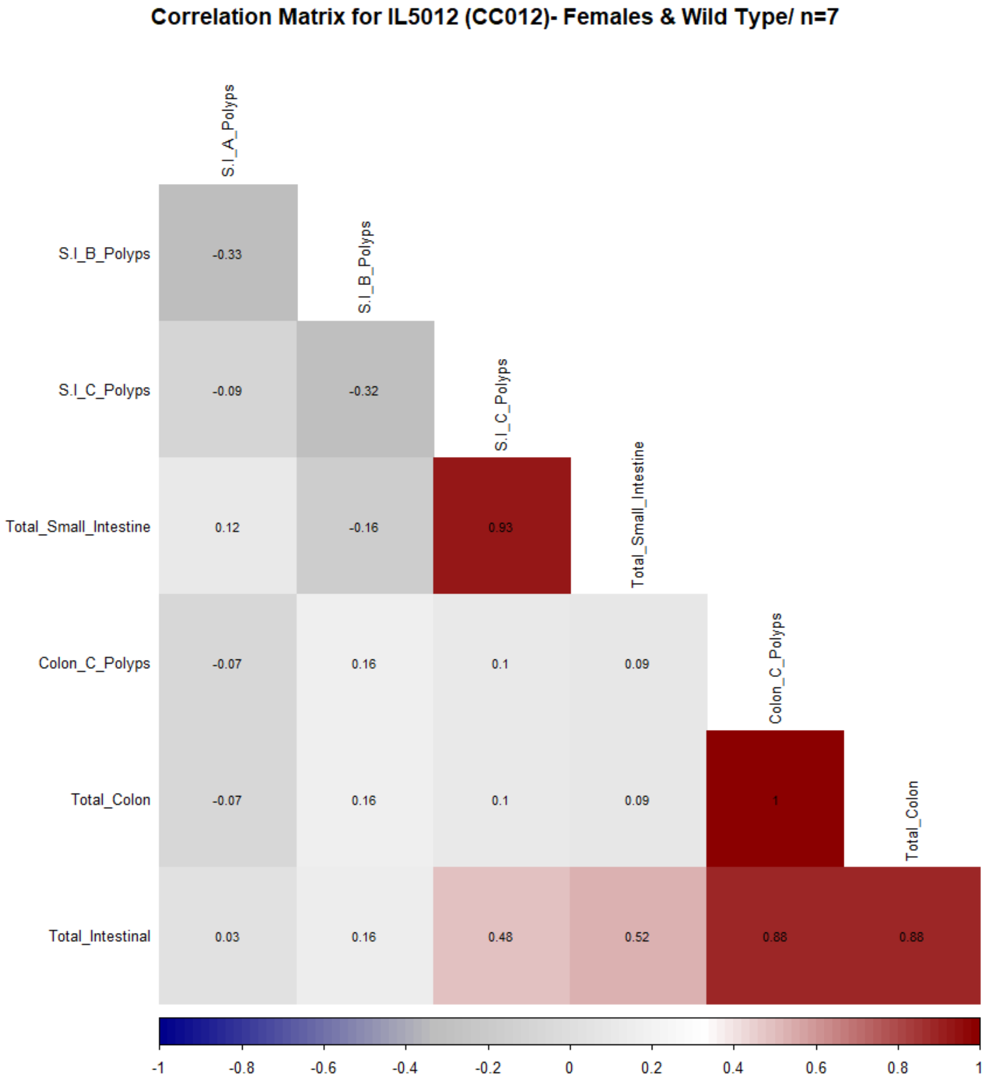

Supplementary Figure S61 Correlation Analysis of Polyp Development Patterns in Wild-Type female Mice from line CC012.

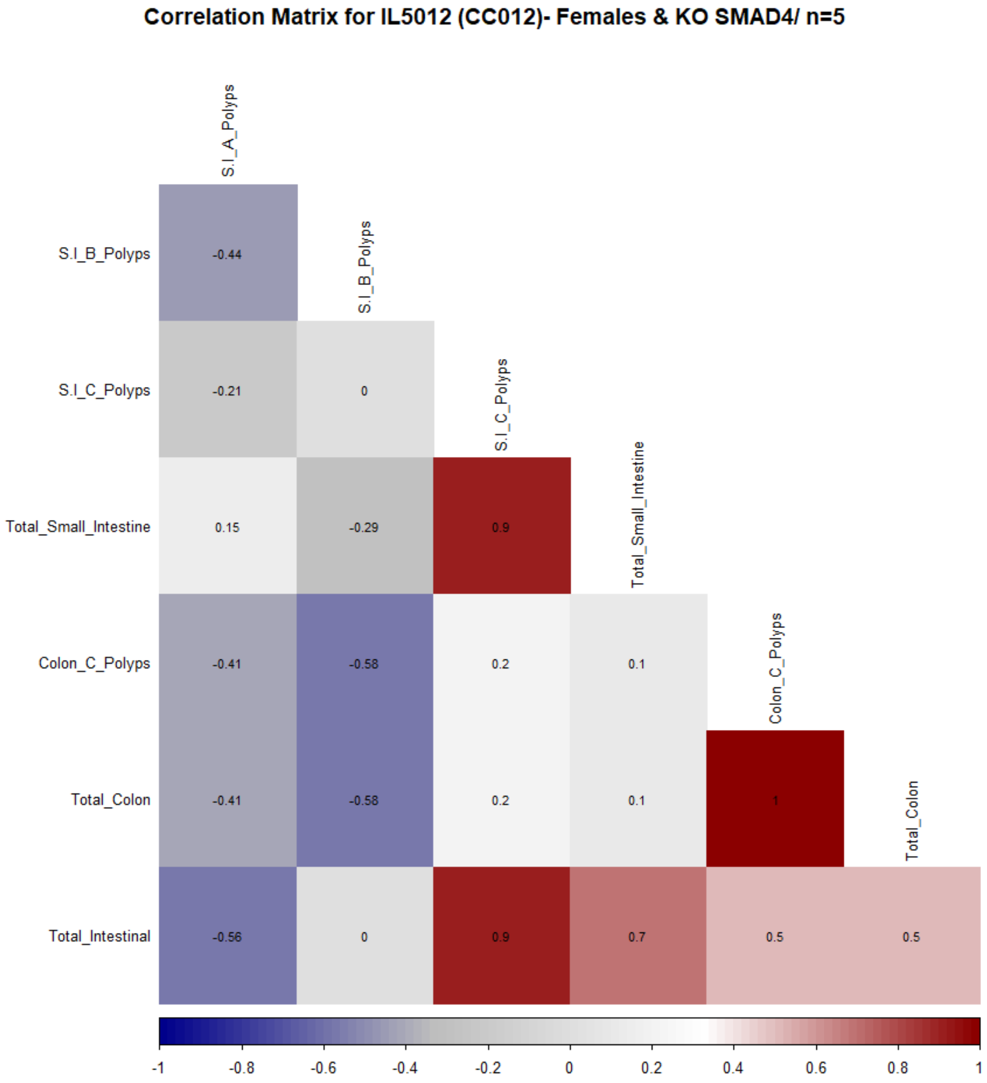

Supplementary Figure S62 Correlation Analysis of Polyp Development Patterns in HETEROZYGOUS KNOCK-OUT female Mice from line CC012.

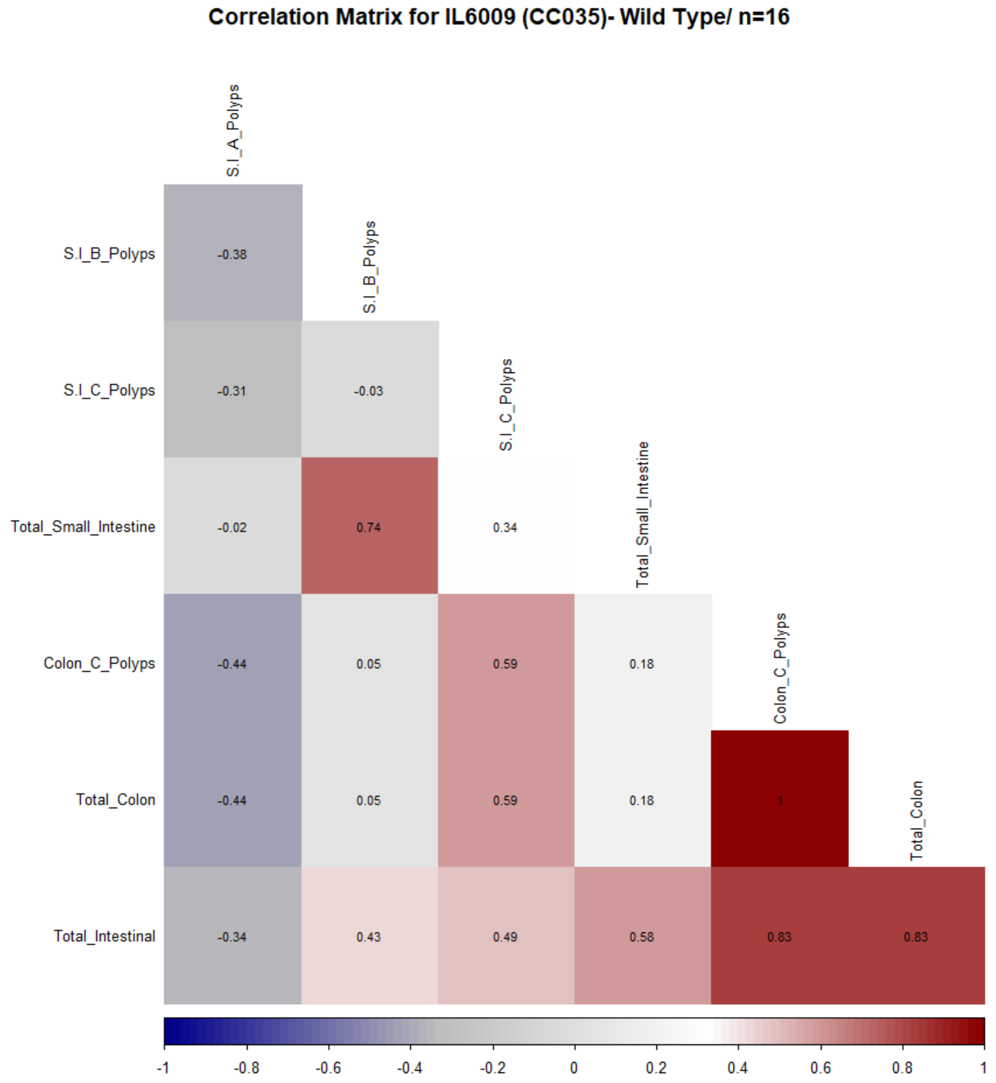

Supplementary Figure S63 Correlation Analysis of Polyp Development Patterns in Wild-Type Mice from line CC035.

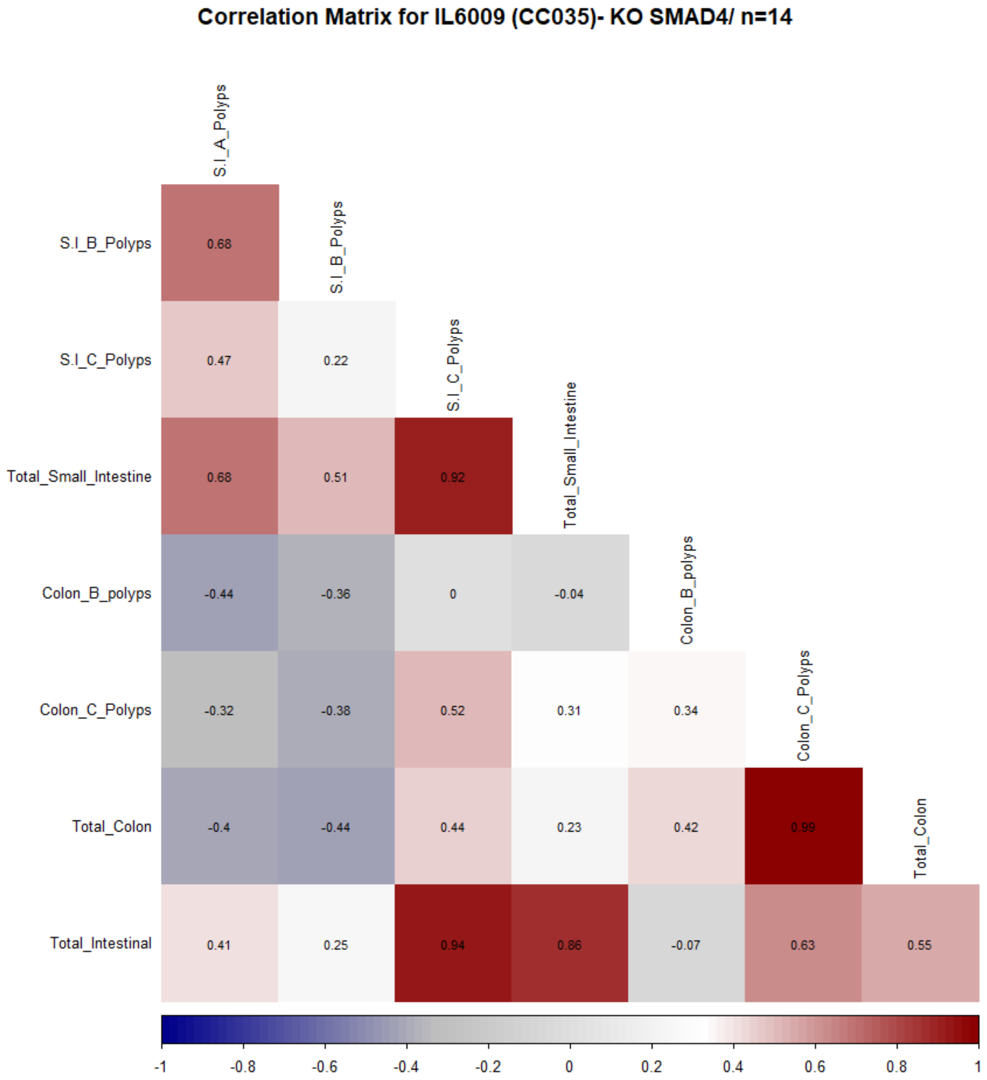

Supplementary Figure S64 Correlation Analysis of Polyp Development Patterns in HETEROZYGOUS KNOCK-OUT Mice from line CC035.

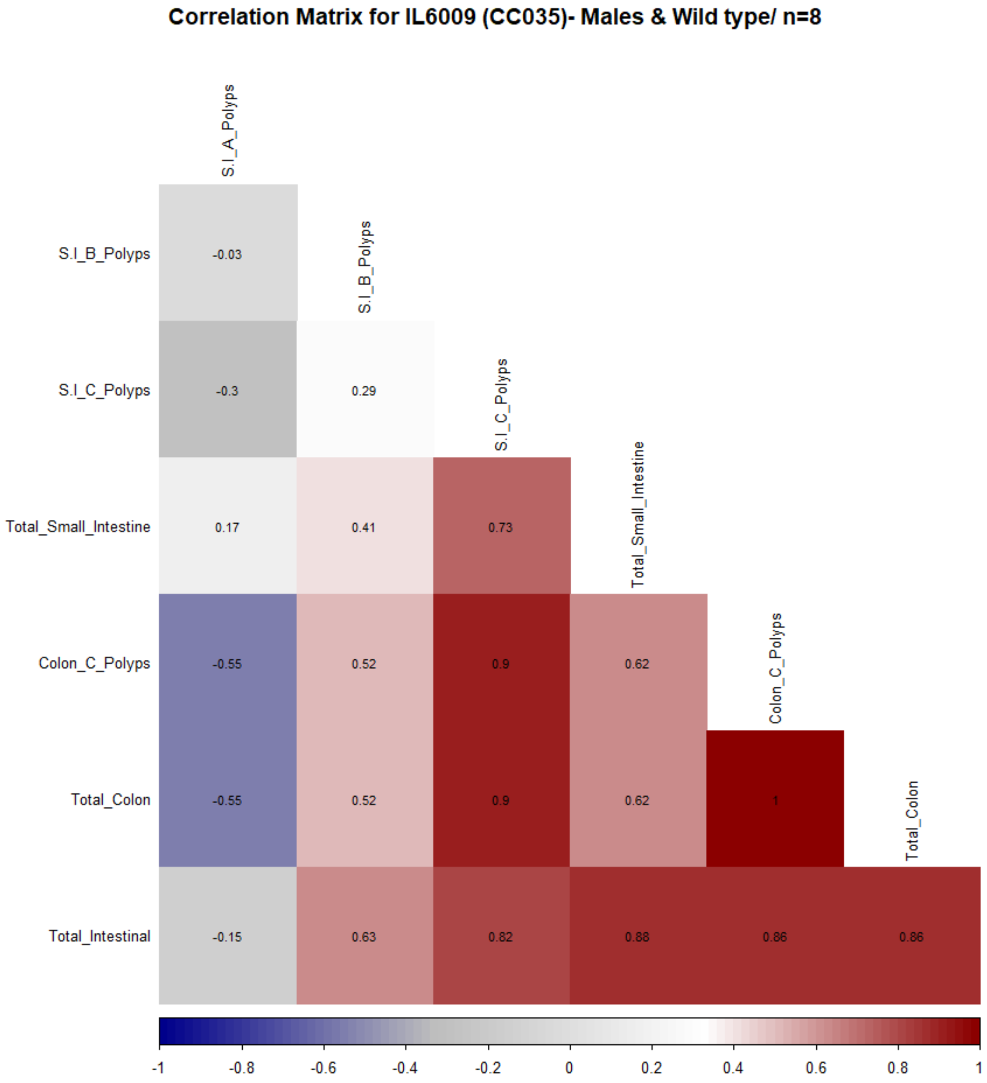

Supplementary Figure S65 Correlation Analysis of Polyp Development Patterns in Wild-Type male Mice from line CC035.

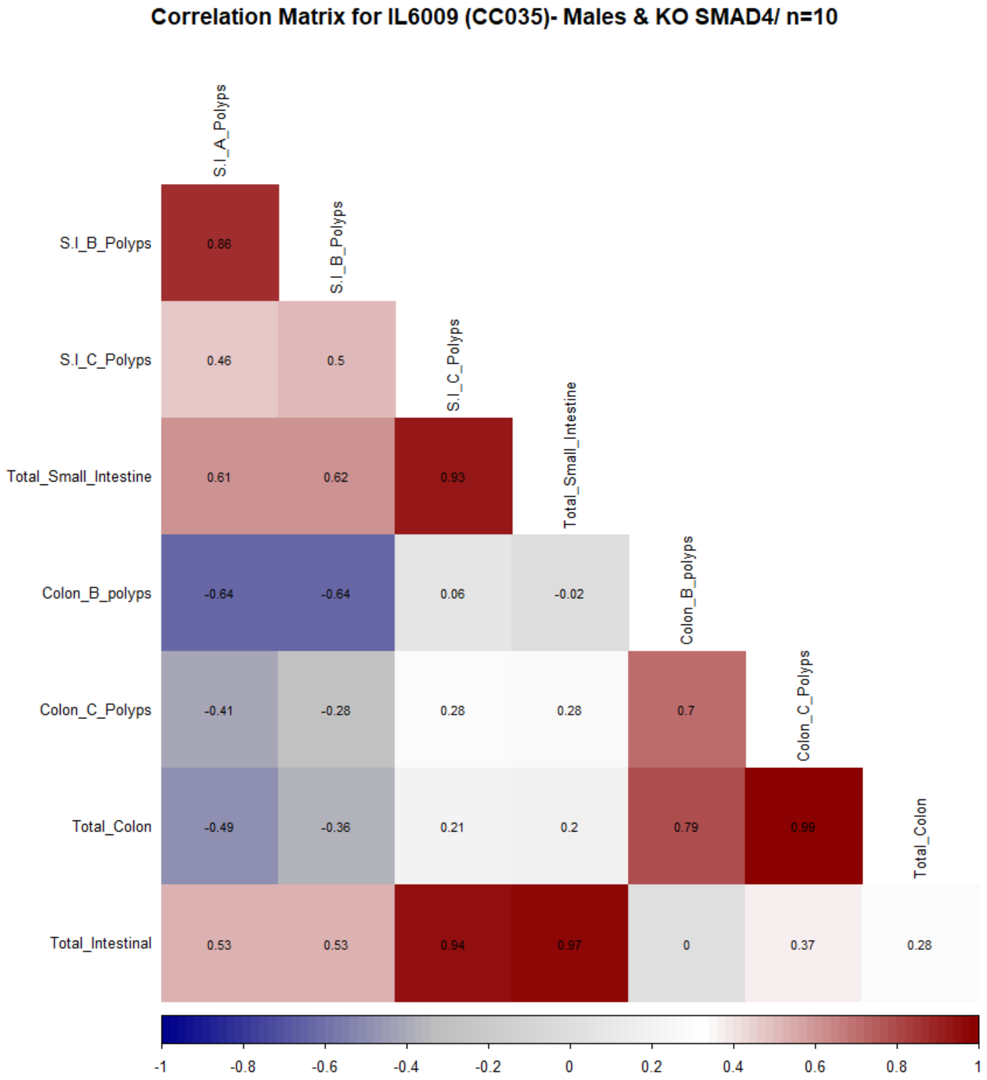

Supplementary Figure S66 Correlation Analysis of Polyp Development Patterns in HETEROZYGOUS KNOCK-OUT male Mice from line CC035.

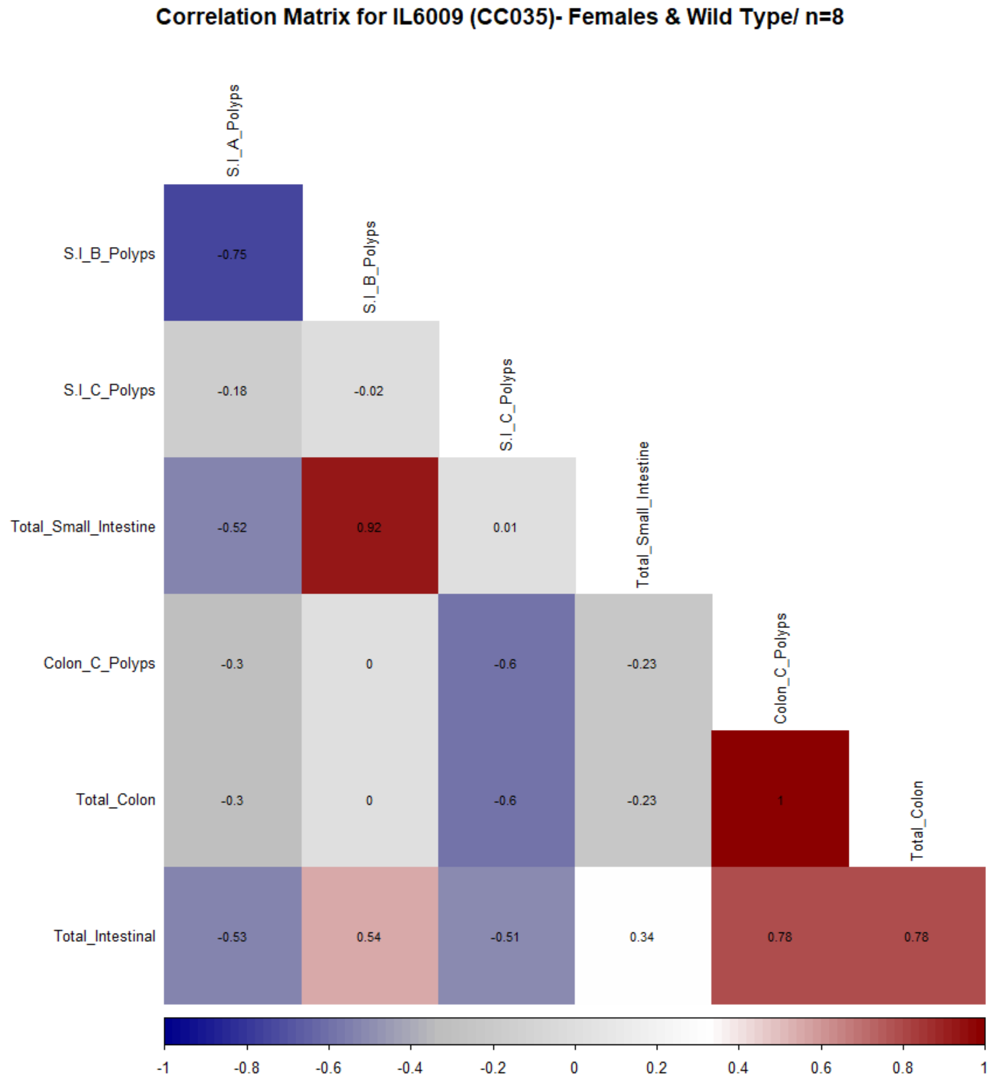

Supplementary Figure S67 Correlation Analysis of Polyp Development Patterns in Wild-Type female Mice from line CC035.

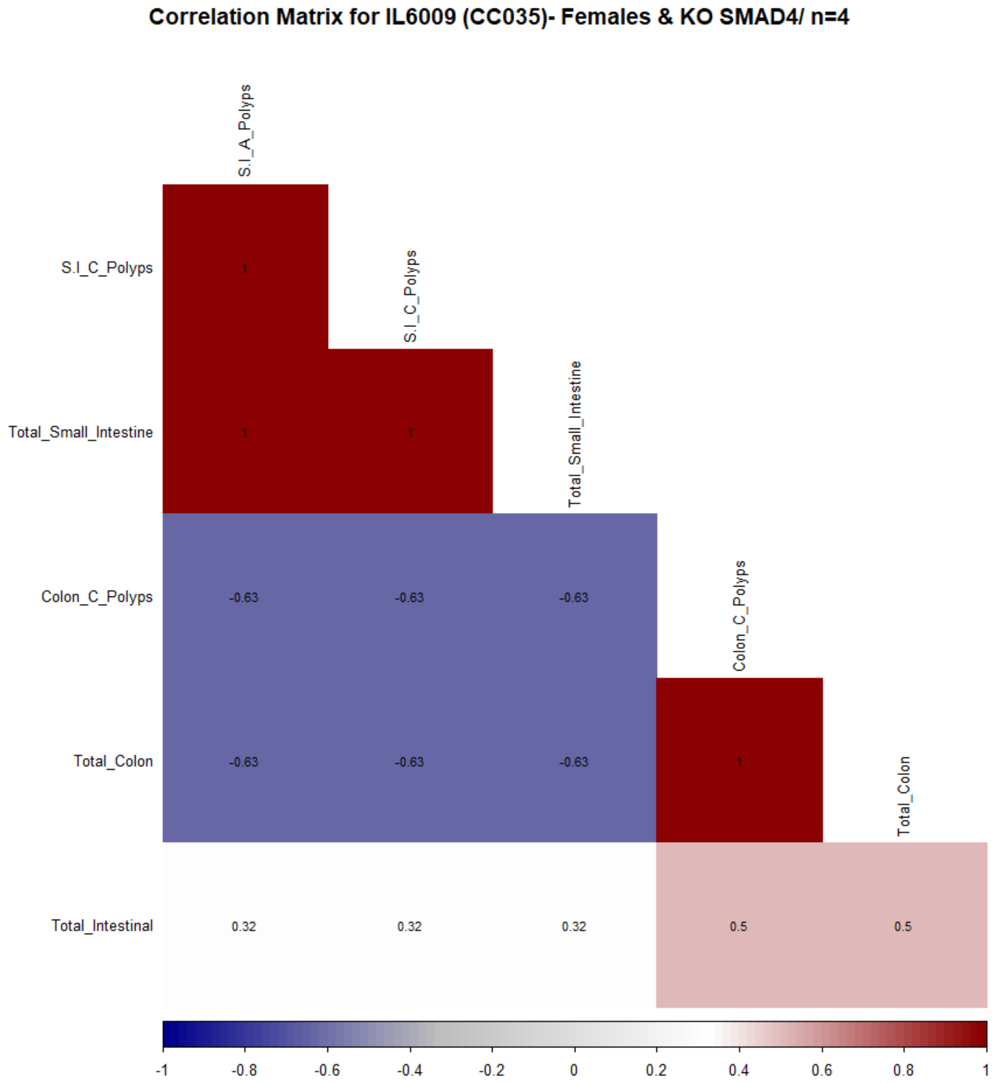

Supplementary Figure S68 Correlation Analysis of Polyp Development Patterns in HETEROZYGOUS KNOCK-OUT female Mice from line CC035.

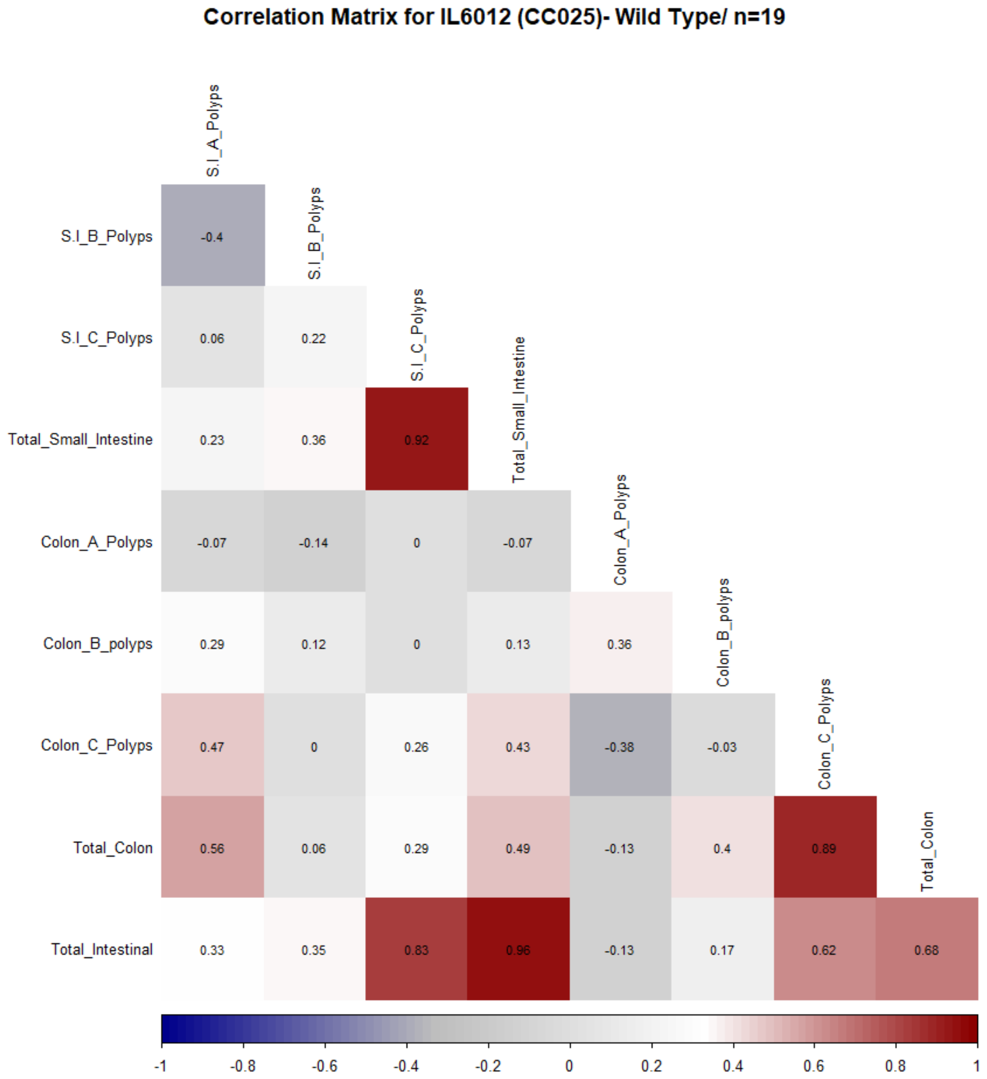

Supplementary Figure S69 Correlation Analysis of Polyp Development Patterns in Wild-Type Mice from line CC025.

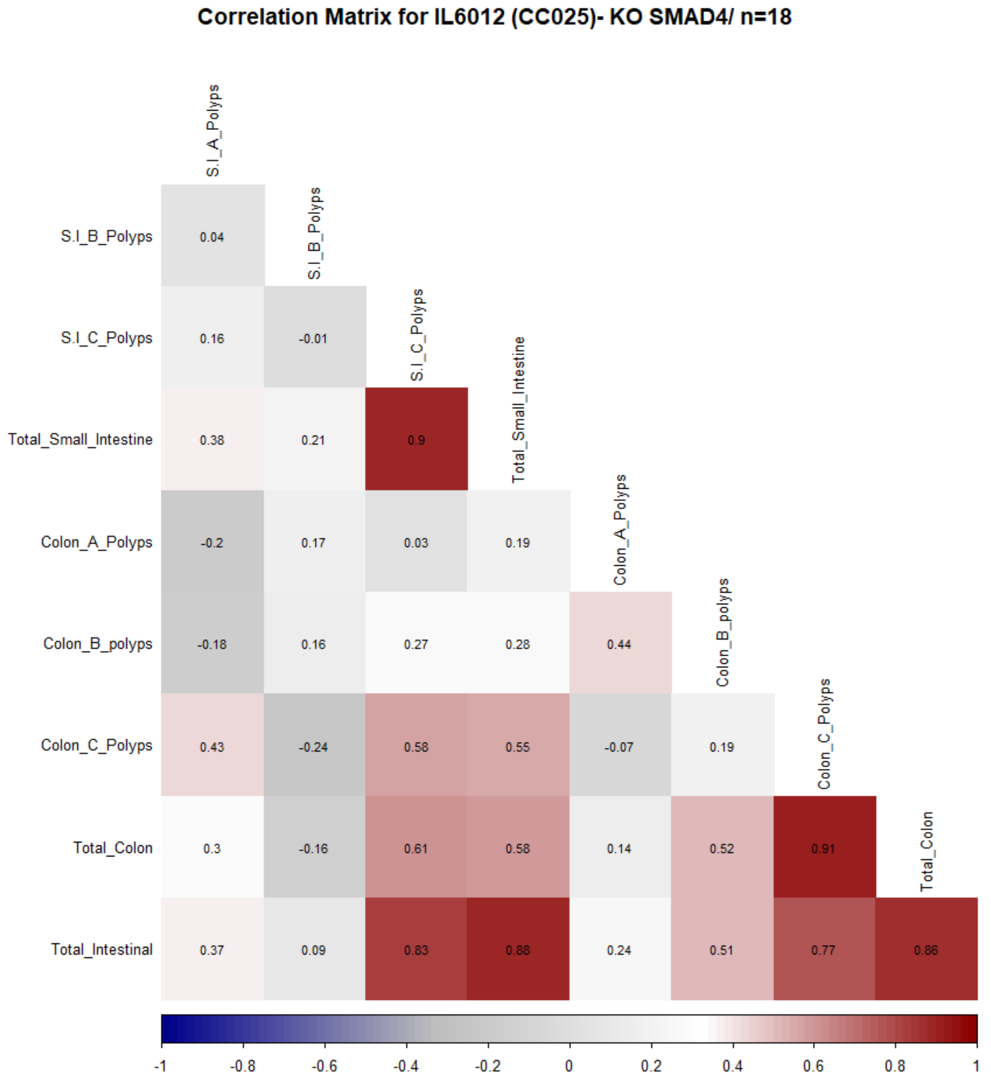

Supplementary Figure S70 Correlation Analysis of Polyp Development Patterns in HETEROZYGOUS KNOCK-OUT Mice from line CC025.

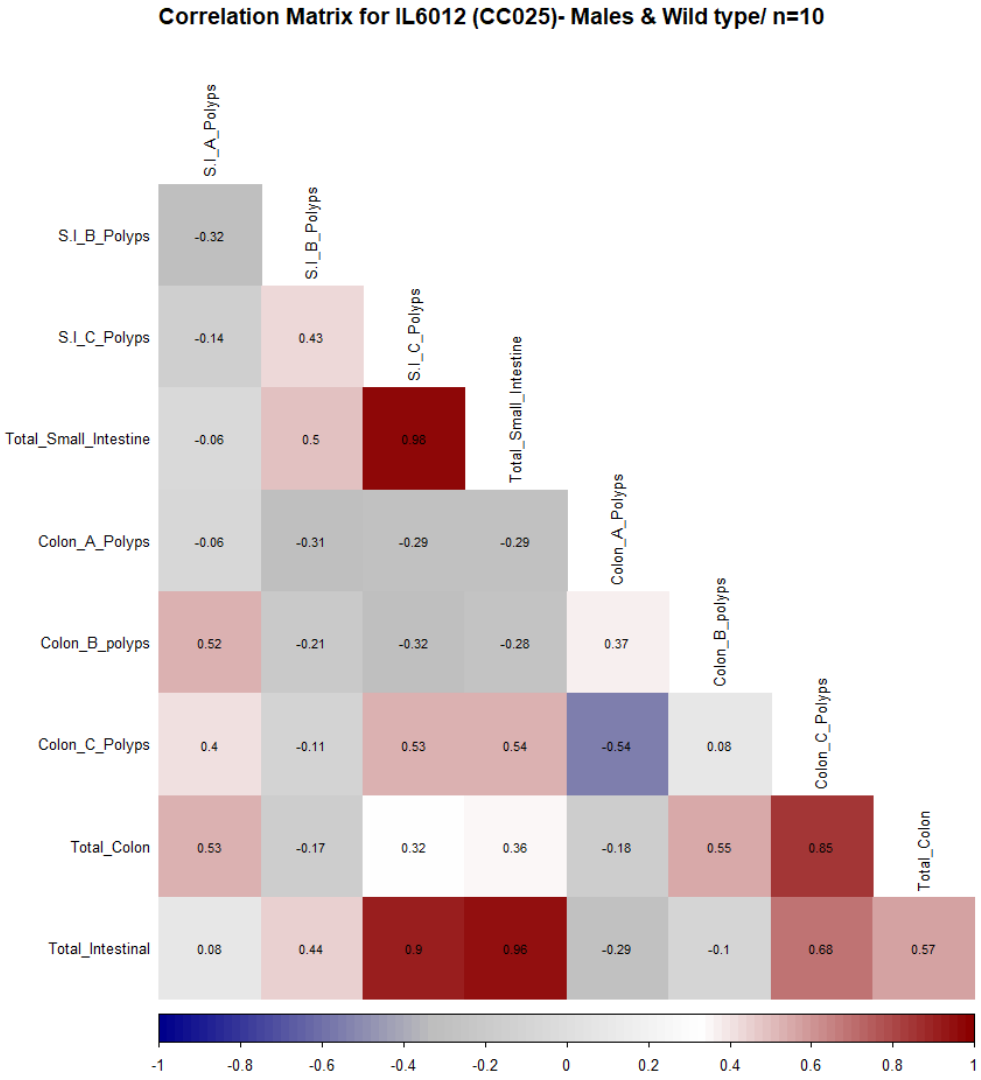

Supplementary Figure S71 Correlation Analysis of Polyp Development Patterns in Wild-Type male Mice from line CC025.

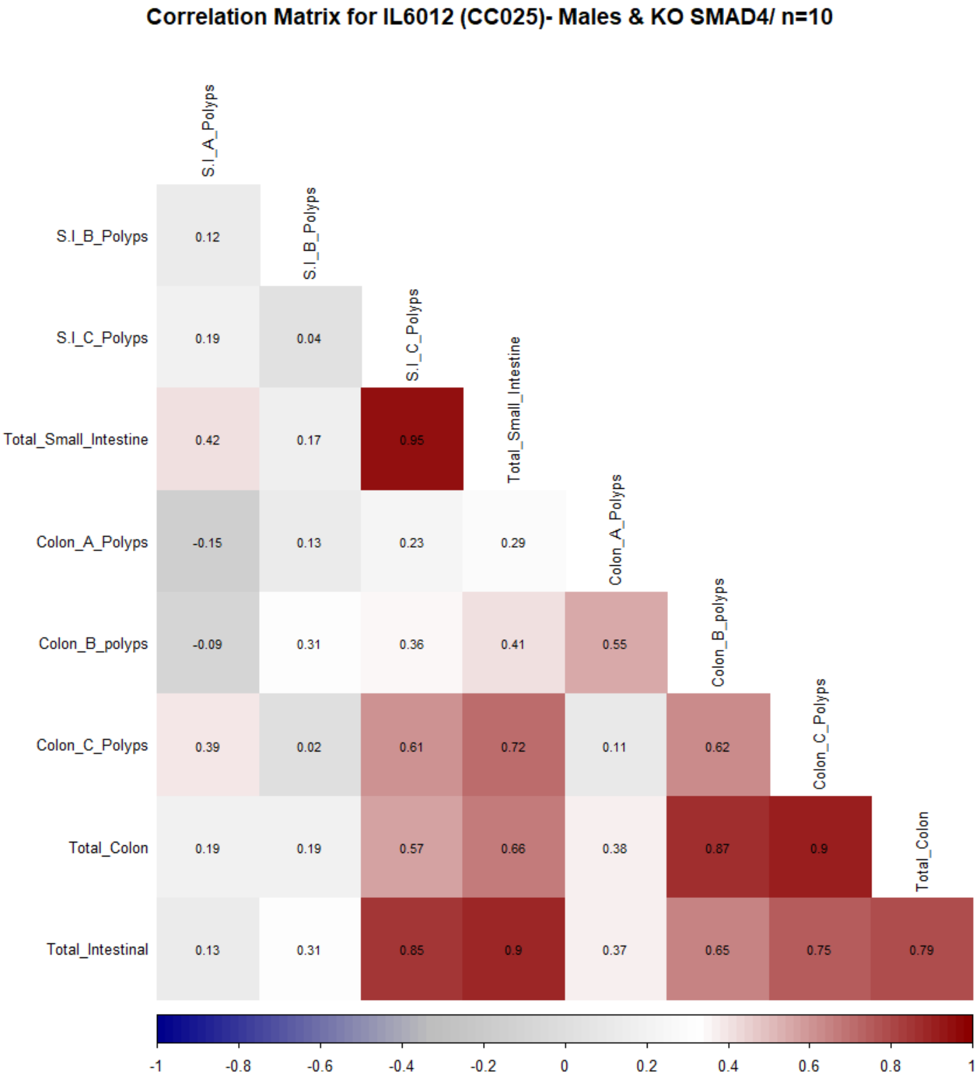

Supplementary Figure S72 Correlation Analysis of Polyp Development Patterns in HETEROZYGOUS KNOCK-OUT male Mice from line CC025.

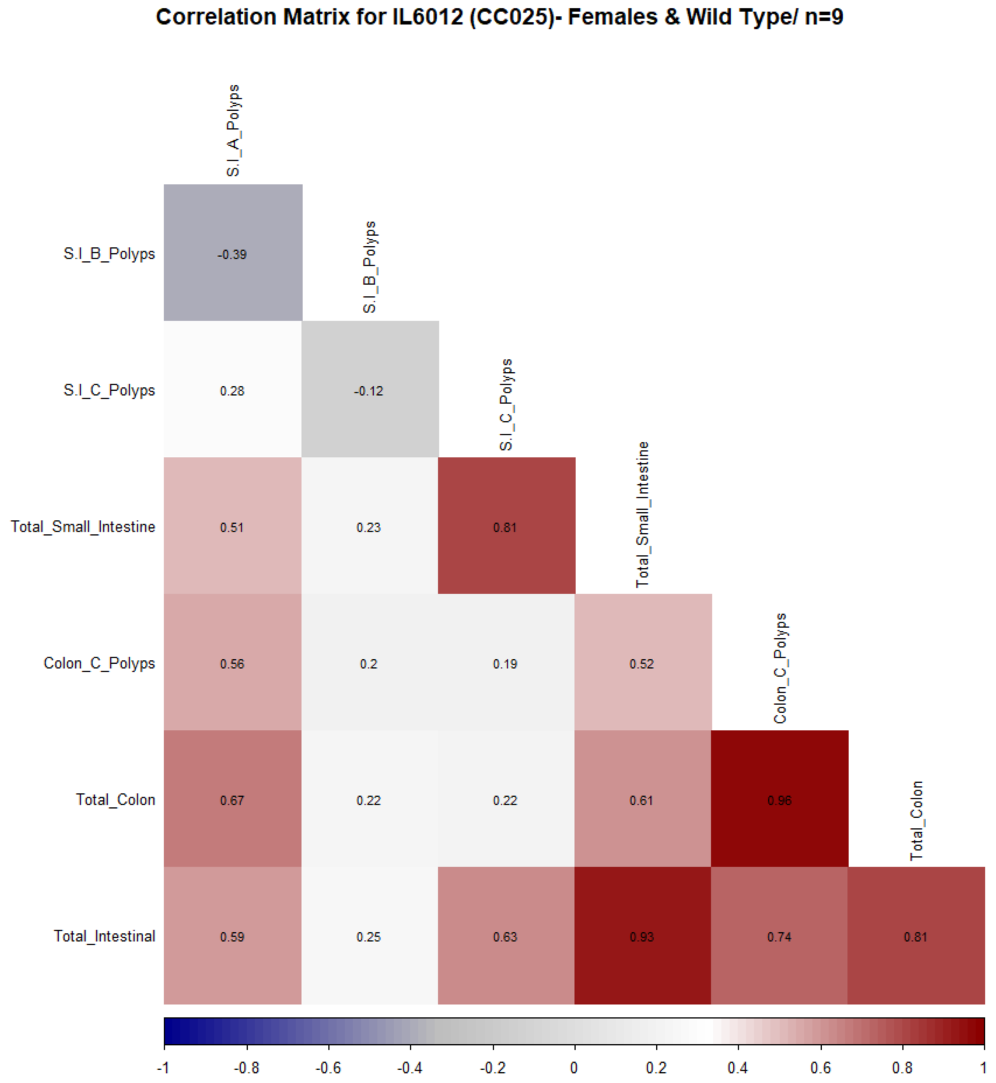

Supplementary Figure S73 Correlation Analysis of Polyp Development Patterns in Wild-Type female Mice from line CC025.

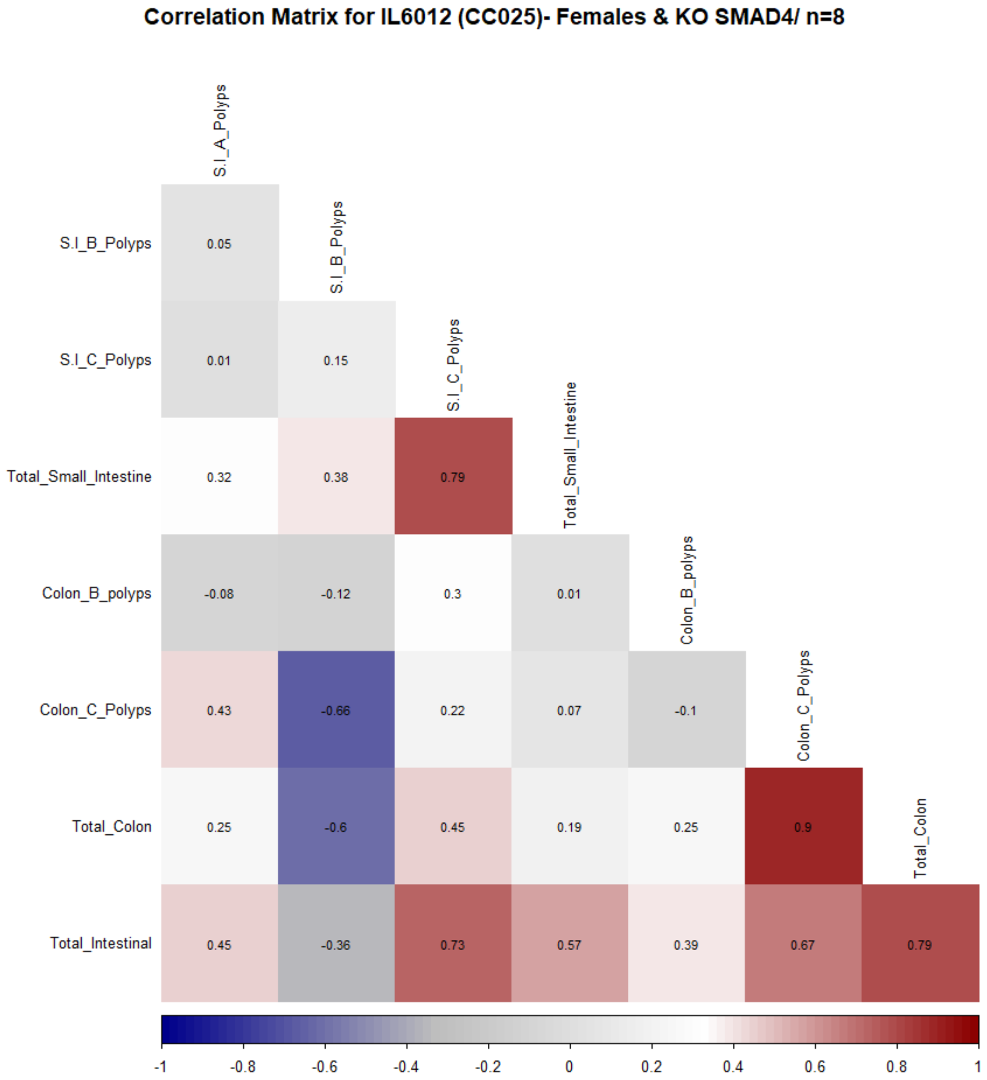

Supplementary Figure S74 Correlation Analysis of Polyp Development Patterns in HETEROZYGOUS KNOCK-OUT female Mice from line CC025.

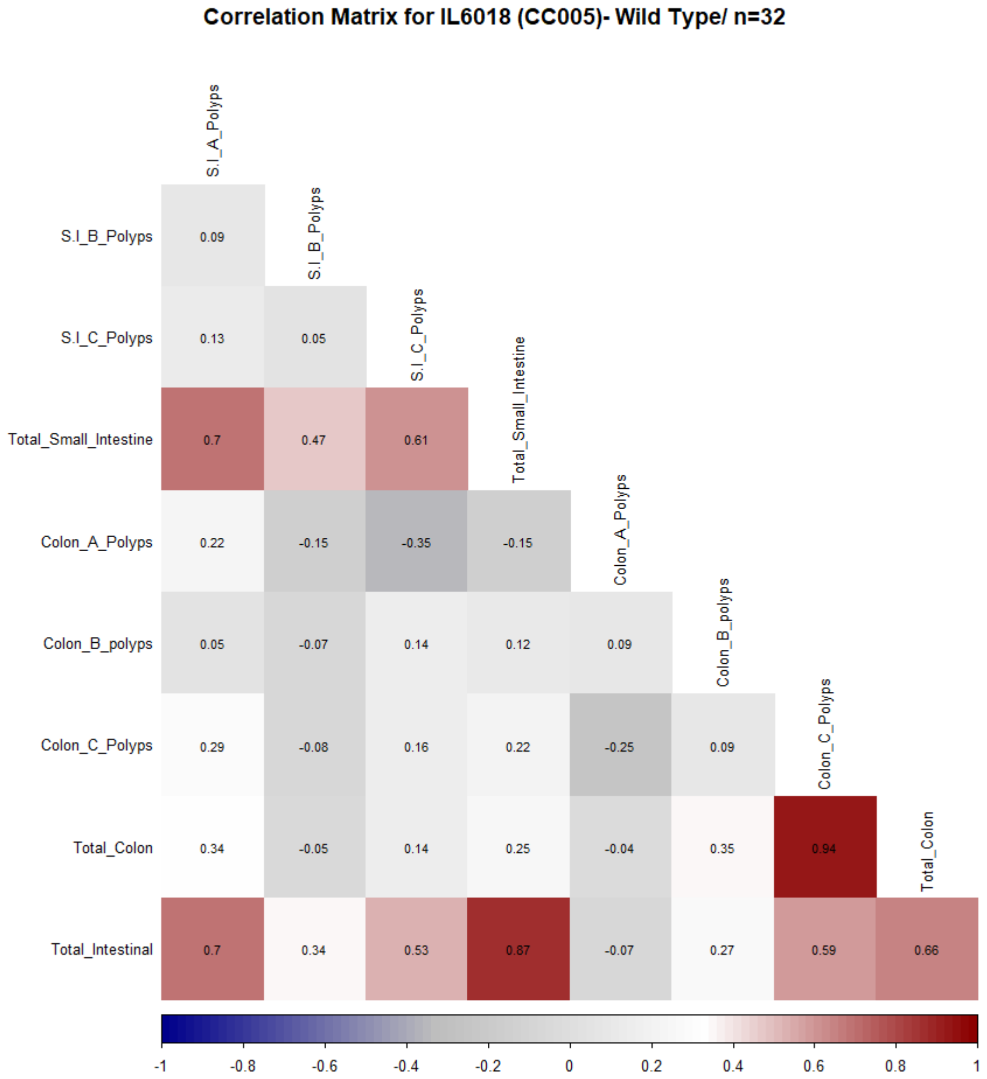

Supplementary Figure S75 Correlation Analysis of Polyp Development Patterns in Wild-Type Mice from line CC005.*

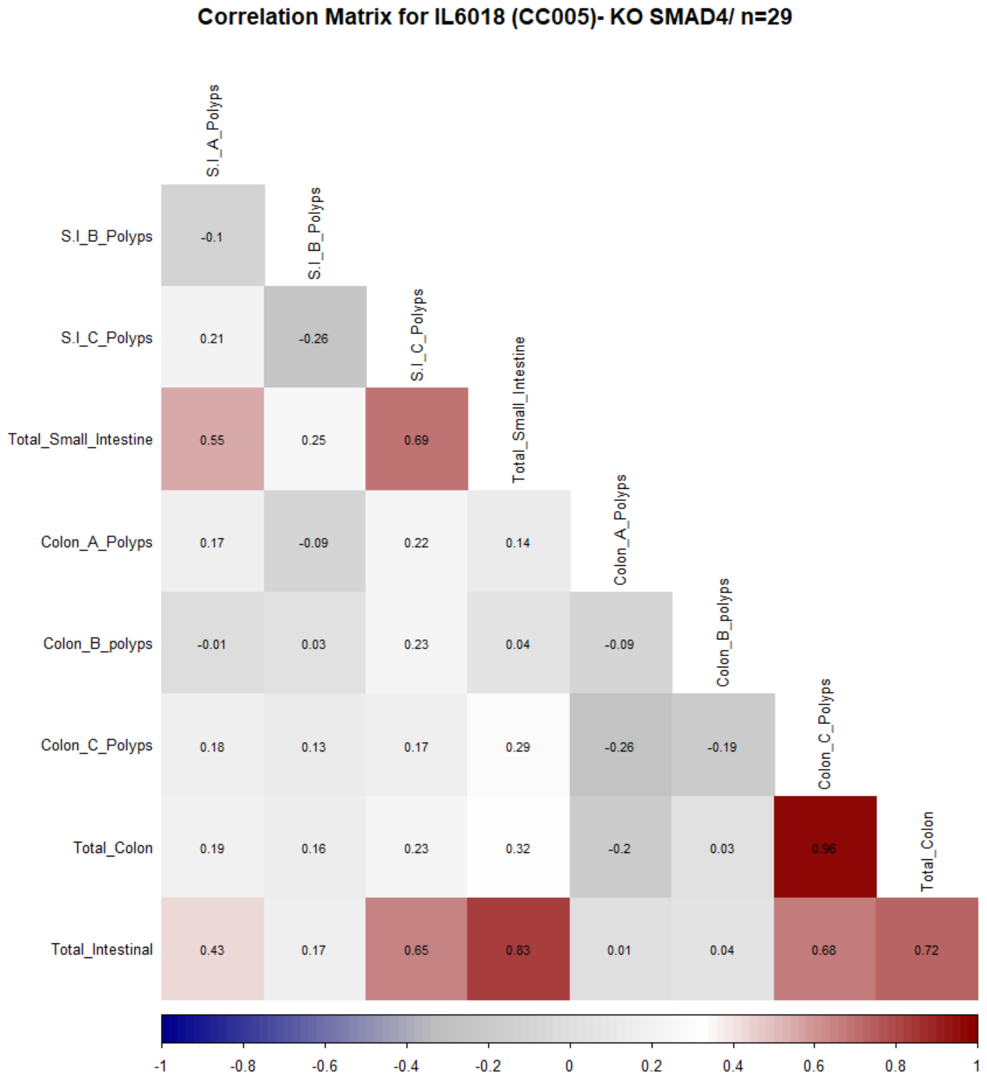

Supplementary Figure S76 Correlation Analysis of Polyp Development Patterns in HETEROZYGOUS KNOCK-OUT Mice from line CC005.*

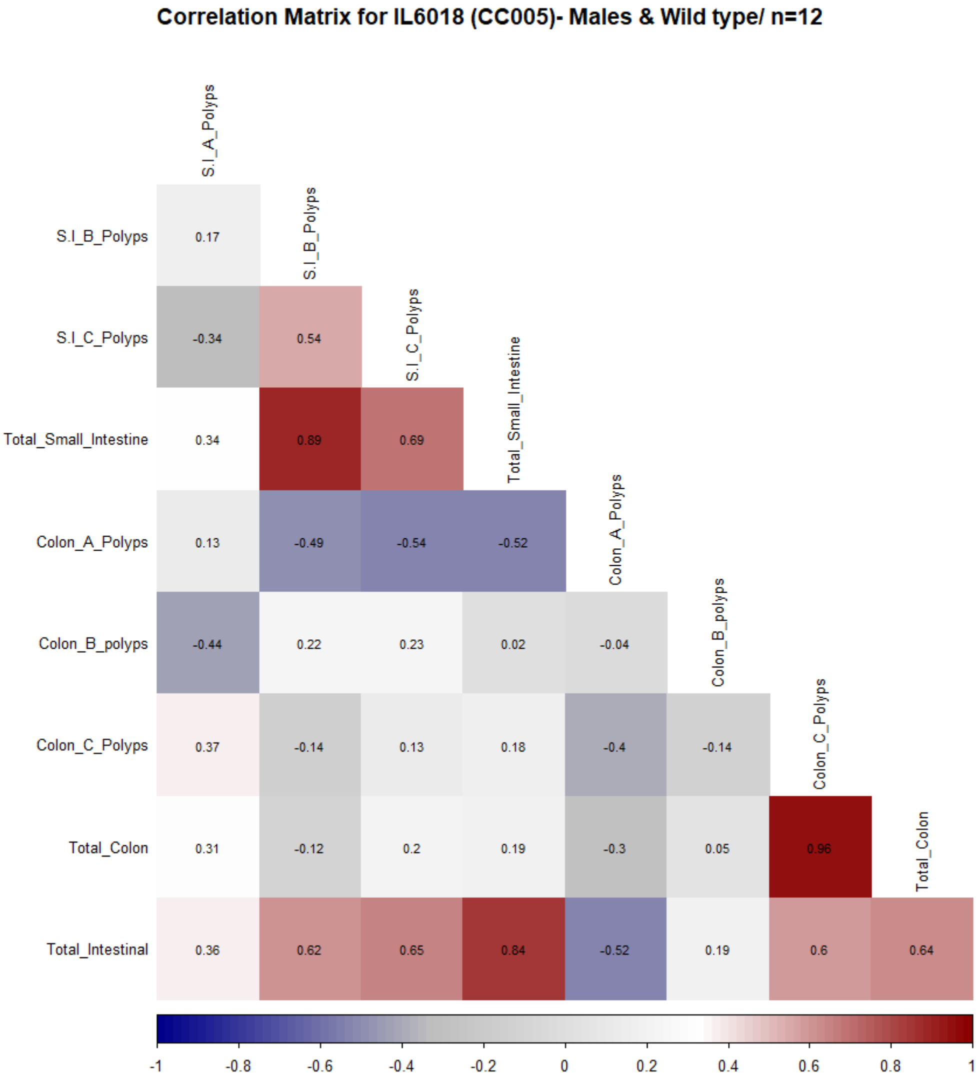

Supplementary Figure S77 Correlation Analysis of Polyp Development Patterns in Wild-Type male Mice from line CC005.*

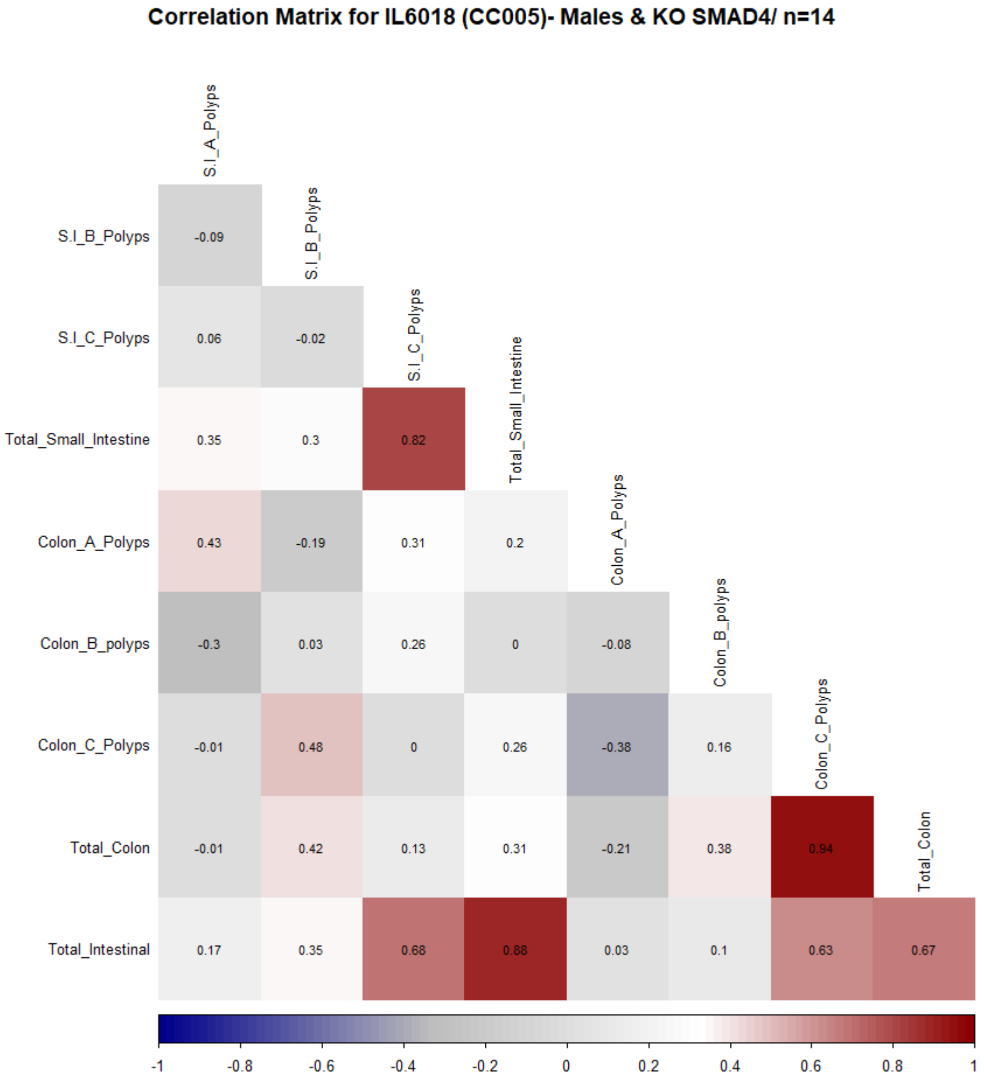

Supplementary Figure S78 Correlation Analysis of Polyp Development Patterns in HETEROZYGOUS KNOCK-OUT male Mice from line CC005.*

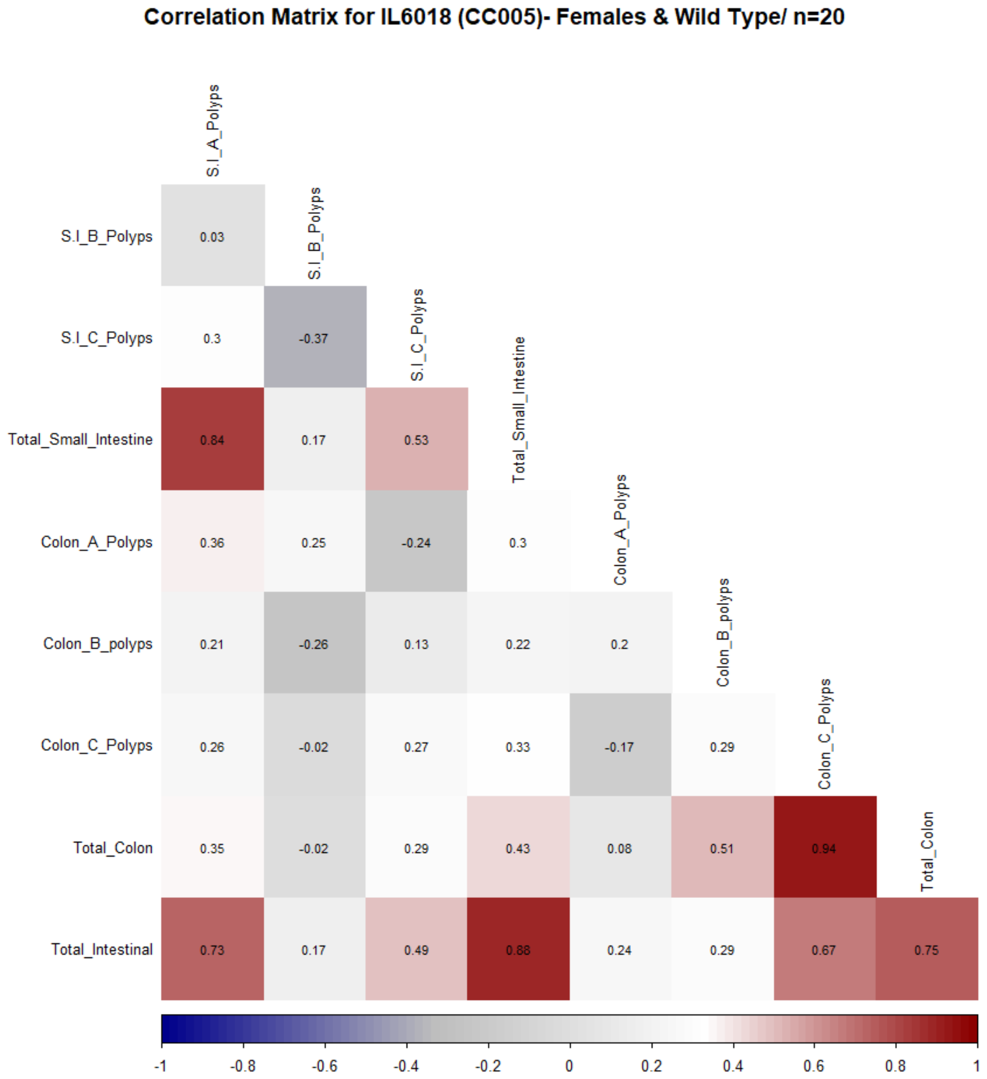

Supplementary Figure S79 Correlation Analysis of Polyp Development Patterns in Wild-Type female Mice from line CC005.*

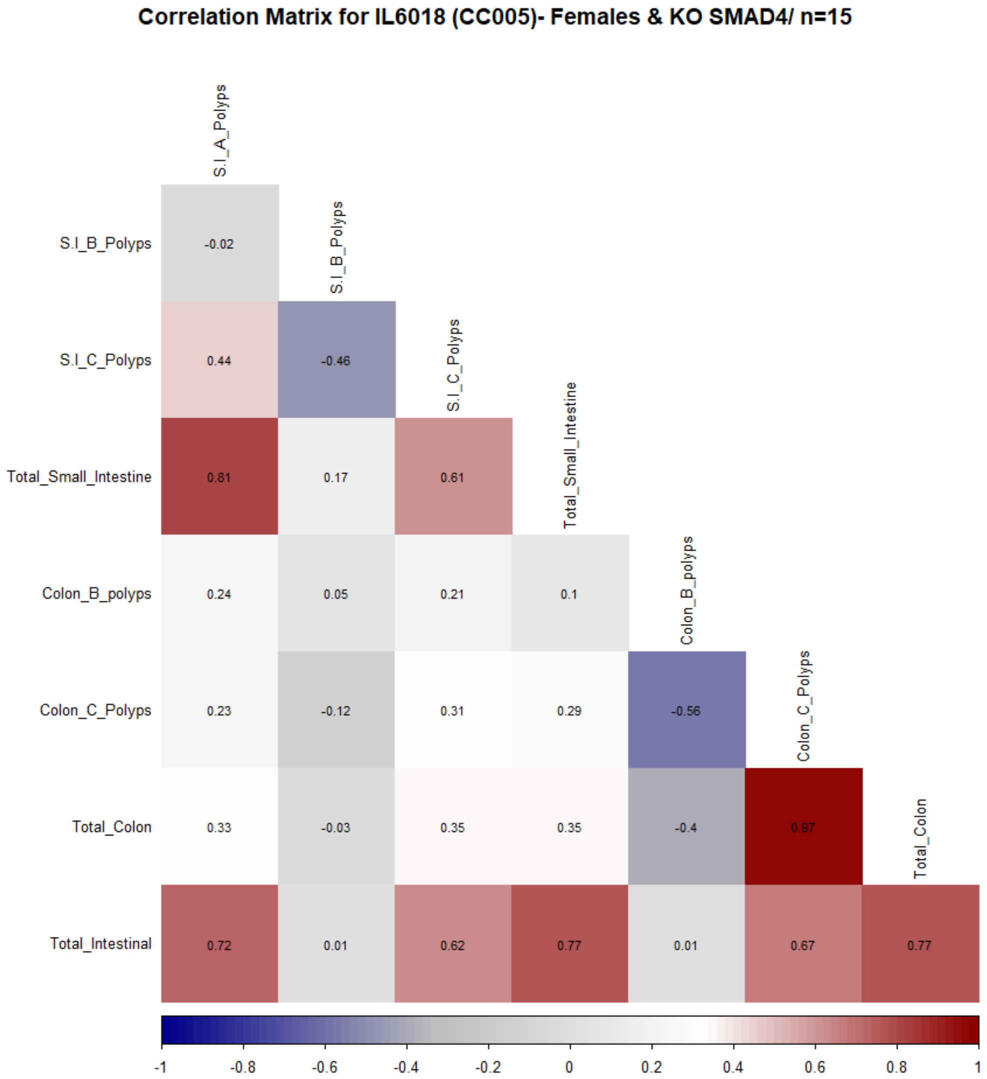

Supplementary Figure S80 Correlation Analysis of Polyp Development Patterns in HETEROZYGOUS KNOCK-OUT female Mice from line CC005.*
